# Supplementary material for: Optogenetic activation of local colonic sympathetic innervations attenuates colitis by limiting immune cell extravasation
Source: Immunity. 2021 May 11;54(5):1022–1036.e8. doi: 10.1016/j.immuni.2021.04.007 (PMC8116309; doi:10.1016/j.immuni.2021.04.007)
Supplement: Document S1. Figures S1–S5 [file mmc1.pdf]

Immunity, Volume *54*

Supplemental information

Optogenetic activation of local colonic sympathetic innervations attenuates colitis by limiting immune cell extravasation

Maya Schiller, Hilla Azulay-Debby, Nadia Boshnak, Yehezqel Elyahu, Ben Korin, Tamar L. Ben-Shaanan, Tamar Koren, Maria Krot, Fahed Hakim, and Asya Rolls

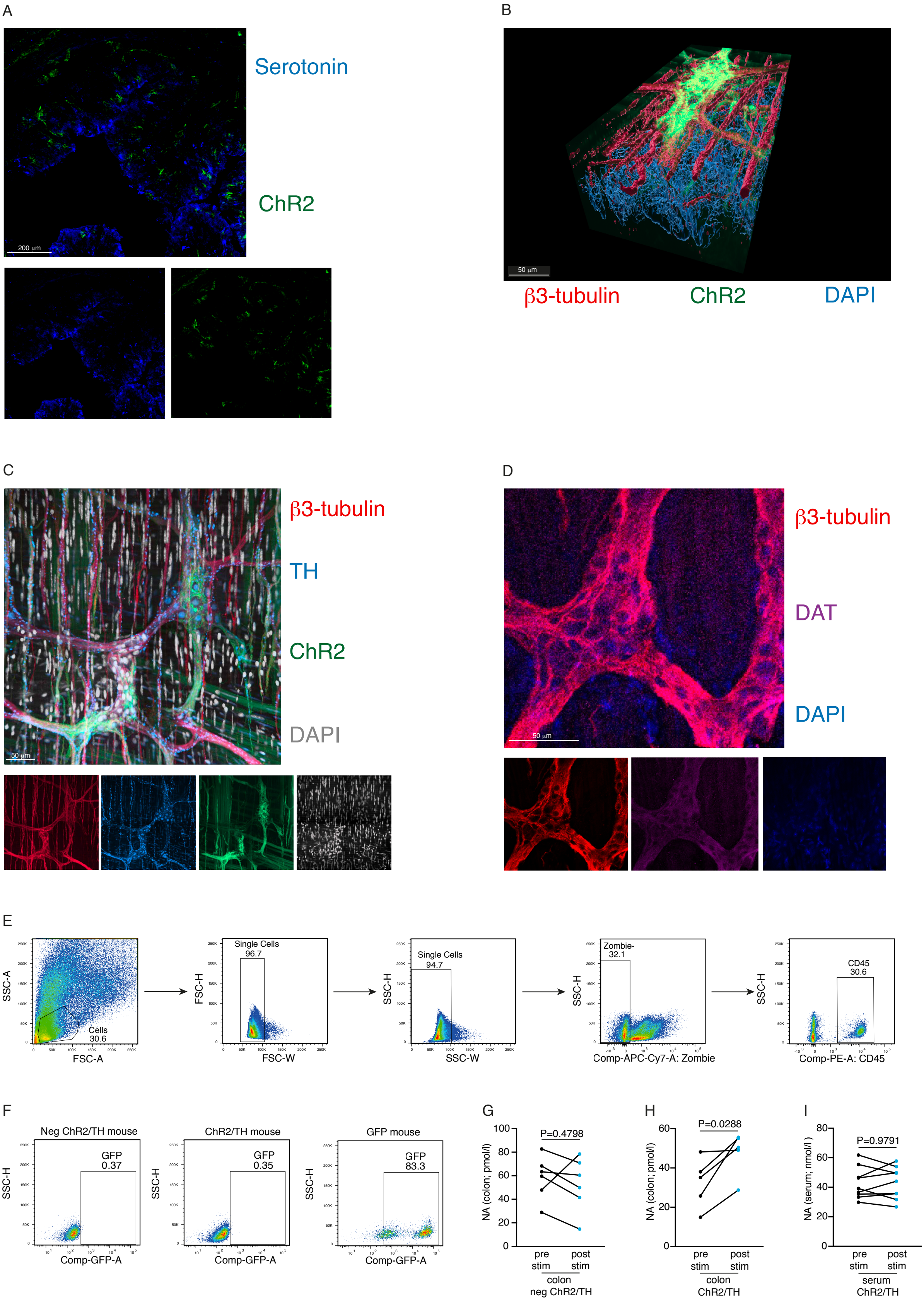

A
Serotonin
ChR2
200 µm
B
50 µm
β3-tubulin
ChR2
DAPI
C
β3-tubulin
TH
ChR2
DAPI
50 µm
D
β3-tubulin
DAT
DAPI
50 µm
E
SSC-A
FSC-A
Cells
30.6
FSC-H
FSC-W
Single Cells
96.7
SSC-H
SSC-W
Single Cells
94.7
SSC-H
Comp-APC-Cy7-A: Zombie
Zombie-
32.1
SSC-H
Comp-PE-A: CD45
CD45
30.6
F
Neg ChR2/TH mouse
GFP
0.37
ChR2/TH mouse
GFP
0.35
GFP mouse
GFP
83.3
SSC-H
Comp-GFP-A
G
NA (colon; pmol/l)
P=0.4798
pre stim
post stim
colon
neg ChR2/TH
H
NA (colon; pmol/l)
P=0.0288
pre stim
post stim
colon
ChR2/TH
I
NA (serum; nmol/l)
P=0.9791
pre stim
post stim
serum
ChR2/TH

Figure S1: Characterization of the optogenetic activation of local sympathetic fibers in the colon. Related to Figure 1. (A) Expression of the markers serotonin (blue) and the ChR2 fluorescent marker (green) in the colon of ChR2/TH mice, demonstrating that ChR2 is not express serotonin^{+} cells. Scale bar=200 µm. **(B)** Expression of the markers β3-tubulin (red), the ChR2 fluorescent marker (green) and DAPI (blue) in the colon of ChR2/TH mice. The image was taken from a colon that underwent a clearing technique. Scale bar=50 µm. **(C)** Expression of the markers β3-tubulin (red), TH (blue), ChR2 fluorescent marker (green) and DAPI (grey) in the colon of ChR2/TH mice. The image was taken from a colon that underwent a clearing technique. Scale bar=50 µm. **(D)** Expression of the markers β3-tubulin (red), DAT (purple), and DAPI (blue) in the colon of C57BL/6 mice. Scale bar=50 µm. **(E)** Gating strategy for ChR2/TH mice, their transgene negative littermates, and GFP mice used to validate that CD45^{+} cells from the colons of ChR2/TH mice do not express the ChR2 fluorescent marker. **(F)** Representative image of flow cytometry analysis, showing the percentage of GFP^{+} cells (the fluorescent marker expressed by ChR2^{+} cells) out of CD45^{+} cells (gating strategy shown in S1E) in the colons of negative littermates who do not express the ChR2 channel, ChR2/TH, and GFP mice as a positive control group. This analysis demonstrates that the ChR2 fluorescent marker was not expressed by CD45^{+} cells in the ChR2/TH mice. **(G)** Representative set of raw data of NA levels measured in the colons of transgene negative littermates and **(H)** ChR2/TH mice before (pre-stim) and after (post-stim) optogenetic stimulation. Negative littermate's colon: N=6; ChR2/TH colon: N=5. **(I)** NA levels measured from the serum of ChR2/TH mice before (pre-stim) and after (post- stim) optogenetic stimulation. N=9. Mean ± SEM, as well as individual mice, are presented for each group. Student's paired t-test.

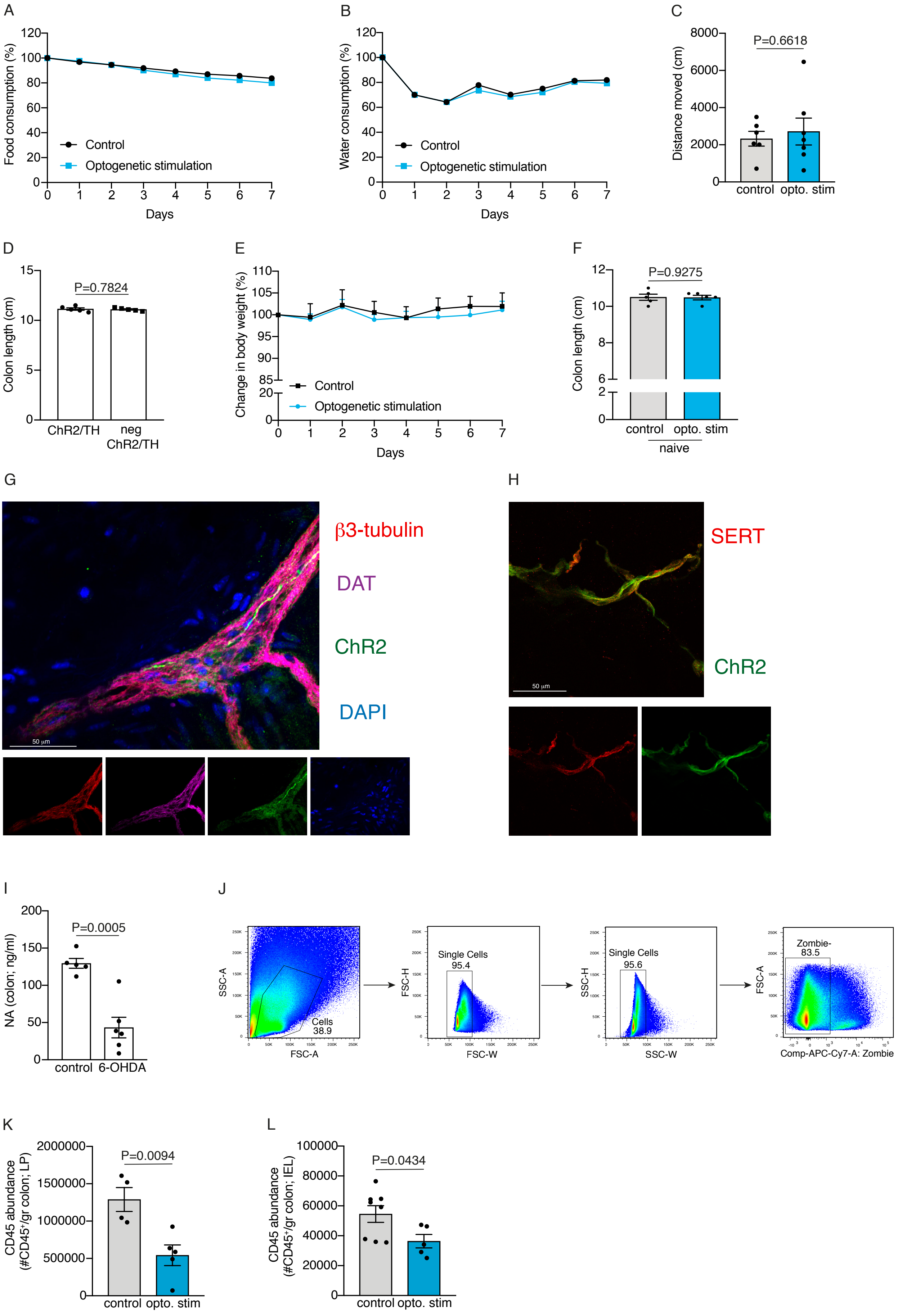

A
Food consumption (%)
Control
Optogenetic stimulation
Days
B
Water consumption (%)
Control
Optogenetic stimulation
Days
C
P=0.6618
Distance moved (cm)
control
opto. stim
D
P=0.7824
Colon length (cm)
ChR2/TH
neg ChR2/TH
E
Change in body weight (%)
Control
Optogenetic stimulation
Days
F
P=0.9275
Colon length (cm)
control
opto. stim
naive
G
β3-tubulin
DAT
ChR2
DAPI
50 μm
H
SERT
ChR2
50 μm
I
P=0.0005
NA (colon; ng/ml)
control
6-OHDA
J
SSC-A
FSC-A
Cells 38.9
FSC-H
FSC-W
Single Cells 95.4
SSC-H
SSC-W
Single Cells 95.6
FSC-A
Zombie- 83.5
Comp-APC-Cy7-A: Zombie
K
P=0.0094
CD45 abundance (#CD45+/gr colon; LP)
control
opto. stim
L
P=0.0434
CD45 abundance (#CD45+/gr colon; IEL)
control
opto. stim

Figure S2: Effects of optogenetic activation on immune abundance, behavioral and clinical traits in ChR2/TH mice, and the expression of ChR2 in ChR2/DAT and ChR2/SERT mice. Related to Figures 2 and 3. (A) Food and **(B)** water consumption by ChR2/TH mice and their controls (negative littermates exposed to light stimulation) during the 7 days of DSS administration and daily optogenetic activation. **(C)** Locomotion activity of ChR2/TH mice and their controls (negative littermates exposed to light stimulation) during the 7 days of DSS administration and daily optogenetic activation. The locomotion was calculated as the distance moved by each mouse (see methods). N=6, 7. **(D)** Colon length of ChR2/TH mice and their transgene negative littermates showing there is no difference in baseline colon length between the two strains. N=5, 5. **(E)** Percentage change in weight during the 7 days of daily optogenetic activation in naïve (not exposed to DSS) ChR2/TH mice and their controls (negative littermates exposed to light stimulation). N=5, 5. **(F)** Evaluation of colon length in naïve (not exposed to DSS) ChR2/TH mice and their controls (negative littermates exposed to light stimulation) following 7 days of daily optogenetic activation. N=5, 5. **(G)** Expression of the markers β3-tubulin (red), DAT (purple), ChR2 fluorescent marker (green) and DAPI (blue) in the colon of ChR2/DAT mice, demonstrating the expression of the ChR2 channel in DAT^+ neurons. Scale bar=50 μm. **(H)** Expression of the markers SERT (red) and the ChR2 fluorescent marker (green) in the colon of ChR2/SERT mice, demonstrating the expression of the ChR2 channel in $SERT^+$ cells. Scale bar=50 μm. **(I)** NA levels measured in the colons of mice injected IP with 6-OHDA (to ablate catecholamine neurons) and a vehicle-control group (injected with saline). N=5, 6. **(J)** Gating strategy for the analysis of immune cell abundance in the ChR2/TH mice and their controls. **(K)** Representative set of raw data of the abundance of immune cells (number of $CD45^+$ cells/gr colon) in the LP and **(L)** IEL layers of the colon from ChR2/TH mice and their controls following 7 days of 3% DSS and daily optogenetic activation. N=4, 5. Mean ± SEM as well as individual mice are presented for each group. Student's unpaired t-test.

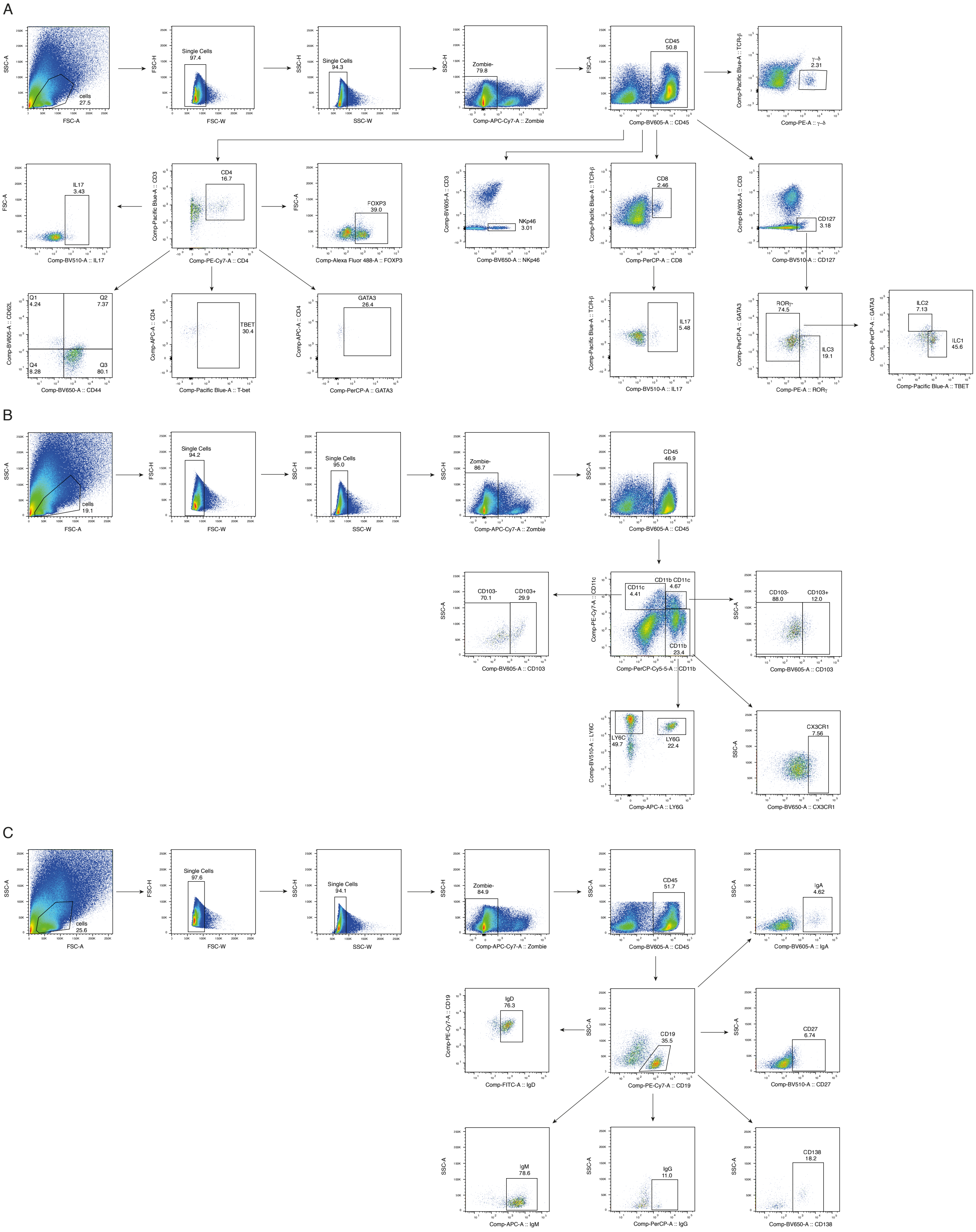
A
SSC-A
FSC-A
cells
27.5
FSC-H
FSC-W
Single Cells
97.4
SSC-H
SSC-W
Single Cells
94.3
Zombie-
79.8
Comp-APC-Cy7-A :: Zombie
CD45
50.8
Comp-BV605-A :: CD45
Comp-Pacific Blue-A :: TCR-β
γ-δ
2.31
Comp-PE-A :: γ-δ
IL17
3.43
Comp-BV510-A :: IL17
Comp-Pacific Blue-A :: CD3
CD4
16.7
Comp-PE-Cy7-A :: CD4
FOXP3
39.0
Comp-Alexa Fluor 488-A :: FOXP3
Comp-BV605-A :: CD3
NKp46
3.01
Comp-BV650-A :: NKp46
CD8
2.46
Comp-PerCP-A :: CD8
CD127
3.18
Comp-BV510-A :: CD127
Comp-BV605-A :: CD62L
Q1
4.24
Q2
7.37
Q4
8.28
Q3
80.1
Comp-BV650-A :: CD44
Comp-APC-A :: CD4
TBET
30.4
Comp-Pacific Blue-A :: T-bet
GATA3
26.4
Comp-PerCP-A :: GATA3
IL17
5.48
Comp-PerCP-A :: GATA3
RORγ
74.5
ILC3
19.1
Comp-PE-A :: RORγ
ILC2
7.13
ILC1
45.6
Comp-Pacific Blue-A :: TBET
B
cells
19.1
Single Cells
94.2
Single Cells
95.0
Zombie-
86.7
CD45
46.9
CD103-
70.1
CD103+
29.9
Comp-BV605-A :: CD103
Comp-PE-Cy7-A :: CD11c
CD11c
4.41
CD11b CD11c
4.67
CD11b
23.4
Comp-PerCP-Cy5-5-A :: CD11b
CD103-
88.0
CD103+
12.0
Comp-BV510-A :: LY6C
LY6C
49.7
LY6G
22.4
Comp-APC-A :: LY6G
CX3CR1
7.56
Comp-BV650-A :: CX3CR1
C
cells
25.6
Single Cells
97.6
Single Cells
94.1
Zombie-
84.9
CD45
51.7
IgA
4.62
Comp-BV605-A :: IgA
Comp-PE-Cy7-A :: CD19
IgD
76.3
Comp-FITC-A :: IgD
CD19
35.5
Comp-PE-Cy7-A :: CD19
CD27
6.74
Comp-BV510-A :: CD27
IgM
78.6
Comp-APC-A :: IgM
IgG
11.0
Comp-PerCP-A :: IgG
CD138
18.2
Comp-BV650-A :: CD138

Figure S3: Gating strategy for immune subpopulations analysis in ChR2/TH mice following optogenetic activation. Related to Figure 3. Gating strategy of **(A)** T cells, NK cells, ILCs **(B)** myeloid, dendritic cells and **(C**) B cells in the colon of ChR2/TH mice and their controls (negative littermates exposed to light stimulation) following 7 days of 3% DSS and daily optogenetic activation.

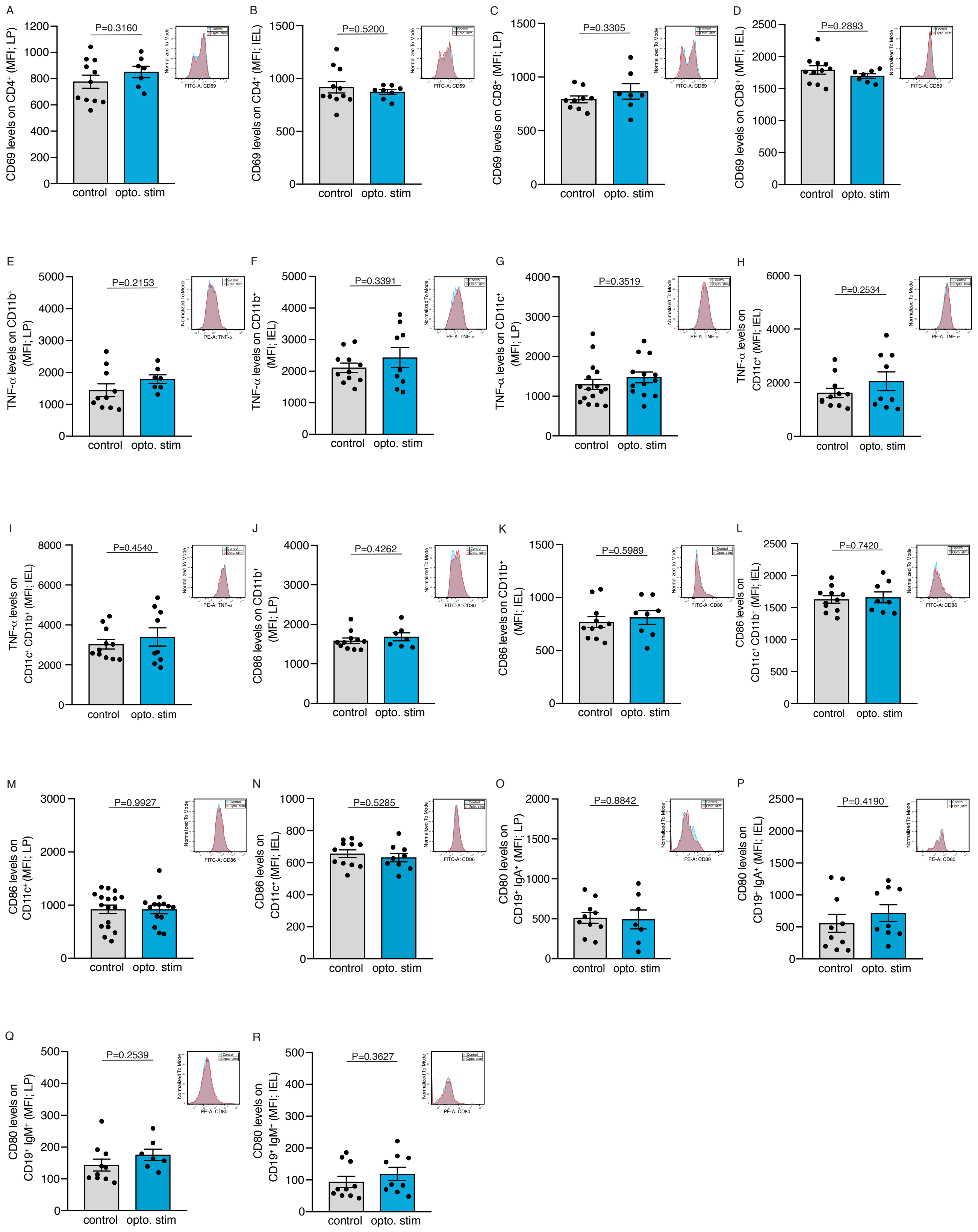
A
P=0.3160
CD69 levels on CD4+ (MFI; LP)
control
opto. stim
FITC-A: CD69
B
P=0.5200
CD69 levels on CD4+ (MFI; IEL)
control
opto. stim
FITC-A: CD69
C
P=0.3305
CD69 levels on CD8+ (MFI; LP)
control
opto. stim
FITC-A: CD69
D
P=0.2893
CD69 levels on CD8+ (MFI; IEL)
control
opto. stim
FITC-A: CD69
E
P=0.2153
TNF-α levels on CD11b+ (MFI; LP)
control
opto. stim
PE-A: TNF-α
F
P=0.3391
TNF-α levels on CD11b+ (MFI; IEL)
control
opto. stim
PE-A: TNF-α
G
P=0.3519
TNF-α levels on CD11c+ (MFI; LP)
control
opto. stim
PE-A: TNF-α
H
P=0.2534
TNF-α levels on CD11c+ (MFI; IEL)
control
opto. stim
PE-A: TNF-α
I
P=0.4540
TNF-α levels on CD11c+ CD11b+ (MFI; IEL)
control
opto. stim
PE-A: TNF-α
J
P=0.4262
CD86 levels on CD11b+ (MFI; LP)
control
opto. stim
FITC-A: CD86
K
P=0.5989
CD86 levels on CD11b+ (MFI; IEL)
control
opto. stim
FITC-A: CD86
L
P=0.7420
CD86 levels on CD11c+ CD11b+ (MFI; IEL)
control
opto. stim
FITC-A: CD86
M
P=0.9927
CD86 levels on CD11c+ (MFI; LP)
control
opto. stim
FITC-A: CD86
N
P=0.5285
CD86 levels on CD11c+ (MFI; IEL)
control
opto. stim
FITC-A: CD86
O
P=0.8842
CD80 levels on CD19+ IgA+ (MFI; LP)
control
opto. stim
PE-A: CD80
P
P=0.4190
CD80 levels on CD19+ IgA+ (MFI; IEL)
control
opto. stim
PE-A: CD80
Q
P=0.2539
CD80 levels on CD19+ IgM+ (MFI; LP)
control
opto. stim
PE-A: CD80
R
P=0.3627
CD80 levels on CD19+ IgM+ (MFI; IEL)
control
opto. stim
PE-A: CD80
Normalized To Mode

Figure S4: Flow cytometry analysis of functional markers in ChR2/TH mice following optogenetic activation. Related to Figure 3. This analysis demonstrates: Expression levels of CD69 (MFI) on **(A)** LP $CD4^+$ (N=11, 7) **(B)** IEL $CD4^+$ (N=11, 8) **(C)** LP $CD8^+$ (N=9, 7). **(D)** IEL $CD8^+$ (N=11, 7). Expression levels of TNF-α (MFI) on **(E)** LP $CD11b^+$ (N=10, 7) **(F)** IEL $CD11b^+$ (N=11, 9) **(G)** LP $CD11c^+$ (N=16, 13) **(H)** IEL $CD11c^+$ (N=11, 9) **(I)** IEL $CD11c^+$ $CD11b^+$ (N=11, 9). Expression levels of CD86 (MFI) on **(J)** LP $CD11b^+$ (N=11, 7) **(K)** IEL $CD11b^+$ (N=11, 8) **(L)** IEL $CD11c^+$ $CD11b^+$ (N=11, 8) **(M)** LP $CD11c^+$ (N=17, 14) **(N)** IEL $CD11c^+$ (N=11, 9). Expression levels of CD80 (MFI) on **(O)** LP $CD19^+$ IgA^+ (N=10, 7) **(P)** IEL $CD19^+$ IgA^+ (N=10, 9) **(Q)** LP $CD19^+$ IgM^+ (N=10, 7) **(R)** IEL $CD19^+$ IgM^+ (N=10, 9). The analysis was performed on the colons of ChR2/TH mice and their controls (negative littermates exposed to light stimulation) following 7 days of 3% DSS and daily optogenetic activation. In each figure the right panel is a representative flow cytometry histogram. Mean ± SEM as well as individual mice are presented for each group. Student's unpaired t-test.

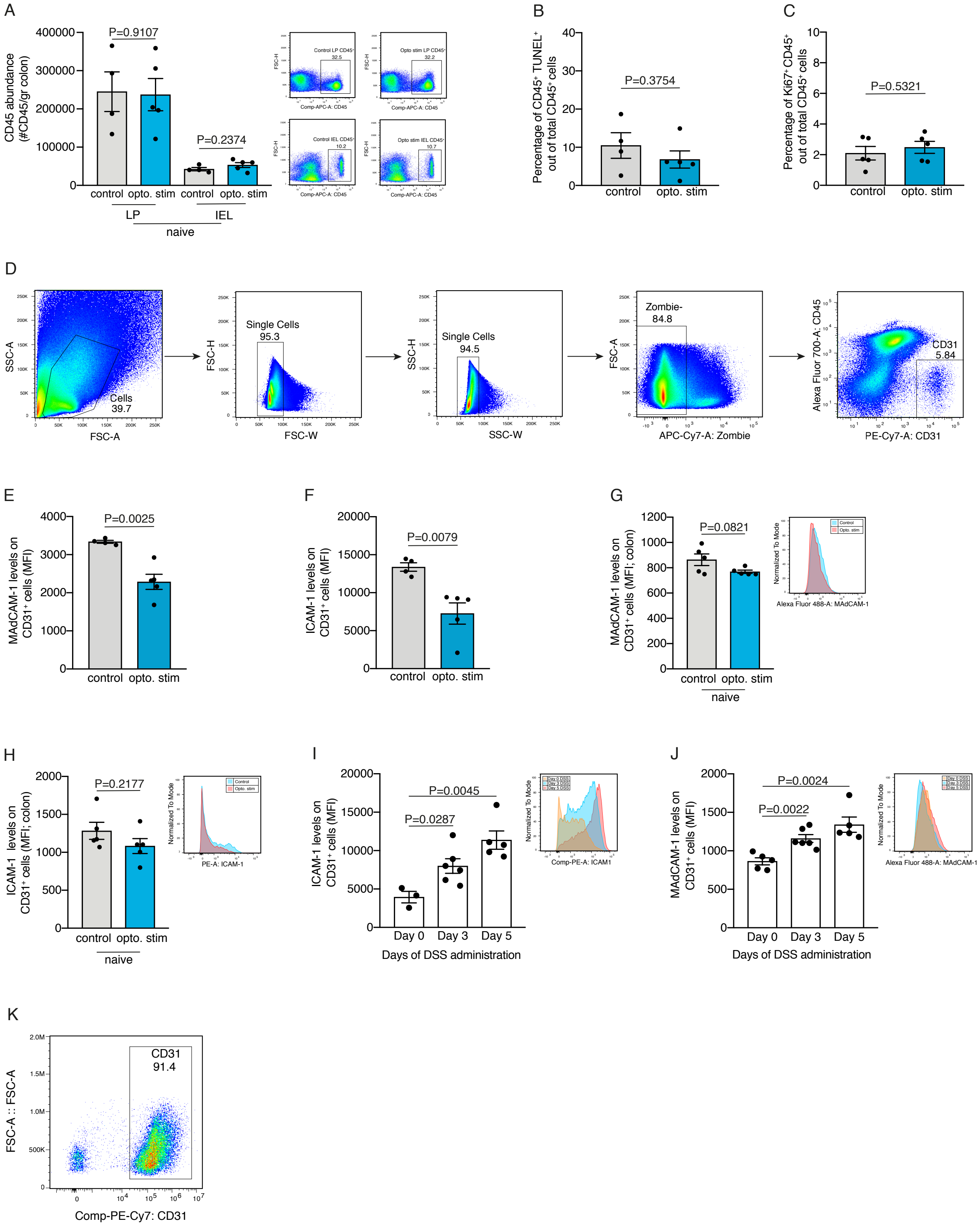
A
CD45 abundance (#CD45/gr colon)
400000
300000
200000
100000
0
P=0.9107
P=0.2374
control
opto. stim
control
opto. stim
LP
IEL
naive
Control LP CD45+ 32.5
Opto stim LP CD45+ 32.2
Control IEL CD45+ 10.2
Opto stim IEL CD45+ 10.7
Comp-APC-A: CD45
B
Percentage of CD45+ TUNEL+ out of total CD45+ cells
P=0.3754
control
opto. stim
C
Percentage of Ki67+ CD45+ out of total CD45+ cells
P=0.5321
control
opto. stim
D
SSC-A
FSC-A
Cells 39.7
FSC-H
FSC-W
Single Cells 95.3
SSC-H
SSC-W
Single Cells 94.5
FSC-A
APC-Cy7-A: Zombie
Zombie- 84.8
Alexa Fluor 700-A: CD45
PE-Cy7-A: CD31
CD31 5.84
E
MAdCAM-1 levels on CD31+ cells (MFI)
P=0.0025
control
opto. stim
F
ICAM-1 levels on CD31+ cells (MFI)
P=0.0079
control
opto. stim
G
MAdCAM-1 levels on CD31+ cells (MFI; colon)
P=0.0821
control
opto. stim
naive
Normalized To Mode
Alexa Fluor 488-A: MAdCAM-1
H
ICAM-1 levels on CD31+ cells (MFI; colon)
P=0.2177
control
opto. stim
naive
Normalized To Mode
PE-A: ICAM-1
I
ICAM-1 levels on CD31+ cells (MFI)
P=0.0045
P=0.0287
Day 0
Day 3
Day 5
Days of DSS administration
Normalized To Mode
Comp-PE-A: ICAM1
J
MAdCAM-1 levels on CD31+ cells (MFI)
P=0.0024
P=0.0022
Day 0
Day 3
Day 5
Days of DSS administration
Normalized To Mode
Alexa Fluor 488-A: MAdCAM-1
K
FSC-A :: FSC-A
CD31 91.4
Comp-PE-Cy7: CD31

Figure S5: Characterization of apoptosis and proliferation markers, immune abundance, MAdCAM-1 and ICAM-1 levels in ChR2/TH mice following optogenetic activation. Related to Figure 4. (A) Left: Representative set of raw data of abundance of immune cells (number of $CD45^+$ cells/gr colon) in the LP and IEL layers of the colon from naïve (not exposed to DSS) ChR2/TH mice and their controls (negative littermates exposed to light stimulation) following 7 days of daily optogenetic activation. Right: Representative flow cytometry plots demonstrating the percentage of $CD45^+$ population in the LP and IEL layers. N=4, 5. **(B)** Immunohistochemistry analysis of the percentage of $CD45^+$ cells expressing TUNEL (apoptosis marker) out of total $CD45^+$ cells in the colon of ChR2/TH mice and their controls (negative littermates exposed to light stimulation) following 7 days of 3% DSS and daily optogenetic activation. N=4, 5. **(C)** Immunohistochemistry analysis of the percentage of $CD45^+$ cells expressing Ki67 (proliferation marker) out of total $CD45^+$ cells in the colon of ChR2/TH mice and their controls (negative littermates exposed to light stimulation) following 7 days of 3% DSS and daily optogenetic activation. N=5, 5. **(D)** Gating strategy for the analysis of MAdCAM-1 and ICAM-1 expression level on endothelial cells ($CD31^+$ cells). **(E)** Representative set of raw data of flow cytometry analysis demonstrating the expression level of MAdCAM-1 on endothelial cells ($CD31^+$; indicated by MFI) in the colons of ChR2/TH mice and their controls following 7 days of 3% DSS and daily optogenetic activation. N=4, 5. **(F)** Representative set of raw data of flow cytometry analysis demonstrating the expression level of ICAM-1 on endothelial cells ($CD31^+$; indicated by MFI) in the colons of ChR2/TH mice and their controls following 7 days of 3% DSS and daily optogenetic activation. N=4, 5. **(G)** Left: Flow cytometry analysis of MAdCAM-1 expression on endothelial cells ($CD31^+$; indicated by MFI) in the colons of naïve (not exposed to DSS) ChR2/TH mice and their controls (negative littermates exposed to light stimulation), following 7 days of daily optogenetic stimulation. Right: Representative flow cytometry histogram demonstrating MAdCAM-1 expression level on $CD31^+$ cells. N=5, 5. **(H)** Left: Flow cytometry analysis of ICAM-1 expression on endothelial cells ($CD31^+$; indicated by MFI) in the colons of naïve (not exposed to DSS) ChR2/TH mice and their controls (negative littermates exposed to light stimulation), following 7 days of daily optogenetic stimulation. Right: Representative flow cytometry histogram demonstrating ICAM-1 expression level on $CD31^+$ cells. N=5, 5. **(I)** Left: Flow cytometry analysis of ICAM-1 expression level on endothelial cells ($CD31^+$; indicated by MFI) in the colons of C57BL/6 mice at different time points during 3% DSS administration. N=3, 6, 5. Right: Representative flow cytometry histogram demonstrating the ICAM-1 expression level on $CD31^+$

cells. **(J)** Left: Flow cytometry analysis of MAdCAM-1 expression level on endothelial cells ($CD31^+$; indicated by MFI) in the colons of C57BL/6 mice at different time points during 3% DSS administration. N=5, 6, 5. Right: Representative flow cytometry histogram demonstrating the MAdCAM-1 expression level on $CD31^+$ cells. **(K)** Representative image of flow cytometry analysis, showing the percentage of colon-derived endothelial cells ($CD31^+$) following endothelial enrichment (see methods). Mean ± SEM as well as individual mice are presented for each group. Student's unpaired t-test.
