# Supplementary material for: Optogenetic activation of local colonic sympathetic innervations attenuates colitis by limiting immune cell extravasation
Source: Immunity. 2021 May 11;54(5):1022–1036.e8. doi: 10.1016/j.immuni.2021.04.007 (PMC8116309; doi:10.1016/j.immuni.2021.04.007)
Supplement: Document S2. Article plus Supplemental information [file mmc2.pdf]

Immunity

Article

Optogenetic activation of local colonic sympathetic innervations attenuates colitis by limiting immune cell extravasation

Graphical abstract

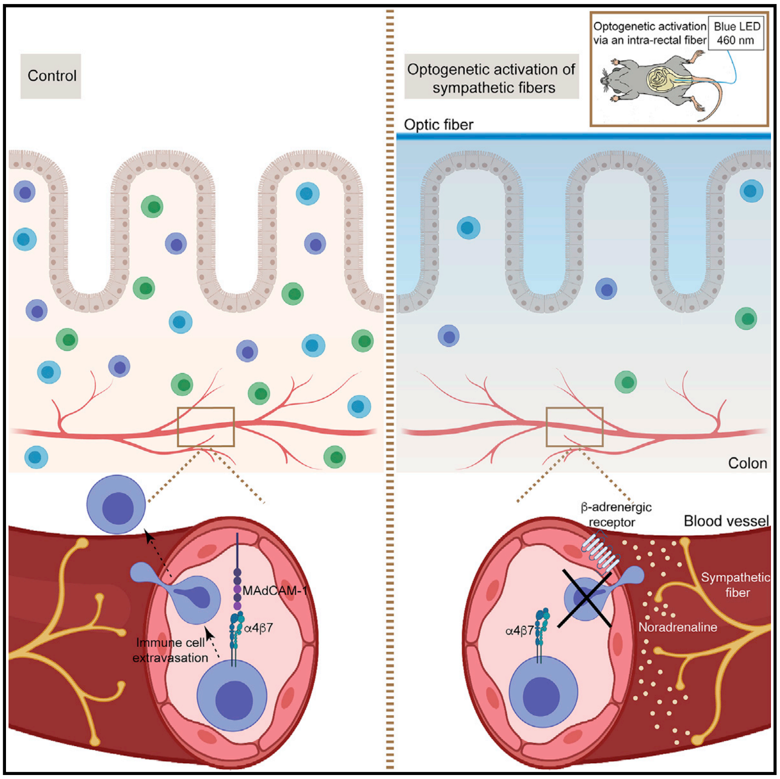

Highlights

- Optogenetic activation of local sympathetic fibers in the colon attenuates colitis
- The local sympathetic activation reduces immune cell abundance
- Reduction in immune abundance is mediated by a decrease in endothelial MAdCAM-1
- Endothelial MAdCAM-1 is regulated by noradrenaline via the β-adrenergic receptor

Authors

Maya Schiller, Hilla Azulay-Debby, Nadia Boshnak, ..., Maria Krot, Fahed Hakim, Asya Rolls

Correspondence

rolls.asya@gmail.com

In brief

Optogenetic activation of sympathetic fibers in the colon attenuated colitis and reduced immune cell abundance. This effect was mediated by endothelial cells, which reduced their expression of MAdCAM-1, required for immune extravasation. Thus, Schiller et al. introduce a mechanism whereby sympathetic fibers control the endothelial barrier to regulate local inflammation.

Schiller et al., 2021, Immunity 54, 1022–1036
May 11, 2021 © 2021 The Authors. Published by Elsevier Inc.
https://doi.org/10.1016/j.immuni.2021.04.007

CellPress

CellPress OPEN ACCESS

Immunity

Article

Optogenetic activation of local colonic sympathetic innervations attenuates colitis by limiting immune cell extravasation

Maya Schiller,[1,2,3] Hilla Azulay-Debby,[1,2,3] Nadia Boshnak,[1,2,3] Yehezqel Elyahu,[4] Ben Korin,[1,2,3] Tamar L. Ben-Shaanan,[1,2,3] Tamar Koren,[1,2,3] Maria Krot,[1,2,3] Fahed Hakim,[5,6] and Asya Rolls[1,2,3,7,*]

[1]Department of Immunology, Rappaport Faculty of Medicine, Technion—Israel Institute of Technology, 3525422, Haifa, Israel
[2]Department of Neuroscience, Rappaport Faculty of Medicine, Technion—Israel Institute of Technology, 3525422, Haifa, Israel
[3]The Technion Integrated Cancer Center, Technion—Israel Institute of Technology, 3525422, Haifa, Israel
[4]Department of Microbiology, Immunology and Genetics, Faculty of Health Sciences, Ben-Gurion University of the Negev, 8410501, Beer-Sheva, Israel
[5]Cancer Research Center, EMMS Nazareth, 16100, Nazareth, Israel
[6]Azrieli faculty of medicine, Bar-Ilan university, 1311502, Safad, Israel
[7]Lead contact
*Correspondence: rolls.asya@gmail.com
https://doi.org/10.1016/j.immuni.2021.04.007

SUMMARY

The sympathetic nervous system is composed of an endocrine arm, regulating blood adrenaline and noradrenaline, and a local arm, a network of fibers innervating immune organs. Here, we investigated the impact of the local arm of the SNS in an inflammatory response in the colon. Intra-rectal insertion of an optogenetic probe in mice engineered to express channelrhodopsin-2 in tyrosine hydroxylase cells activated colonic sympathetic fibers. In contrast to systemic application of noradrenaline, local activation of sympathetic fibers attenuated experimental colitis and reduced immune cell abundance. Gene expression profiling showed decreased endothelial expression of the adhesion molecule MAdCAM-1 upon optogenetic stimulation; this decrease was sensitive to adrenergic blockers and 6-hydroxydopamine. Antibody blockade of MAdCAM-1 abrogated the optogenetic effect on immune cell extravasation into the colon and the pathology. Thus, sympathetic fibers control colonic inflammation by regulating immune cell extravasation from circulation, a mechanism likely relevant in multiple organs.

INTRODUCTION

As a central regulator of homeostasis, the brain receives multiple layers of information from the body, including feedback regarding metabolism, temperature, inflammation and tissue damage. The brain integrates this information to orchestrate behavioral and physiological functions, including the activity of the organism's main protective mechanism, the immune system (Ben-Shaanan et al., 2018; Elenkov et al., 2000; Sloan et al., 2007; Takahashi et al., 2018). Thus, for example, immune activity is synchronized with the circadian rhythm (Druzd et al., 2017) and with psychological states like stress, which can induce immune cell activation (Rinner et al., 1992; Steptoe et al., 2007), mobilization (Dhabhar et al., 2012; Viswanathan and Dhabhar, 2005), or immune suppression (Dhabhar and McEwen, 1997). Activity of specific brain areas, such as the brain's reward system, which is endogenously activated in anticipation of positive experiences (Schultz, 1998; Tsai et al., 2009), boosts the anti-bacterial and anti-tumor immune response (Ben-Shaanan et al., 2016; Ben-Shaanan et al., 2018). On the other hand, stroke suppresses inflammation via hepatic invariant natural killer T (iNKT) cells (Wong et al., 2011). These diverse effects of the brain on immunity can be mediated by hormonal mediators (e.g., cortisol; Morey et al., 2015), or peripheral innervations, via the parasympathetic nervous system (PSNS) and the sympathetic nervous systems (SNS). Here, we focus on the SNS as a key pathway relaying information from the brain to the immune system (Elenkov et al., 2000; Nance and Sanders, 2007).

Anatomically, the SNS is comprised of two arms, an endocrine and a local arm. In the endocrine arm, neuronal fibers that innervate the adrenal gland induce adrenaline and noradrenaline (NA) release to the bloodstream, affecting the whole organism. In contrast, the local arm is comprised of sympathetic fibers directly innervating various tissues and immune organs (Felten et al., 1985; Nance and Sanders, 2007). These sympathetic fibers can act independently of each other (Jänig, 2014), focally releasing NA. This local release forms an infrastructure that can potentially relay unique information to control immune activity at diverse sites. Local control of immunity is especially important, as immune reactions must be spatially confined to avoid an overwhelming systemic response (e.g., sepsis). However, most studies establishing the connection between the SNS and

 © 2021 The Authors. Published by Elsevier Inc.
This is an open access article under the CC BY license (http://creativecommons.org/licenses/by/4.0/).

CellPress
OPEN ACCESS

immunity use systemic pharmacological interventions or adrenalectomy, thereby manipulating the endocrine arm of the SNS (Jänig, 2014). This leaves a major gap in our understanding of the local arm of the SNS and the mechanisms whereby the brain controls the local immune response.

Many inflammatory conditions are affected by the psychological state of the patient, specifically stress (Godbout and Glaser, 2006; Liu et al., 2017), which induces SNS activation. Such a connection between the mental state and inflammation is especially evident in the context of the highly innervated and immunologically active organ, the gastrointestinal tract (GIT), as seen for example, in inflammatory bowel disease (IBD; Mawdsley and Rampton, 2005; Saunders et al., 2006; Taylor and Keely, 2007). The GIT contains an internal nervous system, the enteric nervous system (ENS), and, as part of the gut-brain axis (Bellono et al., 2017; Cryan and Dinan, 2012; Foster and McVey Neufeld, 2013; Han et al., 2018; Kaelberer et al., 2018), receives inputs from the brain via the PSNS and SNS (Altschuler et al., 1993; Gabanyi et al., 2016; Straub et al., 2006; Veiga-Fernandes and Mucida, 2016). The sympathetic fibers in the GIT innervate the mucosa (Straub et al., 2006), the gut-associated lymphoid tissue (GALT; Veiga-Fernandes and Mucida, 2016), and the local blood vessels (Sheng and Zhu, 2018). Nevertheless, the effects of the SNS on GIT inflammatory conditions are mainly analyzed using systemic approaches (Bai et al., 2009; Johnson et al., 2005; Marra et al., 2005; Zádori et al., 2016), overlooking the potential effects of local sympathetic innervation on GIT inflammation.

In this study, we used optogenetic manipulations to investigate the effects of local sympathetic innervation to the colon in a murine model of colitis, dextran sulfate sodium (DSS)-induced colitis (Eichele and Kharbanda, 2017). To locally activate the sympathetic fibers in the colon, an optogenetic probe was inserted intra-rectally to transgenic mice expressing the optogenetic channel, channelrhodopsin-2 (ChR2) in tyrosine hydroxylase (TH) expressing cells. Thus, we activated the sympathetic fibers located in the colon and induced local NA release. In contrast to systemic application of NA, the optogenetic activation of the sympathetic fibers in the colon attenuated the clinical symptoms of the DSS-induced colitis and diminished immune cell abundance in the inflamed site. This effect was mediated via a reduction in MAdCAM-1 expression on endothelial cells, an important factor for immune cell extravasation from the blood to the GIT (Berlin et al., 1993). Taken together, our study identifies a mechanism whereby the local sympathetic fibers control the circulation-tissue gateway via changes in the expression of endothelial cell adhesion molecules. These effects of local sympathetic activation are distinct from systemic NA administration, highlighting the complex effects of the SNS on inflammatory conditions (Pongratz and Straub, 2014). Finally, this study suggests a potential therapeutic mechanism for regulating local inflammatory conditions.

RESULTS

Optogenetics activate local sympathetic fibers in the colon

To characterize the effects of local sympathetic innervations on GIT immunity, we focused on the colon. We used optogenetics to specifically activate the local sympathetic fibers in this tissue. This approach was previously applied in peripheral tissues (Cohen et al., 2019; Montgomery et al., 2016; Pirzgalska et al., 2017) and enables light-dependent activation of specific neurons that express the optogenetic channel, ChR2 (Deisseroth, 2011). To express the ChR2 in sympathetic neurons, we crossbred mice expressing ChR2 and a fluorescent reporter in a *Cre*-dependent manner, with TH-*Cre* mice. TH is an enzyme required for catecholamine synthesis (Kuhar et al., 1999), thereby targeting ChR2 expression to sympathetic neurons, which allows their selective activation. The cell bodies of the sympathetic neurons are located within the sympathetic ganglia, sending axons that innervate various organs, including the colon (Figure 1A). Using immunohistochemistry, we validated the expression of ChR2, identified by a fluorescent reporter, in the sympathetic cell bodies (Figure 1A). As our goal was to use optogenetics to locally activate the sympathetic fibers to the colon, we verified that ChR2 was also expressed in the colon's sympathetic fibers. We applied a clearing technique which enabled us to visualize the structure of the colon in high resolution. The ChR2 fluorescent reporter, TH and β3-tubulin (a neuronal marker) were co-localized in the colon (Figures 1B and 1C). Thus, in our model, ChR2 was expressed in the sympathetic cell bodies (located in the sympathetic ganglia) and in TH^+ neuronal fibers in the colon, making them potentially amenable to the optogenetic manipulation.

TH is also expressed in other cell populations besides sympathetic neurons (Brumovsky et al., 2006; Daubner et al., 2011), which could potentially express ChR2 in our model. In the GIT, TH is expressed mainly in dopamine and serotonin-secreting cells (Blaugrund et al., 1996; Daubner et al., 2011; Karasawa et al., 1997; Obermayr et al., 2013; Gabanyi et al., 2016). Although we could not detect ChR2 expression in $serotonin^+$ cells (Figure S1A), ChR2 was expressed in dopaminergic enteric neurons, evident in the TH^+ enteric plexus (Figures S1B and S1C), known to contain dopaminergic neurons (Figure S1D; Natale et al., 2017). In addition to sympathetic neurons, TH is also expressed in a small fraction of non-neuronal populations, notably in some subsets of immune cells (Cosentino et al., 2007; Flierl et al., 2008; Nguyen et al., 2011). Thus, we analyzed the expression of the ChR2 fluorescent reporter in immune cells residing in the colon. In line with the low expression of TH in immune cells compared to that of neurons (Gabanyi et al., 2016), no immune cells were shown to express the ChR2 fluorescent reporter by immunohistochemistry or flow cytometry (Figures 1D, S1E, and S1F). Therefore, in our transgenic model, the sympathetic nerve fibers express ChR2 and thus, could be potentially targeted by the optogenetic manipulation.

The light sensitive ChR2 ion channel induces neuronal activation upon illumination with blue light (460nm). To achieve localized activation of the sympathetic fibers only at the colon, we inserted an optic fiber intra-rectally to anesthetized mice (Figure 1E) and activated the fibers based on previous reports (Başar, 2011; Kubota et al., 1995; Ootsuka et al., 1995). To demonstrate the functional activation of the ChR2 fibers following the optogenetic stimulation, we used calcium imaging. We loaded colons of ChR2/TH mice with an acetoxymethyl (AM) form of the red calcium fluorescent indicator Calbryte 590. This indicator penetrates the cytoplasm, and upon cell activation intracellular calcium concentration rises, increasing the

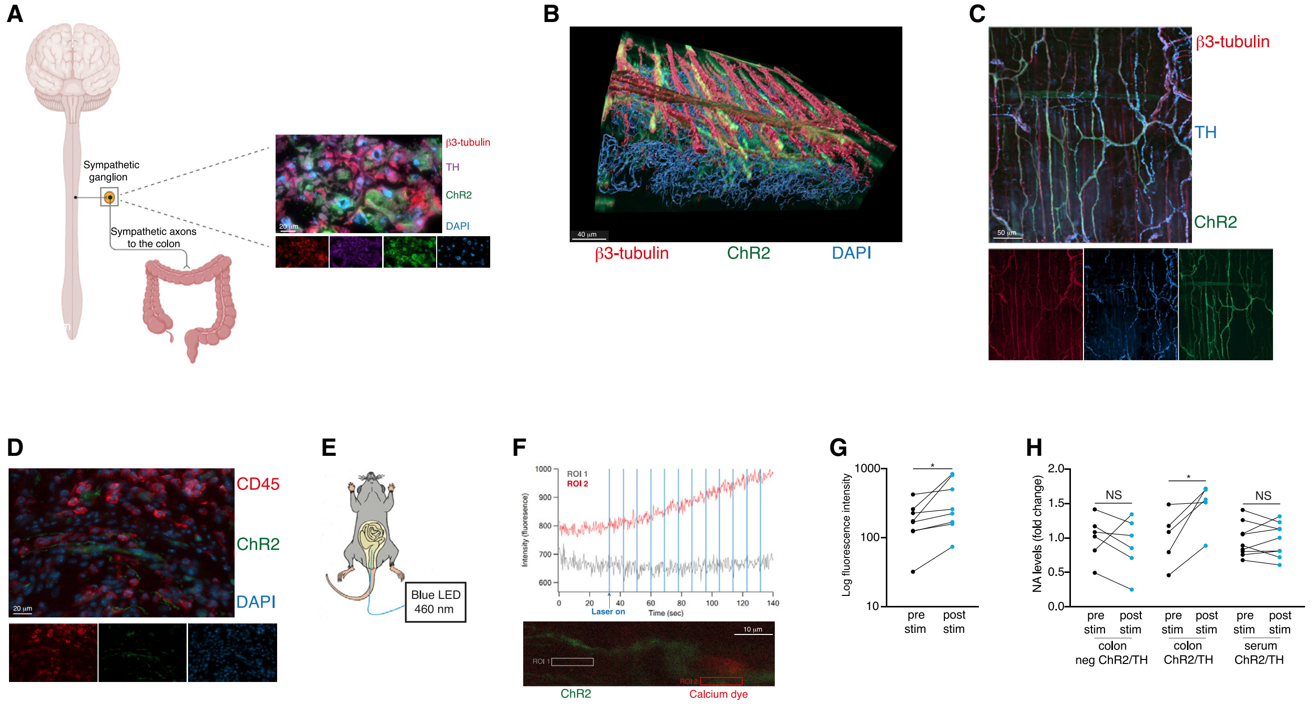

Figure 1. Optogenetics activates local sympathetic fibers in the colon

(A) Left: schematic representation of the SNS: the SNS originates from the brain, travels via the sympathetic ganglia, and sends axons innervating various organs, including the colon. Right: expression of β3-tubulin (red), TH (purple), ChR2 fluorescent marker (green), and DAPI (blue) in the sympathetic ganglion of ChR2/TH mice. Scale bar, 20 μm.

(B) Expression of β3-tubulin (red), ChR2 fluorescent marker (green), and DAPI (blue) in the colon of ChR2/TH mice that underwent tissue clearing. Scale bar, 40 μm.

(C) Expression of β3-tubulin (red), TH (blue), and ChR2 fluorescent marker (green) in the colon of ChR2/TH mice that underwent tissue clearing. Scale bar, 50 μm.

(D) Expression of CD45 (red), ChR2 fluorescent marker (green), and DAPI (blue) in the colon of ChR2/TH mice. Scale bar, 20 μm.

(E) Experimental procedure: anesthetized mice were subjected to blue light illumination using an intra-rectal optogenetic probe, inserted into the colon. Light was delivered for 30 min, comprised of 60 cycles of (10 s of 1 ms pulses at 10 Hz, followed by a 20 s break).

(F) Bottom panel: representative image of calcium dye (Calbryte 590; red) and ChR2 fluorescence marker (green). Region of interest (ROI) 1 (gray): An example of a region negative for the two fluorescent indicators. ROI 2 (red): an example of a region co-expressing both fluorescent indicators. Upper panel: representative graph showing the fluorescence intensity of ROI 1 and 2 as a function of time before and during optogenetic stimulation (blue horizontal lines represent the timing of the blue light illumination). Scale bar, 10 μm.

(G) Fluorescence intensity of red calcium dye (Calbryte 590) in regions co-expressing the green ChR2 fluorescent marker and the red calcium dye, before (pre stim) and after (post stim) optogenetic stimulation. N = 8, ROIs from three different ChR2/TH mice.

(H) Fold change of NA levels measured in the colon or serum of ChR2/TH mice and transgene negative littermates (who do not express ChR2) before (pre stim), and after (post stim) optogenetic activation. Negative littermate's colon: N = 6; ChR2/TH colon: N = 5; ChR2/TH serum: N = 9. Mean ± SEM and individual mice are presented for each group. Student's paired t test. * = $p < 0.05$. Data represent at least two independent repeats.

indicator's red fluorescence intensity. The location of ChR2 fibers was identified by green fluorescence (the ChR2 fluorescent reporter), while activity was identified by the increase in the calcium indicator's red fluorescence intensity. In fibers that co-expressed the red calcium dye with the green ChR2 indicator, we observed a significant increase in the red fluorescence intensity following the optogenetic stimulation ($p = 0.0403$; Figures 1F and 1G). These results demonstrate that the ChR2 fibers in the colon are functionally activated by the optogenetic stimulation. Moreover, the local optogenetic activation in the colon via the intra-rectal probe specifically affected the sympathetic fibers, as we observed a significant increase in NA levels in the colons of ChR2/TH mice following the optogenetic activation (colon ChR2/TH: $p = 0.0288$; colon negative littermates: $p = 0.4798$; Figures 1H, S1G, and S1H). These were local effects, as there were no changes in serum NA levels in the ChR2/TH mice following the optogenetic activation (serum ChR2/TH: $p = 0.9791$; Figures 1H and S1I). Thus, these results support the capacity of the optogenetic manipulation to locally activate the sympathetic fibers in the colon.

Local sympathetic activation attenuates clinical symptoms of DSS-induced colitis

Having established the functionality of the optogenetic system, we next tested the effects of local sympathetic activation in a mouse model of colon inflammation, DSS-induced colitis (Eichele and Kharbanda, 2017). In this model, the addition of DSS to the drinking water disrupts the integrity of the epithelial layer, leading to colon inflammation. This results in gradual weight loss and shortening of the colon (Chassaing et al., 2014). Although this is a widely used model for colitis, it is important to note that the induced inflammation is a form of

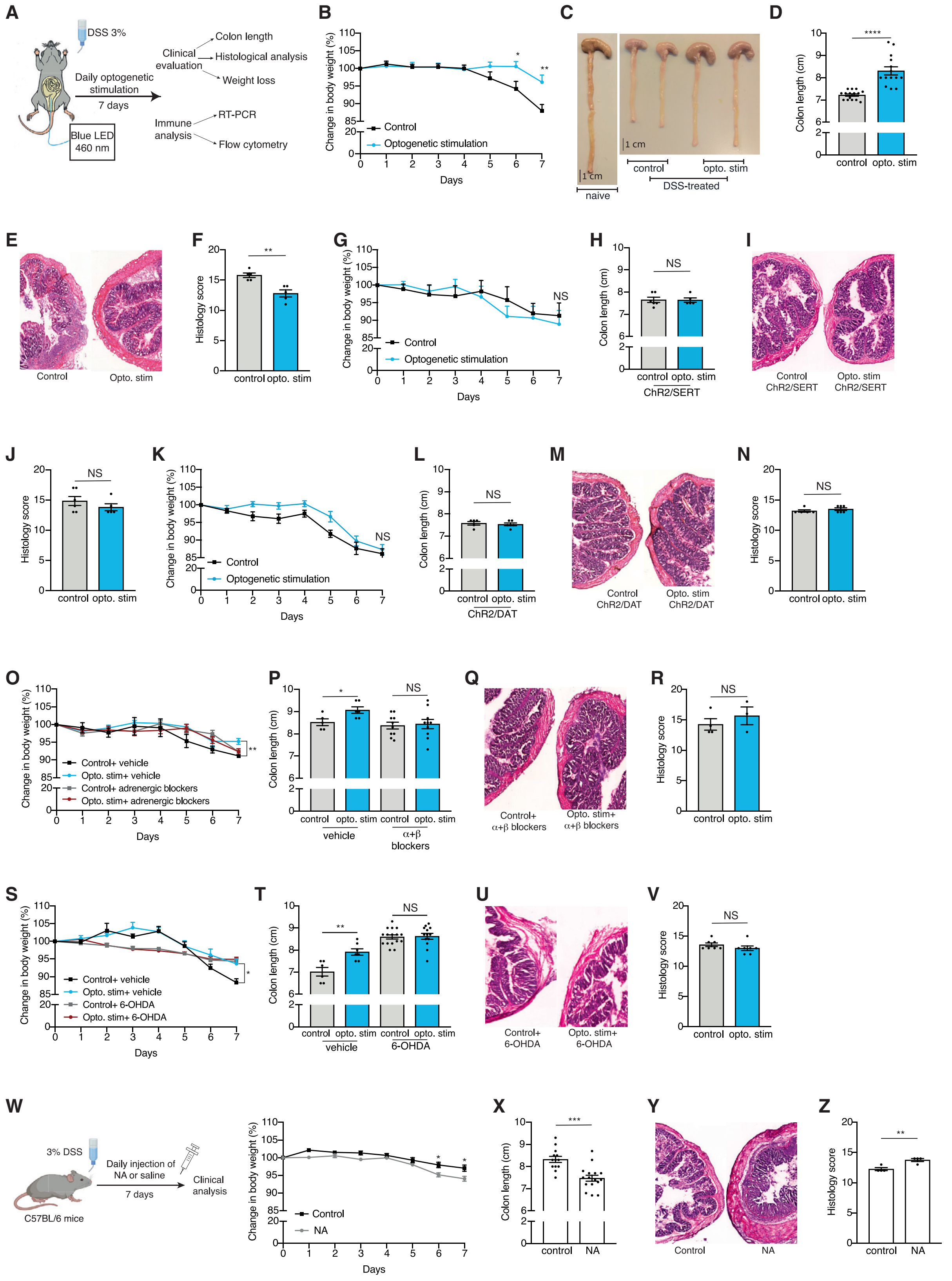

(legend on next page)

chemical colitis, and thus differs from the endogenous inflammation process.

In our experiments, we used two groups of mice: ChR2/TH mice as the experimental group, and negative littermates lacking ChR2 expression undergoing the same experimental procedure served as the control. Both groups were treated with 3% DSS in their drinking water and underwent local optogenetic activation to their colons via an intra-rectal probe daily for 7 days (Figure 2A). To determine whether the optogenetic activation of the sympathetic fibers affected the clinical manifestations of the colitis, we weighed the mice daily, and measured the length of their colons following sacrifice at the end of the experiment. The optogenetic activation did not affect food or water consumption (Figures S2A and S2B), or locomotion (Figure S2C). Nevertheless, the ChR2/TH mice exhibited significantly less weight loss (day 6: $p = 0.034$; day 7: $p = 0.0084$; Figure 2B) and reduced colon shortening ($p < 0.0001$; Figures 2C and 2D), which is not due to a baseline difference in the colon lengths between the two groups (Figure S2D). Moreover, there was a significant difference in the colitis histological severity score between the two groups, indicating less severe disease in the ChR2/TH mice, manifested by inflammatory infiltrates, goblet cell loss, crypt density, crypt hyperplasia, muscle thickening, submucosal inflammation, crypt abscess, and ulceration ($p = 0.0025$; Figures 2E and 2F; Koelink et al., 2018). The effects of the optogenetic activation on weight loss and colon length were evident only in the presence of colon inflammation and were not apparent in naive mice, which underwent daily optogenetic activation without exposure to DSS (Figures S2E and S2F). Thus, local optogenetic activation of the sympathetic fibers in the colon significantly attenuated the clinical symptoms of DSS-induced colitis.

Nevertheless, as previously noted, neurons in the enteric plexus express the ChR2 channel (Figures S1B and S1C), known to contain dopaminergic neurons (Figure S1D; Natale et al., 2017). Moreover, although we did not observe ChR2 expression in serotonin^{+} cells (Figure S1A), based on previous reports, TH may be also expressed by serotonergic cells, and thus, can potentially express ChR2 (Blaugrund et al., 1996; Karasawa et al., 1997; Obermayr et al., 2013). Therefore, the effects of optogenetic activation could potentially be mediated by these cell populations rather than by manipulation of the SNS. To control for this possibility, we generated two new transgenic mouse strains, ChR2/dopamine transporter (DAT) and ChR2/serotonin transporter (SERT), expressing the ChR2 in dopamine or serotonin-producing cells, respectively. We validated the ChR2 expression in DAT^{+} and SERT^{+} cells in the colons of ChR2/DAT and ChR2/SERT mice, respectively (Figures S2G and S2H). We then repeated our experimental protocol applying daily

Figure 2. Local sympathetic activation attenuates clinical symptoms of DSS-induced colitis

(A) Experimental design: ChR2/TH mice and their controls (transgene negative littermates exposed to light stimulation) underwent daily optogenetic activation of their colon (as described in Figure 1E) and were supplemented with 3% DSS for 7 days.

(B) Percentage change in weight during the 7 days of 3% DSS administration and daily optogenetic activation of ChR2/TH mice and their controls. N = 13, 10.

(C) Representative image of colons obtained from ChR2/TH mice and their controls following 7 days of 3% DSS and daily optogenetic activation (left is a naive colon that was not exposed to DSS). Scale bar, 1 cm.

(D) Colon length of ChR2/TH mice and their controls following 7 days of 3% DSS and daily optogenetic activation. N = 15, 14.

(E and F) Representative histological image (E) and histological severity score (F) of colons obtained from ChR2/TH mice and their controls following 7 days of 3% DSS and daily optogenetic activation. N = 5, 5.

(G) Percentage change in weight during 7 days of 3% DSS administration and daily optogenetic activation of ChR2/SERT mice and their controls (transgene negative littermates exposed to light stimulation). N = 6, 4.

(H) Colon length of ChR2/SERT mice and their controls following 7 days of 3% DSS and daily optogenetic activation. N = 6, 5.

(I and J) Representative histological image (I) and histological severity score (J) of colons obtained from ChR2/SERT mice and their controls following 7 days of 3% DSS and daily optogenetic activation. N = 6, 5.

(K) Percentage change in weight during 7 days of 3% DSS administration and daily optogenetic activation of ChR2/DAT mice and their controls (ChR2/DAT mice that underwent the same procedure but were not exposed to the light stimulation). N = 5, 6.

(L) Colon length of ChR2/DAT mice and their controls following 7 days of 3% DSS and daily optogenetic activation. N = 5, 6.

(M and N) Representative histological image (M) and histological severity score (N) of colons obtained from ChR2/DAT mice and their controls following 7 days of 3% DSS and daily optogenetic activation. N = 5, 6.

(O) Percentage change in weight during 7 days of 3% DSS administration and daily optogenetic activation of ChR2/TH mice and their controls. 15 min before each optogenetic activation, the mice received IP injections of the adrenergic blockers Phentolamine (10 mg/kg) and Nadolol (5 mg/kg), or PBS (vehicle group). Vehicle: N = 5, 5; adrenergic blocker: N = 10, 10.

(P) Colon length of ChR2/TH mice and their controls following 7 days of 3% DSS, daily optogenetic activation and injection of adrenergic blockers or vehicle (as described in Figure 2O). Vehicle: N = 5, 6; adrenergic blocker: N = 10, 10.

(Q and R) Representative histological image (Q) and histological severity score (R) of colons obtained from ChR2/TH mice and their controls following 7 days of 3% DSS, daily optogenetic activation and injection of adrenergic blockers (as described in Figure 2O). N = 4, 3.

(S) Percentage change in weight during 7 days of 3% DSS and daily optogenetic activation of ChR2/TH mice and their controls injected 5 days before with 6-OHDA or vehicle. Vehicle: N = 6, 6; 6-OHDA: N = 16, 13.

(T) Colon length of ChR2/TH mice and their controls following 6-OHDA or vehicle injections and 7 days of 3% DSS administration and daily optogenetic activation. Vehicle: N = 6, 7; 6-OHDA: N = 16, 13.

(U and V) Representative histological image (U) and histological severity score (V) of colons obtained from ChR2/TH mice and their controls following 6-OHDA injections, 7 days of 3% DSS administration and daily optogenetic activation. N = 7, 7.

(W) Left: experimental design: C57BL/6 mice were supplemented with 3% DSS and received daily IP injections of NA (5 mg/kg) or saline (control group). Right: percentage change in weight during 7 days of 3% DSS administration and daily NA or saline injection. N = 16, 18.

(X) Colon length of the NA-treated mice and their controls following 7 days of 3% DSS. N = 13, 16.

(Y and Z) Representative histological image (Y) and histological severity score (Z) of colons obtained from NA-treated mice and their controls following 7 days of 3% DSS. N = 4, 4. Mean ± SEM, as well as results for individual mice, are presented for each group; Student's unpaired t test. * = $p < 0.05$, ** = $p < 0.01$, *** = $p < 0.001$, **** = $p < 0.0001$. Data represent at least two independent repeats.

CellPress
OPEN ACCESS

optogenetic stimulation to the colons of DSS-treated mice for 7 days. In contrast to the reduction in the clinical symptoms observed in the ChR2/TH mice (Figures 2B–2F), the optogenetic activation had no effect on any of the clinical parameters in the ChR2/SERT or ChR2/DAT mice (ChR2/SERT: p = 0.3485 weight, p = 0.9507 colon length, p = 0.3197 histological score; ChR2/DAT: p = 0.5617 weight, p = 0.6491 colon length, p = 0.3526 histological score; Figures 2G–2N). Thus, we conclude that although ChR2 is expressed in some dopaminergic neurons in the colon, optogenetic activation of the dopaminergic or serotonergic populations cannot account for the observed effects in the ChR2/TH mice.

To further validate that the effects of the optogenetic activation were mediated by the SNS and its main neurotransmitter NA, we used adrenergic blockers (Phentolamine, an α adrenergic blocker, and Nadolol a β adrenergic blocker). We injected the adrenergic blockers daily to the ChR2/TH mice and their littermate controls, undergoing daily optogenetic activation to their colons during DSS treatment for 7 days. In the presence of the adrenergic blockers, the optogenetic activation did not affect the clinical symptoms of the colitis, further indicating that the optogenetic effect is NA-dependent (weight: vehicle p = 0.0051 and blockers p = 0.9915; colon shortening: vehicle p = 0.0308 and blockers p = 0.7884; histological score p = 0.43; Figures 2O–2R). As additional validation of the necessity of the SNS for the observed effects on the colitis clinical manifestations, we injected ChR2/TH mice and their littermate controls with 6-hydroxydopamine (6-OHDA). When 6-OHDA is injected to the periphery, it does not cross the blood-brain barrier (BBB; Kostrzewa and Jacobowitz, 1974). Thus, 6-OHDA specifically denervates peripheral catecholaminergic neurons, including in the colon as evident by an 86.21 ± 16.42% reduction in colon NA following 6-OHDA injection (p = 0.0005; Figure S2I). We repeated our experimental paradigm of daily optogenetic activation to the colon during DSS treatment for 7 days in the denervated ChR2/TH mice and their controls. In line with the pharmacological adrenergic blockers results, the optogenetic activation did not mitigate the clinical symptoms of the colitis in the 6-OHDA-treated ChR2/TH mice (weight: vehicle p = 0.0103 and 6-OHDA p = 0.5941; colon shortening: vehicle p = 0.0030 and 6-OHDA p = 0.8538; histological score p = 0.2577; Figures 2S–2V). Although the adrenergic blockers and the 6-OHDA are not limited to the colon, these results further indicate that the effects following the optogenetic manipulation are dependent on sympathetic activity, and specifically on NA.

The beneficial effects of the local sympathetic activation on DSS-induced colitis were in contrast to previous reports showing that stress and systemic-pharmacological adrenergic manipulations are associated with exacerbation of IBD (Bai et al., 2009; Gracie et al., 2019; Hart and Kamm, 2002; Johnson et al., 2005; Marra et al., 2005; Mawdsley and Rampton, 2005; Zádori et al., 2016). This apparent discrepancy suggested that systemic versus local release of NA may have distinct effects on colitis. To test this possibility, we intraperitoneally (IP) injected DSS-treated mice with NA, or saline as a control (Figure 2W). In line with previous reports (Gracie et al., 2019; Hart and Kamm, 2002; Mawdsley and Rampton, 2005; Zádori et al., 2016), the systemic injection of NA resulted in worsening of the colitis, manifested by increased weight loss (day 6: p = 0.0106; day 7: p = 0.0225; Figure 2W), enhanced shortening of the colon (p = 0.0001; Figure 2X) and a more severe histological score (p = 0.0054; Figures 2Y and 2Z). Thus, while a systemic increase in NA levels exacerbates the disease, the local increase in NA attenuates it, suggesting differential effects of the local and endocrine arms of the SNS.

Local sympathetic activation reduces inflammation and immune cell abundance in the colon of DSS treated mice

The colon contains two main immune layers, the intraepithelial lymphocyte (IEL) layer and the lamina propria (LP) layer. Immune cells in the IEL are interspersed between epithelial cells and are the first cells to encounter luminal antigens (Cheroutre et al., 2011). The LP is a layer of connective tissue residing beneath the epithelial layer, rich in immune cells and blood vessels (Coombes and Powrie, 2008; Fagarasan and Honjo, 2003; Hadis et al., 2011; Varol et al., 2009; Varol et al., 2010). To determine whether the local optogenetic activation affected the inflammatory response in the colon, we repeated our experimental paradigm (Figure 2A), and analyzed the tissue expression of cytokines, and the immune cells in the LP and IEL. Following the optogenetic activation, there was a significant decrease in the mRNA levels of the cytokines IL-6, TNF-α, IL-12, IL-21, IL-1β, and IL-17 (Figure 3A), and a non-significant trend was evident in IFN-γ, IL-10 and TGF-β (Figure 3A) in the colons of the DSS-treated ChR2/TH mice. Moreover, following the optogenetic activation, immune cell abundance (number of $CD45^+$ cells/gr colon) was also significantly attenuated in both the LP and the IEL in the DSS-treated ChR2/TH mice (p = 0.0297 and p < 0.0001 respectively; Figures 3B and S2J–S2L). The diminished abundance of immune cells following optogenetic activation was dependent on sympathetic activity as it was abolished when the DSS-treated ChR2/TH mice were injected with adrenergic blockers (Phentolamine and nadolol; LP: p = 0.1591 and IEL: p = 0.5147; Figure 3C) or treated with 6-OHDA (LP: p = 0.2759 and IEL: p = 0.1439; Figure 3D).

Our analysis of the various immune subpopulations in the colon did not identify a specific cell subset that could account for the reduction in immune cell abundance following the optogenetic activation (Figures 3E, 3F, and S3). Moreover, we could not identify significant changes in the expression of functional markers on any of the examined immune subpopulations (Figure S4). Although the colon in naive mice (i.e., without exposure to DSS) is highly populated by immune cells, daily optogenetic activation of naive ChR2/TH mice and their littermate controls did not affect immune abundance (Figure S5A), suggesting that the optogenetic effect may be dependent on inflammatory factors. Moreover, the decrease in the pro-inflammatory cytokine levels in the colon following the optogenetic activation (Figure 3A) suggests that an inflammation-linked change in vascular permeability is not likely to be responsible for the reduction in the immune population within the LP and IEL (Figure 3B).

Local sympathetic activation attenuates endothelial MAdCAM-1 levels

The observed decrease in immune cell abundance could be mediated by either change in cell proliferation, cell death, or migration. The optogenetic activation did not affect the number of dead or proliferating immune cells in the ChR2/TH colons

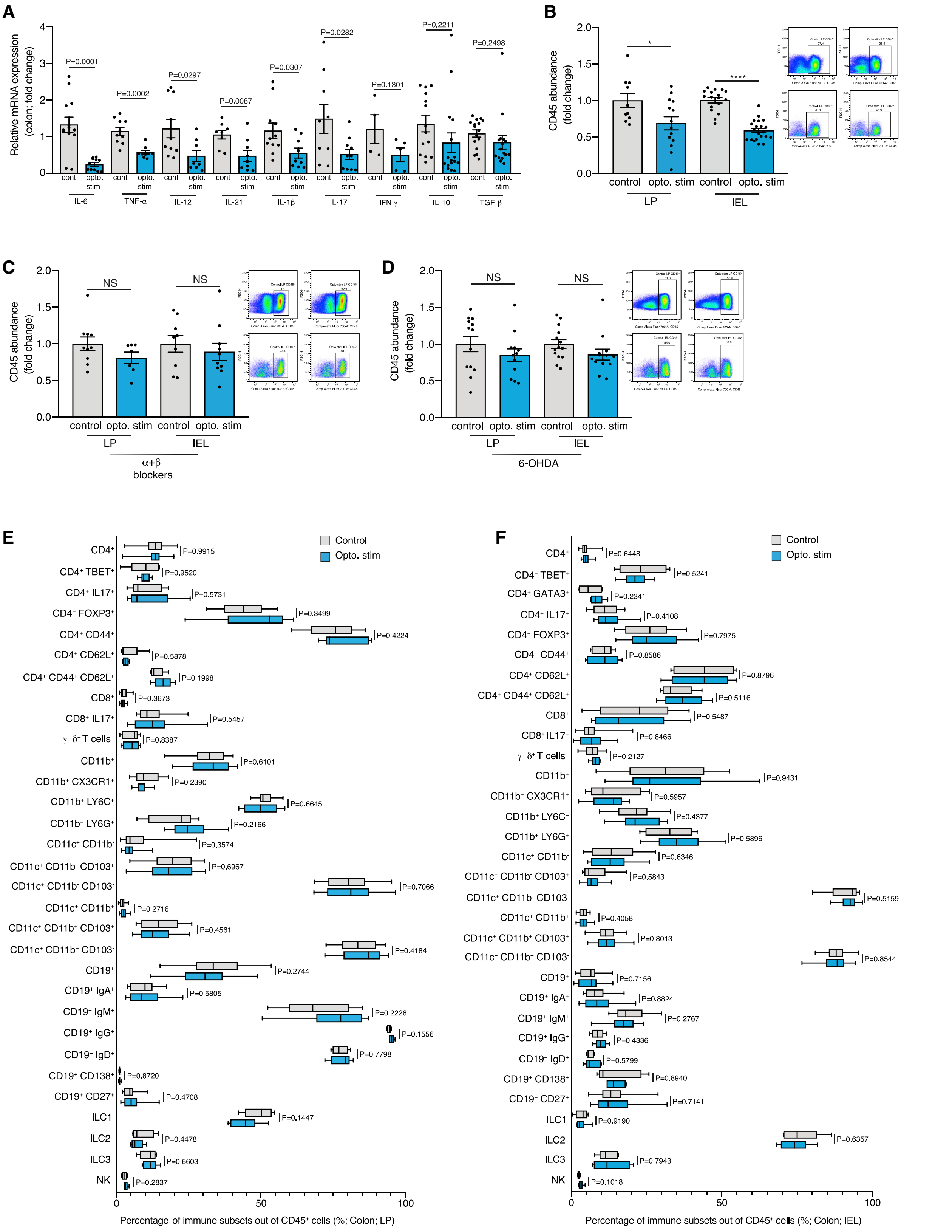

(legend on next page)

CellPress
OPEN ACCESS

(tested by staining for TUNEL and Ki67; Figures S5B and S5C). Thus, we focused on the potential effects of our manipulation on cell migration, especially since sympathetic activity correlates with alterations in immune cell trafficking (Dantzer, 2018; Straub et al., 2006; Suzuki et al., 2016). We analyzed changes in the mRNA levels of several migration-related factors in the colon: the intestine homing receptor, CCR9 (Habtezion et al., 2016); the lymph node-homing receptor, CCR7 (von Andrian and Mempel, 2003); and the gut-homing α4β7 integrin (Meenan et al., 1997). These molecules are expressed on immune cells and guide immune cell trafficking. Additionally, we tested motility-guiding molecules expressed by the colon microenvironment (chemokines: CCL20, CCL25 and selectins: E-selectin, and P-selectin). However, the expression of these factors was not significantly altered by the optogenetic activation in the DSS-treated ChR2/TH mice (Figure 4A).

We therefore considered the possibility that the effects of the optogenetic activation may be evident only at specific sites with high sympathetic activity, and that these effects were masked when we analyzed the entire colon tissue. To identify potential sites of interaction between sympathetic fibers and immune cells, we performed an anatomical characterization of the colon. We noticed that many of the ChR2-expressing TH^+ fibers in the colon were localized around blood vessels (Figures 4B and 4C**)**. This is in line with previous reports showing that sympathetic fibers envelop blood vessels (Anlauf et al., 2003; Guyenet, 2006; Sheng and Zhu, 2018; Wehrwein et al., 2016), and may alter migration-related molecules of the microvasculature (Scheiermann et al., 2012). This observation suggested that the optogenetic activation could potentially affect endothelial cells, which line the interior surface of blood vessels. Given that endothelial cells serve as a barrier modulating immune cell extravasation from the circulation to inflammatory sites, alterations in these cells could account for the observed change in immune abundance (Figure 3B).

Endothelial cells express various cell adhesion molecules, including VCAM-1, ICAM-1 and MAdCAM-1 (Lawson and Wolf, 2009; Ogawa et al., 2005) that regulate immune cell extravasation to the local tissue (Mackay and Imhof, 1993; Springer, 1990). mRNA and flow cytometry analyses revealed that following optogenetic activation, there was a significant reduction in the expression of the cell adhesion molecule MAdCAM-1 (the ligand for α4β7; Berlin et al., 1993), and to a lesser extent, of ICAM-1 in the DSS-treated ChR2/TH mice (mRNA: VCAM-1 $p = 0.1905$, MAdCAM-1 $p = 0.0026$ and ICAM-1 $p = 0.0081$. Flow cytometry: MAdCAM-1 $p = 0.0005$ and ICAM-1 $p = 0.0182$; Figures 4D, 4E, and S5D–S5F). MAdCAM-1 and ICAM-1 regulate the migration of many types of leukocytes (Berlin et al., 1993; Lawson and Wolf, 2009), which may account for the observed uniform reduction of the immune populations following the optogenetic activation (Figures 3B, 3E, and 3F). The change in MAdCAM-1 and ICAM-1 levels was a local effect, as their levels were not altered even in the adjacent small intestine (MAdCAM-1 $p = 0.9550$ and ICAM-1 $p = 0.8846$; Figure 4F), further highlighting the local nature of the optogenetic activation. The levels of MAdCAM-1 and ICAM-1 on endothelial cells were also reduced, though not significantly, in naive ChR2/TH mice that were optogenetically stimulated but not exposed to DSS (Figures S5G and S5H). This is in line with previous reports (Ando et al., 2007; Briskin et al., 1997) and with our analysis showing an increase in ICAM-1 and MAdCAM-1 levels on endothelial cells throughout DSS-induced colitis (Figures S5I and S5J). Moreover, this non-significant reduction in MAdCAM-1 and ICAM-1 levels in non-inflamed mice, can account for the limited effect of the optogenetic activation on immune cell abundance in naive mice (Figure S5A).

To confirm that the reduction in MAdCAM-1 and ICAM-1 levels on endothelial cells following optogenetic activation was dependent on sympathetic activity, we applied adrenergic blockers (Phentolamine and Nadolol) or 6-OHDA in the DSS-treated ChR2/TH mice and their controls undergoing daily optogenetic activation. We could not detect any change in MAdCAM-1 and ICAM-1 expression levels on endothelial cells under these conditions (adrenergic blockers: MAdCAM-1 $p = 0.7815$ and ICAM-1 $p = 0.8634$; 6-OHDA: MAdCAM-1 $p = 0.1937$ and ICAM-1 $p = 0.2349$; Figures 4G and 4H). Since the effect on MAdCAM-1 was more prominent than on ICAM-1, and as blocking MAdCAM-1 was previously shown to attenuate IBD (Vermeire et al., 2017), we focused on MAdCAM-1.

To further establish the direct effect of NA on endothelial MAdCAM-1 levels, we performed *ex vivo* experiments in which we

Figure 3. Local sympathetic activation reduces inflammation and immune cell abundance in the colon of DSS treated mice
ChR2/TH mice and their controls (transgene negative littermates exposed to light stimulation) underwent daily optogenetic activation of their colon (as described in Figure 1E) and were supplemented with 3% DSS for 7 days.
(A) Relative mRNA expression levels of IL-6, TNF-α, IL-12, IL-21, IL-1β, IL-17, IFN-γ, IL-10 and TGF-β in the colon tissue of ChR2/TH mice and their controls following 7 days of 3% DSS and daily optogenetic activation. Fold change between the ChR2/TH mice and their controls relative to the mean of the control group.
(B) Left: fold change in abundance of total immune cells (number of $CD45^+$ cells/gr colon) in the LP and IEL layers of the colon from ChR2/TH mice and their controls following 7 days of 3% DSS and daily optogenetic activation. Fold change between the ChR2/TH mice and their controls relative to the mean of the control group. LP: N = 10, 14; IEL: N = 17, 21. Right: Representative flow cytometry plot demonstrating the percentage of $CD45^+$ population in the LP and in the IEL.
(C) Left: Fold change in abundance of immune cells (number of $CD45^+$ cells/gr colon) in the LP and IEL layers of the colon from ChR2/TH mice and their controls. The mice received 7 days of 3% DSS and daily IP injections of adrenergic blockers (Phentolamine 10 mg/kg and Nadolol 5 mg/kg) 15 min before each daily optogenetic activation. Fold change between the ChR2/TH mice and their controls relative to the mean of the control group. LP: N = 10, 7; IEL: N = 9, 10. Right: representative flow cytometry plot demonstrating the percentage of $CD45^+$ population in the LP and in the IEL.
(D) Left: fold change in abundance of total immune cells (number of $CD45^+$ cells/gr colon) in the LP and IEL layers of the colon from ChR2/TH mice and their controls. The mice were injected IP with 6-OHDA, and 5 days afterward received 7 days of 3% DSS and daily optogenetic activation. Fold change between the ChR2/TH mice and their controls relative to the mean of the control group. LP: N = 13, 12; IEL: N = 14, 13. Right: representative flow cytometry plot demonstrating the percentage of $CD45^+$ population in the LP and in the IEL.
(E and F) Distribution of the different immune subsets out of the $CD45^+$ population in the LP (E), and IEL layers (F) in the colons of ChR2/TH mice and their controls following 7 days of 3% DSS and daily optogenetic activation. The populations were gated from $CD45^+$ cells. Mean ± SEM, as well as data from individual mice, are presented for each group. Student's unpaired t test. * = $p < 0.05$, **** = $p < 0.0001$. Data represent at least two independent repeats.

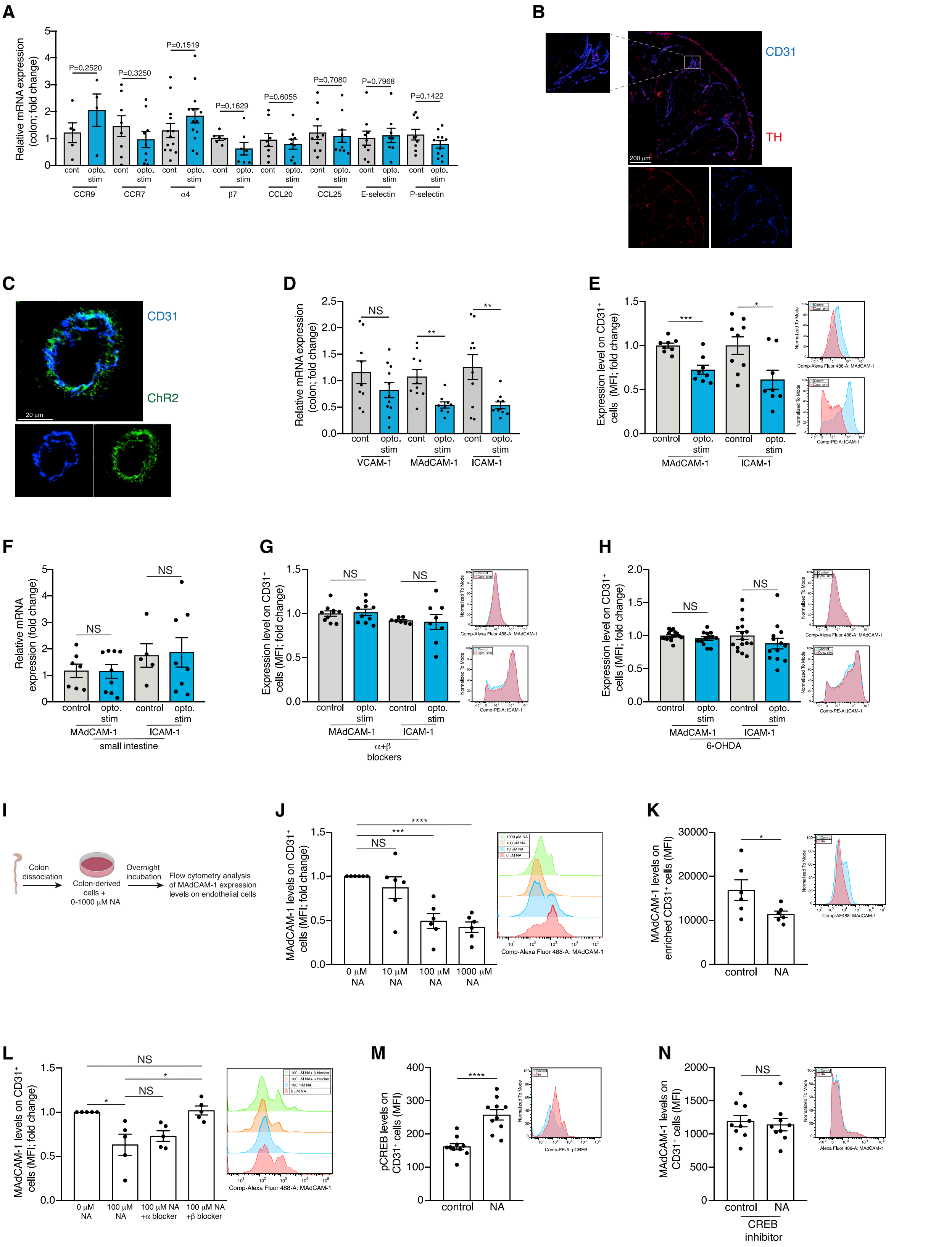

(legend on next page)

CellPress
OPEN ACCESS

enzymatically dissociated cells from colons of mice and exposed them to increasing concentrations of NA. We then analyzed by flow cytometry the expression levels of MAdCAM-1 on endothelial cells (Figure 4I). Exposure to NA significantly reduced MAdCAM-1 levels on endothelial cells (10 μM $p = 0.3221$, 100 μM $p = 0.0001$ and 1000 μM $p < 0.0001$; Figure 4J). The effect was dose-dependent and required high concentrations of NA. This may highlight the unique potential of the local sympathetic innervations to induce high local levels of NA at concentrations that cannot be tolerated at the systemic level. We further validated that the reduction in MAdCAM-1 levels was a direct effect of NA on the endothelial cells (rather than mediated via an additional cell type present in the culture). To this end, we used enriched colon-derived endothelial cells and showed that NA exposure resulted in a reduction in MAdCAM-1 levels on endothelial cells ($p = 0.0234$; Figure 4K).

To probe the mechanism and to determine which adrenergic receptor mediated these effects on MAdCAM-1 levels, we exposed the colon-derived cells to NA in the presence of an α or β adrenergic blocker and analyzed the endothelial MAdCAM-1 levels using flow cytometry. While the α adrenergic blocker did not affect MAdCAM-1 levels ($p = 0.4847$ for the change between 100 μM NA and 100 μM NA+α blocker; Figure 4L), the addition of the β adrenergic blocker completely abrogated the effects of NA on MAdCAM-1 levels ($p = 0.0175$ for the change between 100 μM NA and 100 μM NA+β blocker; $p = 0.7106$ for the change between 0 NA and 100 μM NA+β blocker; Figure 4L). Next, we characterized the intracellular signaling pathway responsible for the observed reduction of MAdCAM-1 expression on endothelial cells in the presence of NA. Previous studies demonstrated that the phosphorylation of cAMP response element-binding protein (pCREB) is an important intracellular mediator in the cellular pathway of adrenergic receptors (Lorton and Bellinger, 2015). Thus, we treated the extracted colon cells with NA and demonstrated an increased pCREB levels in endothelial cells following exposure to NA ($p < 0.0001$; Figure 4M). Moreover, exposure to a CREB inhibitor (666-15; Xie et al., 2015) eliminated the effects of NA on endothelial MAdCAM-1 levels ($p = 0.6373$; Figure 4N). Thus, we conclude that NA activates the CREB signaling pathway via the β adrenergic receptor and induces the decrease in MAdCAM-1 levels on endothelial cells.

Figure 4. Local sympathetic activation attenuates endothelial MAdCAM-1 levels

ChR2/TH mice and their controls (transgene negative littermates exposed to light stimulation) underwent daily optogenetic activation of their colon (as described in Figure 1E) and were supplemented with 3% DSS for 7 days.

(A) Relative mRNA expression levels of CCR9, CCR7, α4, β7, CCL20, CCL25, E-selectin, and P-selectin in the colon tissue of ChR2/TH mice and their controls following 7 days of 3% DSS and daily optogenetic activation. Fold change between the ChR2/TH mice and their controls relative to the mean of the control group.

(B) Expression of the markers CD31 (blue) and TH (red) in the colon of C57BL/6 mice. Scale bar, 200 μm.

(C) Expression of the markers CD31 (blue) and the ChR2 fluorescent marker (green) in the colon of ChR2/TH mice. Scale bar, 20 μm.

(D) Relative mRNA expression levels of VCAM-1, MAdCAM-1 and ICAM-1 in the colon tissue of ChR2/TH mice and their controls following 7 days of 3% DSS and daily optogenetic activation. Fold change between the ChR2/TH mice and their controls relative to the mean of the control group. VCAM-1: N = 9, 11; MAdCAM-1: N = 10, 9; ICAM-1: N = 10, 10.

(E) Left: flow cytometry analysis of MAdCAM-1 and ICAM-1 levels on endothelial cells ($CD31^+$; indicated by the fold change in MFI) in the colons of ChR2/TH mice and their controls following 7 days of 3% DSS and daily optogenetic activation. Fold change between the ChR2/TH mice and their controls relative to the mean of the control group. MAdCAM-1: N = 8, 8; ICAM-1: N = 9, 8. Right: representative flow cytometry histogram demonstrating the MAdCAM-1 and ICAM-1 levels on $CD31^+$ cells.

(F) Relative mRNA expression levels of MAdCAM-1 and ICAM-1 in the small intestine tissue of ChR2/TH mice and their controls following 7 days of 3% DSS and daily optogenetic activation. Fold change between the ChR2/TH mice and their controls relative to the mean of the control group. MAdCAM-1: N = 7, 9; ICAM-1: N = 5, 8.

(G) Left: flow cytometry analysis of MAdCAM-1 and ICAM-1 levels on endothelial cells ($CD31^+$; indicated by the fold change in MFI) in the colons of ChR2/TH mice and their controls following 7 days of 3% DSS and daily optogenetic activation. The mice received daily IP injections of adrenergic blockers 15 min before each optogenetic stimulation (Phentolamine 10 mg/kg and Nadolol 5 mg/kg). Fold change between the ChR2/TH mice and their controls relative to the mean of the control group. MAdCAM-1: N = 10, 10; ICAM-1: N = 7, 8. Right: representative flow cytometry histogram demonstrating MAdCAM-1 and ICAM-1 levels on $CD31^+$ cells.

(H) Left: flow cytometry analysis of MAdCAM-1 and ICAM-1 levels on endothelial cells ($CD31^+$; indicated by the fold change in MFI) in the colons of ChR2/TH mice and their controls. The mice were injected IP with 6-OHDA, and 5 days afterward received 7 days of 3% DSS and daily optogenetic activation. Fold change between the ChR2/TH mice and their controls relative to the mean of the control group. MAdCAM-1: N = 16, 13; ICAM-1: N = 16, 13. Right: representative flow cytometry histogram demonstrating MAdCAM-1 and ICAM-1 levels on $CD31^+$ cells.

(I) Experimental design: cells were enzymatically dissociated from colons and incubated for one hour with LPS at 37°C. Afterward, NA was added (0-1000 μM) and the cells were incubated overnight at 37°C. Then MAdCAM-1 levels were analyzed on endothelial cells ($CD31^+$) by flow cytometry.

(J) Left: MAdCAM-1 levels on endothelial cells ($CD31^+$; indicated by the fold change in MFI) following exposure to increasing concentrations of NA (as described in Figure 4I). N = 6. Right: representative flow cytometry histogram demonstrating MAdCAM-1 levels on $CD31^+$ cells.

(K) Left: MAdCAM-1 levels on colon-derived enriched endothelial cells ($CD31^+$) following exposure to 100 μM NA overnight at 37°C. N = 6. Right: Representative flow cytometry histogram demonstrating MAdCAM-1 levels on $CD31^+$ cells.

(L) Left: MAdCAM-1 levels on endothelial cells ($CD31^+$; indicated by the fold change in MFI) that were incubated at 37°C with α- or β-adrenergic blockers (Phentolamine or Nadolol respectively) followed by exposure to 100 μM NA overnight. N = 5. Right: representative flow cytometry histogram demonstrating MAdCAM-1 levels on $CD31^+$ cells.

(M) Left: pCREB levels on endothelial cells ($CD31^+$) following exposure to 100 μM NA for 30 min at 37°C. N = 10. Right: representative flow cytometry histogram demonstrating pCREB levels on $CD31^+$ cells.

(N) Left: MAdCAM-1 levels on endothelial cells ($CD31^+$) following exposure to 100 μM NA overnight at 37°C. Prior to the administration of NA, the cells were incubated with a CREB inhibitor (666-15). N = 9. Right: representative flow cytometry histogram demonstrating MAdCAM-1 levels on $CD31^+$ cells. Mean ± SEM, as well as data from individual mice, are presented for each group. Students paired t- test for figures 4K, 4M and 4N and Student's unpaired t test for the rest of the figures. * = $p < 0.05$, ** = $p < 0.01$, *** = $p < 0.001$, **** = $p < 0.0001$. Data represent at least two independent repeats.

MAdCAM-1 is necessary to mediate the beneficial effects of local sympathetic activation on DSS-induced colitis

Finally, we sought to establish, at the functional level, that the effects of the optogenetic activation on the DSS-induced colitis are MAdCAM-1 dependent. It is possible, for example, that in addition to the effect of MAdCAM-1 levels on endothelial cells, the SNS directly affected immune cells, which express the adrenergic receptors (Sanders and Straub, 2002; Scanzano and Cosentino, 2015). Moreover, the optogenetic activation could affect other aspects of GIT activity which can account for the observed effects, for example, intestinal motility (Browning and Travagli, 2014). To directly address these concerns, we injected ChR2/TH mice and their littermate controls daily with a MAdCAM-1 blocking antibody (shown to block the interaction of MAdCAM-1 with its receptor; Pullen et al., 2009) or an isotype-matched control antibody during DSS induction and daily optogenetic activation (Figure 5A). Neutralizing MAdCAM-1 abrogated the effects of optogenetic activation on the clinical symptoms of DSS-induced colitis in the ChR2/TH mice (weight loss: $p = 0.0470$ for isotype control group and $p = 0.2939$ for anti-MAdCAM-1 group; colon shortening: $p = 0.0015$ for isotype control group, and $p = 0.1654$ for anti-MAdCAM-1 group; histological score: $p = 0.3761$ for anti MAdCAM-1 group; Figures 5B–5E). Moreover, the anti-MAdCAM-1 antibody abolished the effects of optogenetic activation on immune cells abundance in the DSS-treated ChR2/TH mice (LP: isotype control $p = 0.0156$ and anti-MAdCAM-1 $p = 0.7278$; IEL: isotype control $p = 0.0466$ and anti-MAdCAM-1 $p = 0.8156$; Figure 5F). Taken together, in the absence of MAdCAM-1, the change in the clinical symptoms and immune abundance following optogenetic activation is eliminated. Therefore, we conclude that the change in endothelial MAdCAM-1 levels is necessary for the observed beneficial effect of the optogenetic sympathetic activation on DSS-induced colitis.

DISCUSSION

The SNS is a major communication pathway between the brain and the periphery and plays an important role in immune regulation, especially during stress. Although the SNS is composed of two distinct anatomical arms, most research has been focused on the effects of the systemic, endocrine arm of the SNS, while neglecting the local arm. Conceptually, these two SNS pathways may serve different physiological functions and might even balance each other's activity. The endocrine arm can act as an alarm system that increases inflammation, while the local arm, based on our findings, appears to attenuate the inflammatory response. Here, we demonstrate this differential effect in the context of DSS-induced colitis. While both systemic and local manipulations are associated with an increase in NA levels, the responses to these manipulations may differ both in the levels of secreted NA (Bell and Gillespie, 1981) and the target cells affected. For example, while NA secreted to the blood affects many organs simultaneously, the local sympathetic fibers mainly affect the target site (e.g., the colon).

The unique effects of such local SNS innervations have become increasingly apparent. Sympathetic innervations to the bone marrow were shown to regulate hematopoietic stem cell egress to the blood in response to circadian signals from the brain (Maryanovich et al., 2018). Activity of sympathetic neurons upregulate clearance of NA via sympathetic neuron-associated macrophages, and modulate changes in brown adipose tissue content, thermogenesis and weight in mice (Pirzgalska et al., 2017). Such effects of the SNS can be achieved mainly by its local arm, which offers unique features. For example, local signaling allows for elevated levels of NA to be attained within specific sites without affecting the entire organism. Moreover, guiding cell migration to specific organs (e.g., colon, bone marrow) requires the use of localized signals. The effects we have shown of local sympathetic activation on endothelial MAdCAM-1 expression provide an effective mechanism for such localized control of immunity, especially since anatomically, blood vessels are innervated by sympathetic neurons (Anlauf et al., 2003; Wehrwein et al., 2016).

Taken together, the emerging evidence indicating that the brain engages in precise spatial and temporal control over the peripheral immune system is supported by the infrastructure provided by the SNS. Physiologically, both local and endocrine components of the SNS can be used by the organism to regulate homeostasis; however, research and clinical applications have focused on the endocrine-systemic pathway, often neglecting the local pathway. Here, we show that the local sympathetic fibers engage in unique control of the endothelial gateway between the circulation and the tissue. A better understanding of these interactions may allow us to utilize targeted neuronal activation to treat local inflammatory conditions.

Limitations of the study

The use of optogenetics allowed us to manipulate specific neuronal populations with spatial and temporal resolution. We generated transgenic mice, which express the optogenetic channel in TH^+ cells, using a *Cre*-dependent system. This allowed us to specifically manipulate the sympathetic neurons in the colon, and we attributed the effects on endothelial cells to the main neurotransmitter secreted by these cells, NA. However, it is possible that other factors, specifically neuropeptides co-released by the sympathetic neurons (e.g., neuropeptide Y; Reichmann and Holzer, 2016; Zukowska-Grojec et al., 1998), were induced by the optogenetic manipulation and may also contribute to the observed effects. Moreover, while the optogenetic manipulation was local to the colon, it was not specifically limited to the nerve fibers enveloping blood vessels. Thus, further analysis of the interactions between the sympathetic fibers, endothelial, and immune cells is required.

STAR★METHODS

Detailed methods are provided in the online version of this paper and include the following:

- KEY RESOURCES TABLE
- RESOURCE AVAILABILITY
  - Lead contact
  - Materials availability
  - Data and code availability

CellPress
OPEN ACCESS

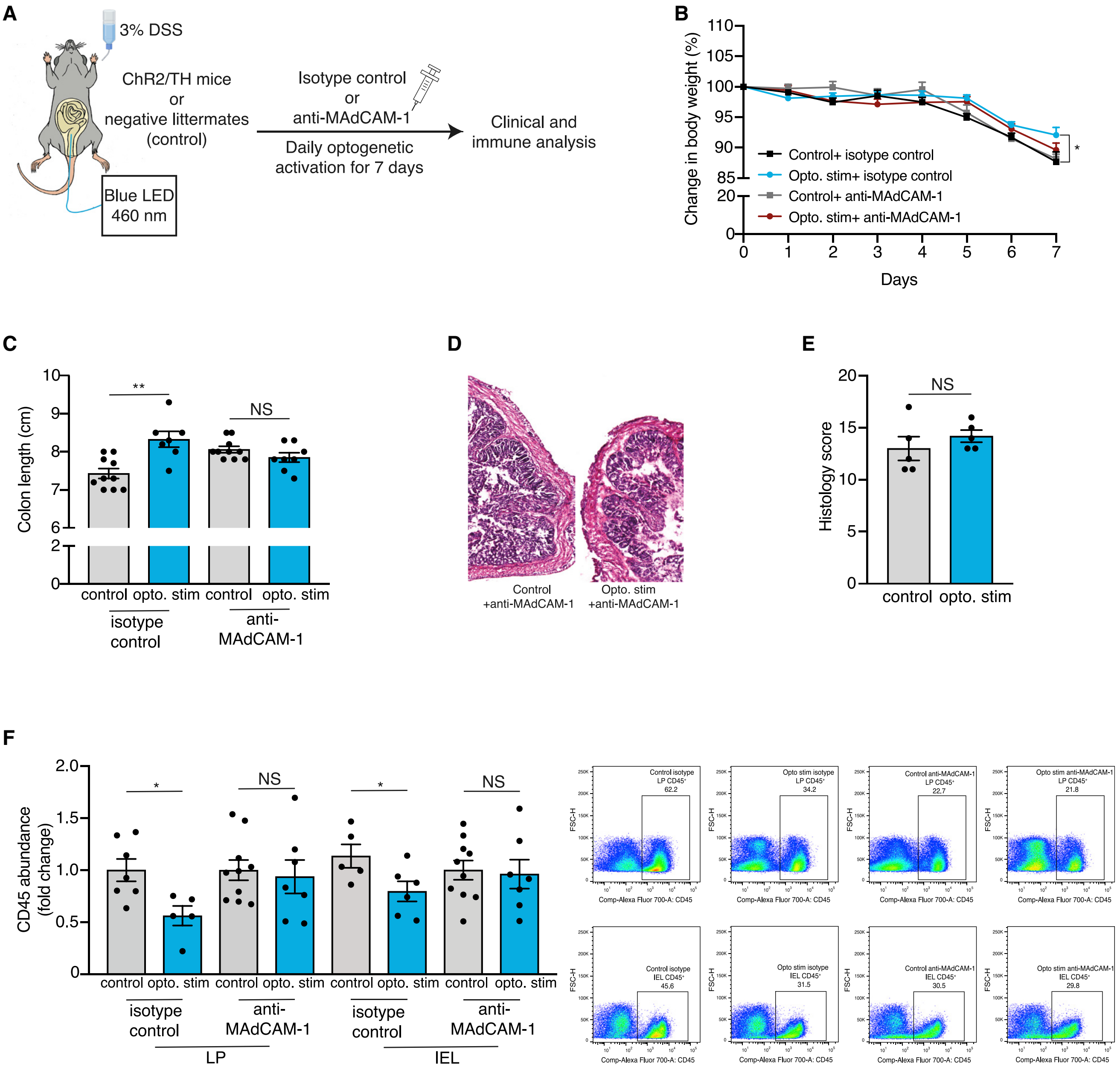

Figure 5. MAdCAM-1 is necessary to mediate the beneficial effects of local sympathetic activation on DSS-induced colitis

(A) Experimental design: ChR2/TH mice and their controls (transgene negative littermates exposed to light stimulation) underwent daily optogenetic activation of their colon (as described in Figure 1E) and were supplemented with 3% DSS for 7 days. Two sets of groups were used: ChR2/TH mice and their controls that received daily IP injection of anti-MAdCAM-1 antibody (anti-MAdCAM-1 group) or daily IP injection of isotype control antibody (isotype control group).

(B) Change in body weight of ChR2/TH mice and their controls following 7 days of 3% DSS and daily optogenetic activation. The mice received daily injections of anti-MAdCAM-1 or isotype control. Isotype control: N = 10, 10; anti-MAdCAM-1: N = 10, 8.

(C) Colon length of ChR2/TH mice and their controls following 7 days of 3% DSS and daily optogenetic activation. The mice received daily injections of anti-MAdCAM-1 or isotype control. Isotype control: N = 10, 7; anti-MAdCAM-1: N = 10, 8.

(D and E) Representative histological image (D) and histological severity score (E) of colons obtained from ChR2/TH mice and their controls following 7 days of 3% DSS, daily optogenetic activation and daily injections of anti-MAdCAM-1. N = 5, 5.

(F) Left: fold change in abundance of total immune cells (number of $CD45^+$ cells/gr colon) in the LP and IEL layers of the colon from ChR2/TH mice and their controls following 7 days of 3% DSS and daily optogenetic activation. The mice received daily injections of anti-MAdCAM-1 or isotype control. Fold change between the ChR2/TH mice and their controls relative to the mean of the control in each group (the isotype control group and anti-MAdCAM-1 group). LP tissue: N = 7, 5 isotype control, N = 10, 7 anti-MAdCAM-1. IEL tissue: N = 5, 6 isotype control, N = 10, 7 anti-MAdCAM-1. Right: representative flow cytometry plot demonstrating the percentage of $CD45^+$ population in the LP and in the IEL. Mean ± SEM, as well as individual mice, are presented for each group. Student's unpaired t test. * = $p < 0.05$, ** = $p < 0.01$. Data represent at least two independent repeats.

- EXPERIMENTAL MODEL AND SUBJECT DETAILS
  - Mice models
  - Primary colon cell culture
- METHOD DETAILS
  - Optogenetic manipulation
  - DSS-induced colitis model
  - Histology staining and scoring
  - Immunohistochemical tissue analysis
  - Analysis of noradrenaline levels
  - Calcium indicator
  - Flow cytometry
  - Noradrenaline injection
  - Nadolol (β-adrenergic blocker) and Phentolamine (α-adrenergic blocker) injection
  - *Ex vivo* assays
  - Sympathetic denervation
  - Anti MAdCAM-1 treatment
  - Activity monitoring
  - Quantitative RT-PCR
  - Illustrations
- QUANTIFICATION AND STATISTICAL ANALYSIS

SUPPLEMENTAL INFORMATION

Supplemental information can be found online at https://doi.org/10.1016/j.immuni.2021.04.007.

ACKNOWLEDGMENTS

We thank O. Barak for "asking the right question," helpful discussions and for commenting on the manuscript; I. Kahn for the optogenetics equipment; O. Izhar and A. Aronhiem for sharing mice; A. Monsonego for assisting with immunohistochemistry; and M. Schwartz, M. Rivlin, I. Goshen, D. Farfara and S. Berger for helpful discussions. We are grateful to J. Schiller, B. Maman, and S. Schwartz for their advice and help with the calcium imaging experiments and S. Berlin and R. Heinrich for their help with the clearing technique. We thank S. Schwarzbaum and U. Rolls for editing the manuscript and O. Shenker, A. Grau, E. Suss-Toby, M. Holdengreber and M. Gurewitz for technical support. We are grateful to the Adelis Foundation, the Allen and Jewel Prince Center for Neurodegenerative Processes of the Brain, and Colleck Research Fund for their support. This project has received funding from the European Commission Horizon 2020 research and innovation program under grant agreement no. 758952-ERC-2017-STG (NEIMO). A.R is an international Howard Hughes Medical Institute HHMI-Wellcome Trust scholar.

AUTHOR CONTRIBUTIONS

M.S. Designed and carried out all the experiments, interpreted the results, and wrote the manuscript. H.A. and N.B. contributed to the experimental design, execution of the experiments and contributed to data analysis. Y.E. contributed to the immunohistochemistry analysis. B.K and T.B.S contributed to the execution of the experiments and data analysis. T.K. and M.K. contributed to the execution of the experiments. F.H. contributed to the results interpretation. A.R. contributed to the experimental design, interpretation of results, and wrote the manuscript. All authors reviewed the final manuscript.

DECLARATION OF INTERESTS

The authors declare no competing interests.

Received: May 12, 2020
Revised: January 16, 2021
Accepted: April 9, 2021
Published: April 30, 2021

REFERENCES

Altschuler, S.M., Escardo, J., Lynn, R.B., and Miselis, R.R. (1993). The central organization of the vagus nerve innervating the colon of the rat. Gastroenterology *104*, 502–509.

Ando, T., Langley, R.R., Wang, Y., Jordan, P.A., Minagar, A., Alexander, J.S., and Jennings, M.H. (2007). Inflammatory cytokines induce MAdCAM-1 in murine hepatic endothelial cells and mediate alpha-4 beta-7 integrin dependent lymphocyte endothelial adhesion in vitro. BMC Physiol. *7*, 10.

Anlauf, M., Schäfer, M.K.-H., Eiden, L., and Weihe, E. (2003). Chemical coding of the human gastrointestinal nervous system: cholinergic, VIPergic, and catecholaminergic phenotypes. J. Comp. Neurol. *459*, 90–111.

Bai, A., Lu, N., Guo, Y., Chen, J., and Liu, Z. (2009). Modulation of inflammatory response via α2-adrenoceptor blockade in acute murine colitis. Clin. Exp. Immunol. *156*, 353–362.

Başar, E. (2011). Brain-Body-Mind in the Nebulous Cartesian System: A Holistic Approach by Oscillations (Springer New York).

Bell, C., and Gillespie, J.S. (1981). Dopamine and noradrenaline levels in peripheral tissues of several mammalian species. J. Neurochem. *36*, 703–706.

Bellono, N.W., Bayrer, J.R., Leitch, D.B., Castro, J., Zhang, C., O'Donnell, T.A., Brierley, S.M., Ingraham, H.A., and Julius, D. (2017). Enterochromaffin Cells Are Gut Chemosensors that Couple to Sensory Neural Pathways. Cell *170de*, 185–198.e16.

Ben-Shaanan, T.L., Azulay-Debby, H., Dubovik, T., Starosvetsky, E., Korin, B., Schiller, M., Green, N.L., Admon, Y., Hakim, F., Shen-Orr, S.S., and Rolls, A. (2016). Activation of the reward system boosts innate and adaptive immunity. Nat. Med. *22*, 940–944.

Ben-Shaanan, T.L., Schiller, M., Azulay-Debby, H., Korin, B., Boshnak, N., Koren, T., Krot, M., Shakya, J., Rahat, M.A., Hakim, F., and Rolls, A. (2018). Modulation of anti-tumor immunity by the brain's reward system. Nat. Commun. *9*, 2723.

Berlin, C., Berg, E.L., Briskin, M.J., Andrew, D.P., Kilshaw, P.J., Holzmann, B., Weissman, I.L., Hamann, A., and Butcher, E.C. (1993). Alpha 4 beta 7 integrin mediates lymphocyte binding to the mucosal vascular addressin MAdCAM-1. Cell *74*, 185–195.

Blaugrund, E., Pham, T.D., Tennyson, V.M., Lo, L., Sommer, L., Anderson, D.J., and Gershon, M.D. (1996). Distinct subpopulations of enteric neuronal progenitors defined by time of development, sympathoadrenal lineage markers and Mash-1-dependence. Development *122*, 309 LP–320.

Briskin, M., Winsor-Hines, D., Shyjan, A., Cochran, N., Bloom, S., Wilson, J., McEvoy, L.M., Butcher, E.C., Kassam, N., Mackay, C.R., et al. (1997). Human mucosal addressin cell adhesion molecule-1 is preferentially expressed in intestinal tract and associated lymphoid tissue. Am. J. Pathol. *151*, 97–110.

Browning, K.N., and Travagli, R.A. (2014). Central Nervous System Control of Gastrointestinal Motility and Secretion and Modulation of Gastrointestinal Functions. In Comprehensive Physiology (John Wiley & Sons, Inc.), pp. 1339–1368.

Brumovsky, P., Villar, M.J., and Hökfelt, T. (2006). Tyrosine hydroxylase is expressed in a subpopulation of small dorsal root ganglion neurons in the adult mouse. Exp. Neurol. *200*, 153–165.

Chassaing, B., Aitken, J.D., Malleshappa, M., and Vijay-Kumar, M. (2014). Dextran Sulfate Sodium (DSS)-Induced Colitis in Mice. In Current Protocols in Immunology (John Wiley & Sons, Inc.), pp. 15.25.1–15.25.14.

Cheroutre, H., Lambolez, F., and Mucida, D. (2011). The light and dark sides of intestinal intraepithelial lymphocytes. Nat. Rev. Immunol. *11*, 445–456.

Cohen, J.A., Edwards, T.N., Liu, A.W., Hirai, T., Jones, M.R., Wu, J., Li, Y., Zhang, S., Ho, J., Davis, B.M., et al. (2019). Cutaneous TRPV1^{+} Neurons Trigger Protective Innate Type 17 Anticipatory Immunity. Cell *178*, 919–932.e14.

Coombes, J.L., and Powrie, F. (2008). Dendritic cells in intestinal immune regulation. Nat. Rev. Immunol. *8*, 435–446.

Cosentino, M., Fietta, A.M., Ferrari, M., Rasini, E., Bombelli, R., Carcano, E., Saporiti, F., Meloni, F., Marino, F., and Lecchini, S. (2007). Human

CellPress
OPEN ACCESS

CD4+CD25+ regulatory T cells selectively express tyrosine hydroxylase and contain endogenous catecholamines subserving an autocrine/paracrine inhibitory functional loop. Blood *109*, 632–642.

Cryan, J.F., and Dinan, T.G. (2012). Mind-altering microorganisms: the impact of the gut microbiota on brain and behaviour. Nat. Rev. Neurosci. *13*, 701–712.

Dantzer, R. (2018). Neuroimmune Interactions: From the Brain to the Immune System and Vice Versa. Physiol. Rev. *98*, 477–504.

Daubner, S.C., Le, T., and Wang, S. (2011). Tyrosine hydroxylase and regulation of dopamine synthesis. Arch. Biochem. Biophys. *508*, 1–12.

Deisseroth, K. (2011). Optogenetics. Nat. Methods *8*, 26–29.

Dhabhar, F.S., and McEwen, B.S. (1997). Acute stress enhances while chronic stress suppresses cell-mediated immunity in vivo: a potential role for leukocyte trafficking. Brain Behav. Immun. *11*, 286–306.

Dhabhar, F.S., Malarkey, W.B., Neri, E., and McEwen, B.S. (2012). Stress-induced redistribution of immune cells–from barracks to boulevards to battlefields: a tale of three hormones–Curt Richter Award winner. Psychoneuroendocrinology *37*, 1345–1368.

Druzd, D., Matveeva, O., Ince, L., Harrison, U., He, W., Schmal, C., Herzel, H., Tsang, A.H., Kawakami, N., Leliavski, A., et al. (2017). Lymphocyte Circadian Clocks Control Lymph Node Trafficking and Adaptive Immune Responses. Immunity *46*, 120–132.

Eichele, D.D., and Kharbanda, K.K. (2017). Dextran sodium sulfate colitis murine model: An indispensable tool for advancing our understanding of inflammatory bowel diseases pathogenesis. World J. Gastroenterol. *23*, 6016–6029.

Elenkov, I.J., Wilder, R.L., Chrousos, G.P., and Vizi, E.S. (2000). The sympathetic nerve–an integrative interface between two supersystems: the brain and the immune system. Pharmacol. Rev. *52*, 595–638.

Fagarasan, S., and Honjo, T. (2003). Intestinal IgA synthesis: regulation of front-line body defences. Nat. Rev. Immunol. *3*, 63–72.

Felten, D.L., Felten, S.Y., Carlson, S.L., Olschowka, J.A., and Livnat, S. (1985). Noradrenergic and peptidergic innervation of lymphoid tissue. J. Immunol. *135* (2, Suppl), 755s–765s.

Flierl, M.A., Rittirsch, D., Huber-Lang, M., Sarma, J.V., and Ward, P.A. (2008). Catecholamines-crafty weapons in the inflammatory arsenal of immune/inflammatory cells or opening pandora's box? Mol. Med. *14*, 195–204.

Foster, J.A., and McVey Neufeld, K.-A. (2013). Gut-brain axis: how the microbiome influences anxiety and depression. Trends Neurosci. *36*, 305–312.

Gabanyi, I., Muller, P.A., Feighery, L., Oliveira, T.Y., Costa-Pinto, F.A., and Mucida, D. (2016). Neuro-immune Interactions Drive Tissue Programming in Intestinal Macrophages. Cell *164*, 378–391.

Godbout, J.P., and Glaser, R. (2006). Stress-induced immune dysregulation: implications for wound healing, infectious disease and cancer. J. Neuroimmune Pharmacol. *1*, 421–427.

Gracie, D.J., Hamlin, P.J., and Ford, A.C. (2019). The influence of the brain-gut axis in inflammatory bowel disease and possible implications for treatment. Lancet Gastroenterol. Hepatol. *4*, 632–642.

Guyenet, P.G. (2006). The sympathetic control of blood pressure. Nat. Rev. Neurosci. *7*, 335–346.

Habtezion, A., Nguyen, L.P., Hadeiba, H., and Butcher, E.C. (2016). Leukocyte Trafficking to the Small Intestine and Colon. Gastroenterology *150*, 340–354.

Hadis, U., Wahl, B., Schulz, O., Hardtke-Wolenski, M., Schippers, A., Wagner, N., Müller, W., Sparwasser, T., Förster, R., and Pabst, O. (2011). Intestinal tolerance requires gut homing and expansion of FoxP3+ regulatory T cells in the lamina propria. Immunity *34*, 237–246.

Han, W., Tellez, L.A., Perkins, M.H., Perez, I.O., Qu, T., Ferreira, J., Ferreira, T.L., Quinn, D., Liu, Z.-W., Gao, X.-B., et al. (2018). A Neural Circuit for Gut-Induced Reward. Cell *175*, 887–888.

Hart, A., and Kamm, M.A. (2002). Review article: mechanisms of initiation and perpetuation of gut inflammation by stress. Aliment. Pharmacol. Ther. *16*, 2017–2028.

Jänig, W. (2014). Sympathetic nervous system and inflammation: a conceptual view. Auton. Neurosci. *182*, 4–14.

Johnson, J.D., Campisi, J., Sharkey, C.M., Kennedy, S.L., Nickerson, M., Greenwood, B.N., and Fleshner, M. (2005). Catecholamines mediate stress-induced increases in peripheral and central inflammatory cytokines. Neuroscience *135*, 1295–1307.

Kaelberer, M.M., Buchanan, K.L., Klein, M.E., Barth, B.B., Montoya, M.M., Shen, X., and Bohórquez, D.V. (2018). A gut-brain neural circuit for nutrient sensory transduction. Science *361*, eaat5236.

Karasawa, N., Arai, R., Isomura, G., Nagatsu, T., and Nagatsu, I. (1997). Coexistence of tyrosine hydroxylase and serotonin in the raphe nucleus of the laboratory shrew (Suncus murinus) during postnatal life. Brain Res. Dev. Brain Res. *99*, 121–125.

Koelink, P.J., Wildenberg, M.E., Stitt, L.W., Feagan, B.G., Koldijk, M., van 't Wout, A.B., Atreya, R., Vieth, M., Brandse, J.F., Duijst, S., et al. (2018). Development of Reliable, Valid and Responsive Scoring Systems for Endoscopy and Histology in Animal Models for Inflammatory Bowel Disease. J. Crohn's Colitis *12*, 794–803.

Kostrzewa, R.M., and Jacobowitz, D.M. (1974). Pharmacological actions of 6-hydroxydopamine. Pharmacol. Rev. *26*, 199–288.

Kubota, A., Ootsuka, Y., Xu, T., and Terui, N. (1995). The 10-Hz rhythm in the sympathetic nerve activity of cats, rats and rabbits. Neurosci. Lett. *196*, 173–176.

Kuhar, M.J., Couceyro, P.R., and Lambert, P.D. (1999). Biosynthesis of Catecholamines. Lawson, C., and Wolf, S. ICAM-1 signaling in endothelial cells. Pharmacol. Rep. *61*, 22–32.

Lawson, C., and Wolf, S. (2009). ICAM-1 signaling in endothelial cells. Pharmacological Reports *61*, 22–32.

Liu, Y.-Z., Wang, Y.-X., and Jiang, C.-L. (2017). Inflammation: The Common Pathway of Stress-Related Diseases. Front. Hum. Neurosci. *11*, 316.

Lorton, D., and Bellinger, D.L. (2015). Molecular mechanisms underlying β-adrenergic receptor-mediated cross-talk between sympathetic neurons and immune cells. Int. J. Mol. Sci. *16*, 5635–5665.

Mackay, C.R., and Imhof, B.A. (1993). Cell adhesion in the immune system. Immunol. Today *14*, 99–102.

Marra, S., Burnett, M., and Hoffman-Goetz, L. (2005). Intravenous catecholamine administration affects mouse intestinal lymphocyte number and apoptosis. J. Neuroimmunol. *158*, 76–85.

Maryanovich, M., Zahalka, A.H., Pierce, H., Pinho, S., Nakahara, F., Asada, N., Wei, Q., Wang, X., Ciero, P., Xu, J., et al. (2018). Adrenergic nerve degeneration in bone marrow drives aging of the hematopoietic stem cell niche. Nat. Med. *24*, 782–791.

Mawdsley, J.E., and Rampton, D.S. (2005). Psychological stress in IBD: new insights into pathogenic and therapeutic implications. Gut *54*, 1481–1491.

Meenan, J., Spaans, J., Grool, T.A., Pals, S.T., Tytgat, G.N., and van Deventer, S.J. (1997). Altered expression of alpha 4 beta 7, a gut homing integrin, by circulating and mucosal T cells in colonic mucosal inflammation. Gut *40*, 241–246.

Montgomery, K.L., Iyer, S.M., Christensen, A.J., Deisseroth, K., and Delp, S.L. (2016). Beyond the brain: Optogenetic control in the spinal cord and peripheral nervous system. Sci. Transl. Med. *8*, 337rv5.

Morey, J.N., Boggero, I.A., Scott, A.B., and Segerstrom, S.C. (2015). Current directions in stress and human immune function. Curr. Opin. Psychol. *5*, 13–17.

Nance, D.M., and Sanders, V.M. (2007). Autonomic innervation and regulation of the immune system (1987-2007). Brain Behav. Immun. *21*, 736–745.

Natale, G., Ryskalin, L., Busceti, C.L., Biagioni, F., and Fornai, F. (2017). The nature of catecholamine-containing neurons in the enteric nervous system in relationship with organogenesis, normal human anatomy and neurodegeneration. Arch. Ital. Biol. *155*, 118–130.

Nguyen, K.D., Qiu, Y., Cui, X., Goh, Y.P.S., Mwangi, J., David, T., Mukundan, L., Brombacher, F., Locksley, R.M., and Chawla, A. (2011). Alternatively activated macrophages produce catecholamines to sustain adaptive thermogenesis. Nature *480*, 104–108.

Obermayr, F., Stamp, L.A., Anderson, C.R., and Young, H.M. (2013). Genetic fate-mapping of tyrosine hydroxylase-expressing cells in the enteric nervous system. Neurogastroenterol. Motil. *25*, e283–e291.

Ogawa, H., Binion, D.G., Heidemann, J., Theriot, M., Fisher, P.J., Johnson, N.A., Otterson, M.F., and Rafiee, P. (2005). Mechanisms of MAdCAM-1 gene expression in human intestinal microvascular endothelial cells. Am. J. Physiol. Cell Physiol. *288*, C272–C281.

Ootsuka, Y., Xu, T., and Terui, N. (1995). The spinally mediated 10-Hz rhythm in the sympathetic nerve activity of cats. J. Auton. Nerv. Syst. *54*, 89–103.

Pirzgalska, R.M., Seixas, E., Seidman, J.S., Link, V.M., Sánchez, N.M., Mahú, I., Mendes, R., Gres, V., Kubasova, N., Morris, I., et al. (2017). Sympathetic neuron-associated macrophages contribute to obesity by importing and metabolizing norepinephrine. Nat. Med. *23*, 1309–1318.

Pongratz, G., and Straub, R.H. (2014). The sympathetic nervous response in inflammation. Arthritis Res. Ther. *16*, 504.

Pullen, N., Molloy, E., Carter, D., Syntin, P., Clemo, F., Finco-Kent, D., Reagan, W., Zhao, S., Kawabata, T., and Sreckovic, S. (2009). Pharmacological characterization of PF-00547659, an anti-human MAdCAM monoclonal antibody. Br. J. Pharmacol. *157*, 281–293.

Reichmann, F., and Holzer, P. (2016). Neuropeptide Y: A stressful review. Neuropeptides *55*, 99–109.

Rinner, I., Schauenstein, K., Mangge, H., Porta, S., and Kvetnansky, R. (1992). Opposite effects of mild and severe stress on in vitro activation of rat peripheral blood lymphocytes. Brain Behav. Immun. *6*, 130–140.

Sanders, V.M., and Straub, R.H. (2002). Norepinephrine, the beta-adrenergic receptor, and immunity. Brain Behav. Immun. *16*, 290–332.

Saunders, P.R., Miceli, P., Vallance, B.A., Wang, L., Pinto, S., Tougas, G., Kamath, M., and Jacobson, K. (2006). Noradrenergic and cholinergic neural pathways mediate stress-induced reactivation of colitis in the rat. Auton. Neurosci. *124*, 56–68.

Scanzano, A., and Cosentino, M. (2015). Adrenergic regulation of innate immunity: a review. Front. Pharmacol. *6*, 171.

Scheiermann, C., Kunisaki, Y., Lucas, D., Chow, A., Jang, J.-E., Zhang, D., Hashimoto, D., Merad, M., and Frenette, P.S. (2012). Adrenergic nerves govern circadian leukocyte recruitment to tissues. Immunity *37*, 290–301.

Schultz, W. (1998). Predictive reward signal of dopamine neurons. J. Neurophysiol. *80*, 1–27.

Sheng, Y., and Zhu, L. (2018). The crosstalk between autonomic nervous system and blood vessels. Int. J. Physiol. Pathophysiol. Pharmacol. *10*, 17–28.

Sloan, E.K., Capitanio, J.P., Tarara, R.P., Mendoza, S.P., Mason, W.A., and Cole, S.W. (2007). Social stress enhances sympathetic innervation of primate lymph nodes: mechanisms and implications for viral pathogenesis. J. Neurosci. *27*, 8857–8865.

Springer, T.A. (1990). Adhesion receptors of the immune system. Nature *346*, 425–434.

Steptoe, A., Hamer, M., and Chida, Y. (2007). The effects of acute psychological stress on circulating inflammatory factors in humans: a review and meta-analysis. Brain Behav. Immun. *21*, 901–912.

Straub, R.H., Wiest, R., Strauch, U.G., Härle, P., and Schölmerich, J. (2006). The role of the sympathetic nervous system in intestinal inflammation. Gut *55*, 1640–1649.

Suzuki, K., Hayano, Y., Nakai, A., Furuta, F., and Noda, M. (2016). Adrenergic control of the adaptive immune response by diurnal lymphocyte recirculation through lymph nodes. J. Exp. Med. *213*, 2567–2574.

Takahashi, A., Flanigan, M.E., McEwen, B.S., and Russo, S.J. (2018). Aggression, Social Stress, and the Immune System in Humans and Animal Models. Front. Behav. Neurosci. *12*, 56.

Taylor, C.T., and Keely, S.J. (2007). The autonomic nervous system and inflammatory bowel disease. Auton. Neurosci. *133*, 104–114.

Tsai, H.C., Zhang, F., Adamantidis, A., Stuber, G.D., Bond, A., De Lecea, L., and Deisseroth, K. (2009). Phasic firing in dopaminergic neurons is sufficient for behavioral conditioning. Science *324*, 1080–1084.

Varol, C., Vallon-Eberhard, A., Elinav, E., Aychek, T., Shapira, Y., Luche, H., Fehling, H.J., Hardt, W.-D., Shakhar, G., and Jung, S. (2009). Intestinal lamina propria dendritic cell subsets have different origin and functions. Immunity *31*, 502–512.

Varol, C., Zigmond, E., and Jung, S. (2010). Securing the immune tightrope: mononuclear phagocytes in the intestinal lamina propria. Nat. Rev. Immunol. *10*, 415–426.

Veiga-Fernandes, H., and Mucida, D. (2016). Neuro-Immune Interactions at Barrier Surfaces. Cell *165*, 801–811.

Vermeire, S., Sandborn, W.J., Danese, S., Hébuterne, X., Salzberg, B.A., Klopocka, M., Tarabar, D., Vanasek, T., Greguš, M., Hellstern, P.A., et al. (2017). Anti-MAdCAM antibody (PF-00547659) for ulcerative colitis (TURANDOT): a phase 2, randomised, double-blind, placebo-controlled trial. Lancet *390*, 135–144.

Viswanathan, K., and Dhabhar, F.S. (2005). Stress-induced enhancement of leukocyte trafficking into sites of surgery or immune activation. Proc. Natl. Acad. Sci. USA *102*, 5808–5813.

von Andrian, U.H., and Mempel, T.R. (2003). Homing and cellular traffic in lymph nodes. Nat. Rev. Immunol. *3*, 867–878.

Wehrwein, E.A., Orer, H.S., and Barman, S.M. (2016). Overview of the Anatomy, Physiology, and Pharmacology of the Autonomic Nervous System. In Comprehensive Physiology (John Wiley & Sons, Inc.), pp. 1239–1278.

Wong, C.H.Y., Jenne, C.N., Lee, W.-Y., Leger, C., and Kubes, P. (2011). Functional Innervation of Hepatic iNKT Cells Is Immunosuppressive Following Stroke. Science *334*, 101–105.

Xie, F., Li, B.X., Kassenbrock, A., Xue, C., Wang, X., Qian, D.Z., Sears, R.C., and Xiao, X. (2015). Identification of a Potent Inhibitor of CREB-Mediated Gene Transcription with Efficacious in Vivo Anticancer Activity. J. Med. Chem. *58*, 5075–5087.

Zádori, Z.S., Tóth, V.E., Fehér, Á., Al-Khrasani, M., Puskár, Z., Kozsurek, M., Timár, J., Tábi, T., Helyes, Z., Hein, L., et al. (2016). Inhibition of α2A-Adrenoceptors Ameliorates Dextran Sulfate Sodium-Induced Acute Intestinal Inflammation in Mice. J. Pharmacol. Exp. Ther. *358*, 483–491.

Zhang, L., Wang, C., Xi, Z., Li, D., and Xu, Z. (2018). Mercaptoethanol Protects the Aorta from Dissection by Inhibiting Oxidative Stress, Inflammation, and Extracellular Matrix Degeneration in a Mouse Model. Med. Sci. Monit. *24*, 1802–1812.

Zukowska-Grojec, Z., Karwatowska-Prokopczuk, E., Rose, W., Rone, J., Movafagh, S., Ji, H., Yeh, Y., Chen, W.T., Kleinman, H.K., Grouzmann, E., and Grant, D.S. (1998). Neuropeptide Y: a novel angiogenic factor from the sympathetic nerves and endothelium. Circ. Res. *83*, 187–195.

CellPress
OPEN ACCESS

STAR★METHODS

KEY RESOURCES TABLE

REAGENT or RESOURCE	SOURCE	IDENTIFIER
Antibodies		
Sheep polyclonal anti-TH	Abcam	Cat# ab113; RRID: AB_297905
Chicken anti-GFP	Abcam	Cat# ab13970; RRID: AB_300798
Rabbit anti-β3 tubulin	Abcam	Cat# ab18207; RRID: AB_444319
Rat anti-DAT	Abcam	Cat# ab5990; RRID: AB_305226
Goat anti-serotonin	Abcam	Cat# ab66047; RRID: AB_1142794
Rabbit anti-SERT	Millipore	Cat# PC177L; RRID: AB_2122553
Goat anti-CD31	R&D systems	Cat# AF3628; RRID: AB_2161028
Rabbit anti-Ki67	Abcam	Cat# ab15580; RRID: AB_443209
Alexa Flour 700-conjugated anti-CD45	Biolegend	Cat# 103128; RRID: AB_493715
BV605-conjugated anti-CD45	Biolegend	Cat# 103140; RRID: AB_2562342
APC-conjugated anti-CD45	Biolegend	Cat# 103112; RRID: AB_312977
PE-conjugated anti-γ-δ	Biolegend	Cat# 118108; RRID: AB_313832
PB-conjugated anti-TCRβ	Biolegend	Cat# 109226; RRID: AB_1027649
BV510-conjugated anti-IL-17	Biolegend	Cat# 506933; RRID: AB_2562668
PE-Cy7- conjugated anti-CD4	Biolegend	Cat# 100422; RRID: AB_312707
APC-conjugated anti-CD4	Biolegend	Cat # 100412; RRID: AB_312697
AF488-conjugated anti-FOXP3	Biolegend	Cat # 320012; RRID: AB_439748
BV650-conjugated anti-NKp46	Biolegend	Cat# 137635; RRID: AB_2734200
PB-conjugated anti-CD3	Biolegend	Cat# 100334; RRID: AB_2028475
BV605-conjugated anti CD3	Biolegend	Cat# 100351; RRID: AB_2565842
PerCP-conjugated anti-CD8	Biolegend	Cat# 100732; RRID: AB_893423
BV510-conjugated anti-CD127	Biolegend	Cat# 135033; RRID: AB_2564576
PB-conjugated anti-T-bet	Biolegend	Cat# 644808; RRID: AB_1595479
PerCP-Cy5.5-conjugated anti-GATA3	Biolegend	Cat# 653812; RRID: AB_2563219
PE-conjugated anti-RORγ	eBioscience	Cat# 12-6981-82; RRID: AB_10807092
BV605-conjugated anti-CD62L	Biolegend	Cat# 104438; RRID: AB_2563058
BV650-conjugated anti-CD44	Biolegend	Cat# 103049; RRID: AB_2562600
PerCP-Cy5.5-conjugated anti-CD11b	Biolegend	Cat# 101228; RRID: AB_893232
PECy7-conjugated anti-CD11c	Biolegend	Cat# 117318; RRID: AB_493568
BV605-conjugated anti-CD103	Biolegend	Cat# 121433; RRID: AB_2629724
BV510-conjugated anti LY6C	Biolegend	Cat# 128033; RRID: AB_2562351
APC-conjugated anti-LY6G	Biolegend	Cat# 127614; RRID: AB_2227348
BV650-conjugated anti CX3CR1	Biolegend	Cat# 149033; RRID: AB_2565999
PECy7-conjugated anti-CD19	Biolegend	Cat# 115520; RRID: AB_313655
BV510-conjugated anti-CD27	Biolegend	Cat# 124229; RRID: AB_2565795
BV650-conjugated anti-CD138	Biolegend	Cat# 142518; RRID: AB_2650927
PerCP-conjugated anti-IgG	Biolegend	Cat# 405334; RRID: AB_2687005
APC-conjugated anti-IgM	Biolegend	Cat# 406509; RRID: AB_315059
Biotin-conjugated anti-IgA	Biolegend	Cat# 407004; RRID: AB_315079
FITC-conjugated anti-IgD	Biolegend	Cat# 405704; RRID: AB_315026
PE conjugated anti-ICAM-1	Biolegend	Cat# 116108; RRID: AB_313699
Biotin-conjugated anti-MAdCAM-1	Biolegend	Cat# 120706; RRID: AB_493397
AF488-conjugated anti-MAdCAM-1	Biolegend	Cat# 120708; RRID: AB_493398
PECy7-conjugated anti-CD31	Biolegend	Cat# 102418; RRID: AB_830757

(*Continued on next page*)

Continued

REAGENT or RESOURCE	SOURCE	IDENTIFIER
Biotin-conjugated anti-CD69	Biolegend	Cat# 104504; RRID: AB_313107
PE-conjugated anti-TNF-α	Biolegend	Cat# 506306; RRID: AB_315427
Biotin-conjugated anti-CD86	Biolegend	Cat# 105004; RRID; AB_313147
PE-conjugated anti-CD80	Biolegend	Cat# 104707; RRID: AB_313128
AF488-conjugated streptavidin	Jackson	Cat# 016-540-084; RRID: AB_2337249
PerCP-conjugated streptavidin	Jackson	Cat# 016-120-084; RRID: AB_2337241
BV605-conjugated streptavidin	Biolegend	Cat# 405229
PE-conjugated phospho-CREB	Cell Signaling	Cat# 14228; RRID: AB_2798432
Zombie NIR dye	Biolegend	Cat# 423106
anti-MAdCAM-1 antibody	Bio X Cell	Cat# BE0035; RRID: 1107725
IgG2a isotype control	Bio X Cell	Cat# BE0089; RRID: AB_1107769
Purified anti-CD16/32	Biolegend	Cat# 101302; RRID: AB_312801
Chemicals, Peptides, and Recombinant Proteins		
Dextran sulfate sodium	TdB Consultancy	Cat# 9011-18-1
Calbryte 590 AM	AAT bioquest	Cat# 20510
Noradrenaline bitartrate	Tocris	Cat# 5169
Nadolol	Sigma Aldrich	Cat# N1892
Phentolamine	Sigma Aldrich	Cat# P7547
LPS	Sigma Aldrich	Cat# L4391
666-15	Tocris	Cat# 5661
6-OHDA	Sigma Aldrich	Cat# H4381
Electrophoretic Tissue Clearing Solution	Logus biosystems	Cat# C13001
Critical Commercial Assays		
Lamina propria dissociation kit	Miltenyi Biotec	Cat# 130-097-410
Anti- CD45 microbeads	Miltenyi Biotec	Cat# 130-052-301; RRID: AB_2877061
Anti- CD31 microbeads	Miltenyi Biotec	Cat# 130-097-418; RRID: AB_2814657
Noradrenaline ELISA kit	IBL-America	Cat# IB89537
X-CLARITY™ Hydrogel Solution Kit	Logus biosystems	Cat# C1310X
In situ cell death detection kit	Roche	Cat# 11684795910
Experimental Models: Organisms/Strains		
Mouse: TH-Cre (B6129X1-Th < tm1(Cre)Te > /Kieg)	EMMA	Cat# EM:00254; RRID: IMSR_EM:00254
Mouse: DAT-Cre (B6.SJL-Slc6a3tm1.1(cre)Bkmn/J)	Jackson Laboratory	Cat# 006660; RRID: IMSR_JAX:006660
Mouse: SERT-Cre mice (B6.FVB(Cg)-Tg(Slc6a4-cre)ET33Gsat/Mmucd)	Jackson Laboratory	Cat# 014554; RRID: IMSR_JAX:014554
Mouse: ChR2 mice (Ai32(RCL-ChR2(H134R)/EYFP)	Jackson Laboratory	Cat# 024109; RRID: IMSR_JAX:024109
Mouse: GFP (C57BL/6-Tg(UBC-GFP)30Scha/J)	Jackson Laboratory	Cat# 004353; RRID: IMSR_JAX:004353
Mouse: C57BL/6JOlaHsd	ENVIGO	Cat # 2BL/606
Oligonucleotides		
GAPDH forward: 5¢-TGAAGCAGGCA TCTGAGGG-3¢ GAPDH reverse: 5¢-CGAAGGTGGAAGAGTGGGAG-3¢	UCSC Genome Browser	N/A
α4 forward: 5¢- CACAGCCACGGGTCGAA –3¢ α4 reverse: 5¢- AGGTCTGGTT TGGATTCTTTCTGA –3¢	UCSC Genome Browser	N/A
β7 forward: 5¢- GCTCTCTGTGGAAAT CTACGA –3¢ β7 reverse: 5¢- TCAC TCTGAAAAATCTCAGCG –3¢	UCSC Genome Browser	N/A

(*Continued on next page*)

Continued

REAGENT or RESOURCE	SOURCE	IDENTIFIER
CCL20 forward: 5¢- GGTGGCAAGCGTC TGCTC –3¢ CCL20 reverse: 5¢- GCC TGGCTGCAGAGGTGA –3¢	UCSC Genome Browser	N/A
CCL25 forward: 5¢-TGAAAGGAAGAA GTCAAACCATATGA –3¢ CCL25 reverse: 5¢- AGGGTGGCACTCCTCACG –3¢	UCSC Genome Browser	N/A
P-selectin forward: 5¢- ACGGGTGTTC TGTAGGAGGCAC –3¢ P-selectin reverse: 5¢- GTTGTTGGGCTCGTTGTCGG –3¢	UCSC Genome Browser	N/A
E-selectin forward: 5¢- CCAGAATGG CGTCATGGA –3¢ E-selectin reverse: 5¢- TAAAGCCCTCATTGCATTGA –3¢	UCSC Genome Browser	N/A
CCR7 forward: 5¢- GCTCCAGGCACG CAACTTT –3¢ CCR7 reverse: 5¢- GACTACCACCACGGCAATGA –3¢	UCSC Genome Browser	N/A
CCR9 forward: 5¢- AGGCCAAGAA GTCATCCAAGC –3¢ CCR9 reverse: 5¢- CCTTCGGAATCTCTCGCCAA –3¢	UCSC Genome Browser	N/A
VCAM-1 forward: 5¢- AGTTGGGGA TTCGGTTGTTC –3¢, VCAM-1 reverse: 5¢- CATTCCTTACCACCCCATTG –3¢	UCSC Genome Browser	N/A
MAdCAM-1 forward: 5¢- AGTTACTGTG CGCTGGACCTTGGCTCCTGGCGACC TGG-3¢ MAdCAM-1 reverse: 5¢- TCCTGGCGGCACTGGAACCAGCC-3¢	UCSC Genome Browser	N/A
ICAM-1 forward: 5¢- GAGAGTGG ACCCAACTGGAA-3¢, ICAM-1 reverse: 5¢- GCCACAGTTCTCAAAGCACA-3¢	UCSC Genome Browser	N/A
IL-6 forward: 5¢- TAGTCCTTCCTA CCCCAATTTCC –3¢ Il-6 reverse: 5¢- TTGGTCCTTAGCCACTCCTTC –3¢	UCSC Genome Browser	N/A
TNF-α forward: 5¢- CCTTTCACTCAC TGGCCCAA –3¢ TNF-α reverse: 5¢- AGTGCCTCTTCTGCCAGTTC –3¢	UCSC Genome Browser	N/A
IFN-γ forward: 5¢- GAGGTCAACAA CCCACAGGTC –3¢ IFN-γ reverse: 5¢- CGAATCAGCAGCGACTCCT-3¢	UCSC Genome Browser	N/A
IL-17 forward: 5¢- CCTCACACGAGG CACAAGTG –3¢ IL-17 reverse: 5¢- CTCTCCCTGGACTCATGTTTGC –3¢	UCSC Genome Browser	N/A
IL-12 forward: 5¢- AAGCTGCATCCTG CTTCAC –3¢ IL-12 reverse: 5¢- GATAGCCCATCACCCTGTTGA –3¢	UCSC Genome Browser	N/A
IL-21 forward: 5¢- TGCTAGCTCCAGC CTCAGTCT –3¢ IL-21 reverse: 5¢- TTAAGTGCTGAACTTGTTTGGATTG –3¢	UCSC Genome Browser	N/A
TGF-β forward: 5¢- AAACGGAAGCG CATCGAA –3¢ TGF-β reverse: 5¢- GGGACTGGCGAGCCTTAGTT –3¢	UCSC Genome Browser	N/A
IL-10 forward: 5¢- GCTCTTACTGACT GGCATGAG –3¢ IL-10 reverse: 5¢- CGCAGCTCTAGGAGCATGTG –3¢	UCSC Genome Browser	N/A
IL-1β forward: 5¢- AACCTGCTGGT GTGTGACGTTC –3¢ IL-1β reverse: 5¢- CAGCACGAGGCTTTTTTGTTGT –3¢	UCSC Genome Browser	N/A

(*Continued on next page*)

Continued		
REAGENT or RESOURCE	SOURCE	IDENTIFIER
Software and Algorithms		
FlowJo software	FlowJo LLC.	Version 10.5.0
IMARIS software	Oxford Instruments	Version 8.1
MATLAB	MathWorks	Version R2018B
Igor Pro software	Wavematrics	Version 5.04B
EthoVision software	Noldus	Version 11.5 XT
Prism software	GraphPad	Version 8.0.1

RESOURCE AVAILABILITY

Lead contact
Further information and requests for resources and reagents should be directed to and will be fulfilled by the Lead Contact, Asya Rolls (rolls.asya@gmail.com).

Materials availability
This study did not generate new unique reagents.

Data and code availability
This study did not generate any datasets or codes.

EXPERIMENTAL MODEL AND SUBJECT DETAILS

Mice models
Mice were maintained under Specific-Pathogen-Free (SPF) conditions on a 12:12 h light cycle (lights on at 07:00). Mice were grouped housed throughout the experiments. All mice were more than 8 weeks at the onset of experiments, and the typical age was 8 to 12 weeks. Several transgenic mice were used in the experiments: TH-Cre mice (B6129X1-Th < tm1(Cre)Te > /Kieg; EMMA), DAT-Cre mice (B6.SJL-Slc6a3tm1.1(cre)Bkmn/J; Jackson Laboratory), and SERT-Cre mice (B6.FVB(Cg)-Tg(Slc6a4-cre)ET33Gsat/Mmucd; Jackson Laboratory) cross-bred with ChR2 mice (RCL-ChR2(H134R)/EYFP; Jackson Laboratory), GFP mice (C57BL/6-Tg(UBC-GFP)30Scha/J) and C57BL/6 mice. All mice were on the same C57BL/6 background. Littermates of the same sex were randomly assigned to experimental groups. For controls, littermates negative for TH-Cre, or SERT-Cre (lacking ChR2 expression) were used, except for ChR2/DAT mice which do not have a negative littermate control (the DAT-Cre strain is homozygote). Therefore, in the ChR2/DAT experiments, the control group was ChR2/DAT mice who underwent the same procedures without light stimulation, namely the rectal probe was inserted but not turned on. All experiments were performed in accordance with the National Institutes of Health Guide for the Care and Use of Laboratory Animals. All procedures and protocols were approved by the Technion Administrative Panel of Laboratory Animal Care.

Primary colon cell culture
Colon-derived cells were isolated from the colon of mice (using the Lamina propria dissociation kit; Miltenyi Biotec). Then, the cells were incubated in Dulbecco's modified Eagle's medium (DMEM; Biological Industries) supplemented with 10% heat-inactivated fetal bovine serum (FBS; Biological Industries), 2mM L-glutamine (Biological Industries), 1mM sodium pyruvate (Biological Industries) and 1% PenStrep (Biological Industries) in a humidified atmosphere of 95% air and 5% CO2 at 37°C.

METHOD DETAILS

Optogenetic manipulation
Mice were anesthetized with isoflurane, and an optic fiber (with its polymeric outer cover removed) was connected to a blue LED source (460nm, Prizmatix) and inserted intra-rectally into the colon (as described in Figure 1E). Light was delivered for 30 min in alternating periods of 10 s of illumination with 1 ms pulses at 10 Hz, followed by a 20 s break. For controls, littermates negative for TH-Cre, or SERT-Cre (lacking ChR2 expression) were used undergoing the same experimental procedure, except for ChR2/DAT mice which do not have a negative littermate control (the DAT-Cre strain is homozygote). Therefore, in the ChR2/DAT experiments, the control group was ChR2/DAT mice who underwent the same procedures without light stimulation, namely the rectal probe was inserted but not turned on.

DSS-induced colitis model
Mice were administered 3% DSS (TdB Consultancy) in their drinking water for 7 consecutive days, and their weight, food and water consumption were monitored daily. During the DSS treatment period, the mice underwent daily optogenetic manipulation (30 min in

alternating periods of 10 s of illumination with 1 ms pulses at 10 Hz, followed by a 20 s break). Afterward, the mice were sacrificed, and the entire colon was removed from caecum to anus. The colon length was measured as a marker for inflammation, and the tissue was used for histological staining and scoring, mRNA analysis, immunohistochemistry staining, and flow cytometry analysis.

Histology staining and scoring

For histology staining, mice were sacrificed, and their colons were fixed in 4% paraformaldehyde (PFA) in PBS for 48 h, cryoprotected in 30% sucrose solution for another 48 h, and then frozen on dry ice. The colons were sliced in 10 μM sections and were mounted on super-frost slides (Fisherbrand). The slides were stained with Hematoxylin & Eosin (H&E). All images were taken using an automatic slide scanner (250 Flash III). The histological scoring was then performed by an investigator blinded to the treatment group of each sample. The components of the histological score were inflammatory infiltrate, goblet cell loss, crypt density, crypt hyperplasia, muscle thickening, submucosal inflammation, crypt abscess, and ulceration (as previously described in Koelink et al., 2018).

Immunohistochemical tissue analysis

Mice were sacrificed, and their colons were fixed in 4% PFA in PBS for 48 h, cryoprotected in 30% sucrose solution for another 48 h, and then frozen on dry ice. The colons were sectioned at 10 μm or 40 μm thickness. The 10 μm colon slices were mounted on super-frost slides (Fisherbrand). The 40 μm colon slices were rinsed twice in washing solution (0.05% Tween20 in PBS) and permeabilized for 15 min in 0.5% Triton X-100 in PBS. The following antibodies were used: anti-TH (1:500; Abcam), anti-GFP (1:100; Abcam), anti-β3-tubulin (1:500; Abcam), anti-DAT (1:500; Abcam), anti-serotonin (1:500; Abcam), anti-SERT (1:500; Sigma), anti-CD45 (1:100, Biolegend), anti-CD31 (1:100, R&D), anti-Ki67 (1:500; Abcam) and TUNEL (Roche diagnostics). All images were acquired using an Axio imager M2 microscope (Carl Zeiss Inc. US), 4-channel Olympus XI81-ZDC confocal microscope, or a laser scanning confocal microscope (Zeiss LSM 880). The percentage of cells with nuclear staining (Ki67 or TUNEL staining) was calculated out of the total number of $CD45^+$ cells using IMARIS software (version 8.1).

CLARITY staining: For clearing of the colon, the X-CLARITY technology was used (Logos biosystem) according to the manufacturer's instructions. The colons were then incubated with the primary antibodies (β3-tubulin, TH, anti-GFP for ChR2 fluorescent marker) diluted 1:100 at 37 °C for 1 week. Then, the colons were rinsed in a washing solution (0.05% Tween20 in PBS) for 2 days, and incubated with the secondary antibodies at 37 °C for 1 week. Then, the colons were incubated in DAPI staining solution (Sigma) for 2 days at 37 °C. All images were acquired using a laser scanning confocal microscope (Zeiss LSM 880) and analyzed using IMARIS software (version 8.1).

Analysis of noradrenaline levels

Analysis of NA levels in the colon was performed using a mouse microdialysis apparatus (Instech, model 375/D/22QM) to collect fluid from the colon tissue before and after the optogenetic stimulation (following 30 min of optogenetic stimulation). Samples were collected using a CMA 71 mm probe and a flow syringe pump (Chemyx, Fusion 400). Afterward, each sample was diluted in PBS containing 0.01 N HCl, 1 mM EDTA, and 4 mM sodium metabisulfite. For the analysis of NA levels in the serum, whole blood from anesthetized mice was collected before and after the optogenetic stimulation (following 30 min of optogenetic stimulation), centrifuged for 15 min at 2000xg, and stored at −80°C until analysis. NA levels in the colon and serum were analyzed using a NA ELISA kit (IBL-America, US).

Calcium indicator

For calcium imaging, an AM form of the red fluorescent calcium indicator, Calbryte 590 (AAT Bioquest) with an excitation peak of 580 nm and an emission peak of 592 nm was used. The dye was dissolved in DMSO for a stock solution, and later diluted to a working solution in a buffer containing 125 mM NaCl, 25 mM $NaHCO_3$, 25 mM glucose, 3 mM KCL, 1.25 mM NaH_2PO_4, 2 mM $CaCl_2$, 1 mM $MgCl_2$ (pH 7.4), with 0.04% Pluronic acid. The colons were freshly removed from ChR2/TH mice and incubated with the dye at 37°C for 1 h, followed by incubation at room temperature for 15 min. The dye solution was replaced with a buffer containing 125 mM NaCl, 25 mM $NaHCO_3$, 25 mM glucose, 3 mm KCL, 1.25 mM NaH_2PO_4, 2 mM $CaCl_2$, 1 mM $MgCl_2$ (pH 7.4) and the colons were imaged by laser scanning confocal microscopy (Zeiss LSM 880), objective 20x/0.8 M27 at 3.36 Hz full-field scanning mode (image size 512x157 pixels). Image analysis was performed using MATLAB and Igor software (Wavematrics). The statistical significance was calculate using paired t test comparing the baseline and peak florescence intensity.

Flow cytometry

Mice were sacrificed, and their colons were collected. Colon samples were dissociated using the Lamina propria dissociation kit (Miltenyi Biotec). Cells were incubated with antibodies for 30 min at 4 °C then washed with FACS staining buffer (PBS containing 1% bovine serum albumin and 0.05% sodium azide). The following antibodies were used (from Biolegend, San Diego, CA, US unless stated otherwise): Alexa Flour 700-conjugated and BV605-conjugated anti-CD45 (30-F11), PE-conjugated anti γ-δ (GL3), Pacific blue (PB)-conjugated anti-TCRβ (H57-597), BV510-conjugated anti-IL-17 (TC11-18H10.1), APC-conjugated and PE-Cy7- conjugated anti-CD4 (GK1.5), AF488-conjugated anti-FOXP3 (150D), BV650-conjugated NKp46 (29A1.4), BV605-conjugated and PB-conjugated anti CD3 (145-2C11), PerCP-conjugated anti-CD8 (53-6.7), BV510-conjugated anti-CD127 (A7R34), PB-conjugated anti T-bet (4B10), PerCP-conjugated anti-GATA3 (16E10A23), PE-conjugated anti RORγ (B2D, eBioscience), BV605-conjugated

anti CD62L (MEL14), BV650-conjugated anti CD44 (IM7), PerCP-Cy5.5-conjugated anti-CD11b (M1/70), PECy7-conjugated CD11c (N418), BV605-conjugated anti-CD103 (2EF), BV510-conjugated anti LY6C (HK1.4), APC-conjugated anti-LY6G (1A8), BV650-conjugated anti CX3CR1 (5A011F11), PECy7-conjugated anti-CD19 (6D5), BV510-conjugated anti-CD27 (LG.3A10), BV650-conjugated anti-CD138 (281-2), PerCP-conjugated anti-IgG (poly4053), APC-conjugated anti-IgM (RMM-1), biotin-conjugated anti-IgA (RMA-1), FITC-conjugated anti-IgD (1126C.29), PE conjugated anti-ICAM-1 (YN1/1.7.4), AF488-conjugated and biotin-conjugated anti-MAdCAM-1 (MECA-367), PECy7-conjugated anti-CD31 (390), biotin-conjugated anti-CD69 (H1.2F3), PE-conjugated anti-TNF-α (MP6-XT22), biotin-conjugated anti-CD86 (GL-1), PE-conjugated anti-CD80 (16-10A1), AF488-conjugated streptavidin (Jackson, 016-540-084), PerCP-conjugated streptavidin (Jackson, 016-120-84), BV605-conjugated anti streptavidin, PE-conjugated phospho-CREB (Cell Signaling, Ser133), and Zombie NIR dye. The samples were resuspended in 200 μl of 1% PFA and analyzed by flow cytometry using a LSRFortessa cell analyzer and FlowJo software. Gating strategies were set on the basis of unstained samples.

Noradrenaline injection

NA hydrochloride (Sigma Aldrich) was dissolved in a saline solution. DSS-treated mice (3% in the drinking water) were injected daily IP with NA (5 mg/kg; as described previously in Zhang et al., 2018), or saline for 7 days. Their weights were monitored daily, and afterward the mice were sacrificed, and the entire colon was removed from caecum to anus. The colon length was measured as a marker of inflammation, and the tissue was used for histological staining and scoring.

Nadolol (β-adrenergic blocker) and Phentolamine (α-adrenergic blocker) injection

Nadolol (Sigma Aldrich) and Phentolamine (Sigma Aldrich) were dissolved in saline solution. DSS-treated ChR2/TH mice (3% in the drinking water) were injected daily IP with the Nadolol (5 mg/kg) and Phentolamine (10 mg/kg) for 7 days (controls were injected with saline). The mice were subjected to optogenetic manipulation, as described in Figure 1E, 15 min following the injection. Their weights were monitored daily, and afterward, the mice were sacrificed, and the entire colon was removed from caecum to anus. The colon length was measured as a marker for inflammation, and the tissue was used for histological scoring and flow cytometry analysis.

Ex vivo assays

To assess the effects of NA on colon endothelial cells, cells were isolated from the colon of mice (using the Lamina propria dissociation kit; Miltenyi Biotec). Then, the cells were incubated with 1 μg/mL LPS (Sigma) diluted in DMEM (Biological Industries) supplemented with 10% heat-inactivated FBS (Biological Industries), 2mM L-glutamine (Biological Industries), 1mM sodium pyruvate (Biological Industries) and 1% PenStrep (Biological Industries) in a humidified atmosphere of 95% air and 5% CO2 at 37°C for 1 h. Afterward, NA was added (0-1000 μM), and the cells were incubated overnight at 37°C. The cells were then stained with Zombie NIR dye, APC-conjugated anti CD45 (30-F11), PECy7-conjugated anti-CD31 (390), AF488-conjugated anti-MAdCAM-1 (MECA-367) and analyzed with a LSRFortessa cell analyzer and FlowJo software.

Colon-derived endothelial cell enrichment: To enrich colon endothelial cells, cells were isolated from the colon of mice (using the Lamina propria dissociation kit; Miltenyi Biotec). Then, endothelial cells were enriched using the CD45 depletion kit (via CD45 microbeads; Miltenyi Biotec), and the CD31 enrichment kit (via CD31 microbeads; Miltenyi Biotec). Then, the enriched colon-derived endothelial cells (~90% purity; Figure S5K) were incubated with 1 μg/mL LPS (Sigma) diluted in DMEM (Biological Industries) supplemented with 10% heat-inactivated FBS (Biological Industries), 2mM L-glutamine (Biological Industries), 1mM sodium pyruvate (Biological Industries) and 1% PenStrep (Biological Industries) in a humidified atmosphere of 95% air and 5% CO2 at 37°C for 1 h. Afterward, 100 μM NA was added, and the cells were incubated overnight at 37°C. The cells were then stained with Zombie NIR dye, APC-conjugated anti CD45 (30-F11), PECy7-conjugated anti-CD31 (390), AF488-conjugated anti-MAdCAM-1 (MECA-367) and analyzed with a LSRFortessa cell analyzer and FlowJo software.

Adrenergic receptors manipulations: To evaluate the effects of NA on MAdCAM-1 expression, cells were isolated from the colon of mice (via the Lamina propria dissociation kit; Miltenyi Biotec). Then, the cells were incubated with 1 μg/mL LPS (Sigma) diluted in DMEM (Biological Industries) supplemented with 10% heat-inactivated FBS (Biological Industries), 2mM L-glutamine (Biological Industries), 1mM sodium pyruvate (Biological Industries) and 1% PenStrep (Biological Industries) in a humidified atmosphere of 95% air and 5% CO2 at 37°C for 1 h. The cells were next incubated with an α adrenergic blocker (3 μg/mL Phentolamine; Sigma) or a β adrenergic blocker (3 μg/mL Nadolol; Sigma) for 20 min. Afterward, 100 μM NA was added, and the cells were incubated overnight at 37°C. The cells were then stained with Zombie NIR dye, APC-conjugated anti CD45 (30-F11), PECy7-conjugated anti-CD31 (390), AF488-conjugated anti-MAdCAM-1 (MECA-367) and analyzed with a LSRFortessa cell analyzer and FlowJo software.

Phospho-CREB: To assess the role of CREB phosphorylation in the effect of NA on endothelial MAdCAM-1 expression, cells were isolated from the colon of mice (using the Lamina propria dissociation kit; Miltenyi Biotec). The colon cells were incubated with 100 μM NA diluted in DMEM (Biological Industries) supplemented with 10% heat-inactivated FBS (Biological Industries), 2mM L-glutamine (Biological Industries), 1mM sodium pyruvate (Biological Industries) and 1% PenStrep (Biological Industries) in a humidified atmosphere of 95% air and 5% CO2 at 37°C for 30 min at 37°C. The cells were then stained with Zombie NIR dye, APC-conjugated anti CD45 (30-F11), PECy7-conjugated anti-CD31 (390), AF488-conjugated anti-MAdCAM-1 (MECA-367), PE-conjugated phospho-CREB (Ser133), and analyzed with a LSRFortessa cell analyzer and FlowJo software.

CREB inhibitor: To evaluate the importance of CREB as the intracellular mediator on MAdCAM-1 levels, cells were isolated from the colon of mice (using the Lamina propria dissociation kit; Miltenyi Biotec). The isolated cells were incubated with 1 μM 666-15 (CREB inhibitor) diluted in DMEM (Biological Industries) supplemented with 10% heat-inactivated FBS (Biological Industries),

2mM L-glutamine (Biological Industries), 1mM sodium pyruvate (Biological Industries) and 1% PenStrep (Biological Industries) in a humidified atmosphere of 95% air and 5% CO2 at 37°C overnight. The cells were next incubated with 1 μg/mL LPS (Sigma) for 1 h. Afterward, 100 μM NA was added, and the cells were incubated overnight at 37°C. The cells were then stained with Zombie NIR dye, APC-conjugated anti CD45 (30-F11), PECy7-conjugated anti-CD31 (390), AF488-conjugated anti-MAdCAM-1 (MECA-367) and analyzed with a LSRFortessa cell analyzer and FlowJo software.

Sympathetic denervation

ChR2/TH mice and their controls (negative littermates exposed to the light stimulation) were sympathetically denervated by two IP injections of 6-OHDA (150 mg/kg in 0.01% ascorbic acid in saline; Sigma) administered at 24 h intervals, as described previously (Ben-Shaanan et al., 2018). 5 days afterward, the mice were administered 3% DSS and daily optogenetic stimulation for 7 days. Their weights were monitored daily, and at the end of the experiment the mice were sacrificed, and the entire colon was removed from caecum to anus. The colon length was measured as a marker for inflammation, and the tissue was used for histological staining and scoring, and flow cytometry analysis.

Anti MAdCAM-1 treatment

ChR2/TH mice and their controls (negative littermates exposed to the light stimulation) were exposed to 3% DSS for 7 days, and 20 min before the daily optogenetic stimulation, they were injected IP with anti-MAdCAM-1 antibody (MECA-367) or an isotype control (100 μg/mouse in saline; BioXcell). Their weights were monitored daily, and at the end of the experiment the mice were sacrificed, and the entire colon was removed from caecum to anus. The colon length was measured as a marker of inflammation, and the tissue was used for histological staining and scoring, and flow cytometry analysis.

Activity monitoring

The locomotor response was tested in ChR2/TH mice and their controls (negative littermates exposed to the light stimulation) during the experimental protocol (7 days of 3% DSS with daily optogenetic activation). The locomotor activity was recorded by a centrally placed overhead camera in an open field arena (30cm X 30cm). Mice were placed into the arena for 1 h, and the distance traveled by each mouse was assessed during the final 30 min using the 'distance' parameter in a video tracking system (EthoVision version 11.5 XT).

Quantitative RT-PCR

Colons and small intestines were lysed in TRI-Reagent (Sigma) and stored at −80 °C overnight. Total RNA was isolated according to the protocol supplied with the TRI-Reagent. Total RNA (0.1 μg) was reverse transcribed (RT) using the High-Capacity cDNA Reverse Transcription Kit (Applied Biosystems). Real-time PCR analysis was performed using an Applied Biosystems StepOnePlus Real-Time PCR System (Foster City, CA) in two independent experiments in duplicate, using the Fast SYBR Green Master Mix (Applied Biosystems). Dissociation analysis was performed at the end of each run to confirm the specificity of the reaction. The cycle conditions for real-time PCR were 95 °C for 20 s, followed by 40 cycles of 95 °C for 3 s and 60 °C for 30 s, and a melting curve stage (95 °C 15 s, 60 °C 1 min, 95 °C 15 s). Quantification of relative gene expression was performed according to the ΔΔ-CT method using StepOne Software 2.3 (Applied Biosystems), and the results expressed as fold difference ± SEM. The following primers were used:

GAPDH forward: 5¢- TGAAGCAGGCATCTGAGGG-3¢,
GAPDH reverse: 5¢- CGAAGGTGGAAGAGTGGGAG-3¢
α4 forward: 5¢- CACAGCCACGGGTCGAA −3¢,
α4 reverse: 5¢- AGGTCTGGTTTGGATTCTTTCTGA −3¢
β7 forward: 5¢- GCTCTCTGTGGAAATCTACGA −3¢,
β7 reverse: 5¢- TCACTCTGAAAAATCTCAGCG −3¢
CCL20 forward: 5¢- GGTGGCAAGCGTCTGCTC −3¢,
CCL20 reverse: 5¢- GCCTGGCTGCAGAGGTGA −3¢
CCL25 forward: 5¢-TGAAAGGAAGAAGTCAAACCATATGA −3¢,
CCL25 reverse: 5¢- AGGGTGGCACTCCTCACG −3¢
P-selectin forward: 5¢- ACGGGTGTTCTGTAGGAGGCAC −3¢,
P- selectin reverse: 5¢- GTTGTTGGGCTCGTTGTCGG −3¢
E-selectin forward: 5¢- CCAGAATGGCGTCATGGA −3¢,
E- selectin reverse: 5¢- TAAAGCCCTCATTGCATTGA −3¢
CCR7 forward: 5¢- GCTCCAGGCACGCAACTTT −3¢,
CCR7 reverse: 5¢- GACTACCACCACGGCAATGA −3¢
CCR9 forward: 5¢- AGGCCAAGAAGTCATCCAAGC −3¢,
CCR9 reverse: 5¢- CCTTCGGAATCTCTCGCCAA −3¢
VCAM-1 forward: 5¢- AGTTGGGGATTCGGTTGTTC −3¢,
VCAM-1 reverse: 5¢- CATTCCTTACCACCCCATTG −3¢
MAdCAM-1 forward: 5¢- AGTTACTGTGCGCTGGACCTTGGCTCCTGGCGACCTGG-3¢
MAdCAM-1 reverse: 5¢- TCCTGGCGGCACTGGAACCAGCC-3¢

ICAM-1 forward: 5¢- GAGAGTGGACCCAACTGGAA-3¢,
ICAM-1 reverse: 5¢- GCCACAGTTCTCAAAGCACA-3¢
IL-6 forward: 5¢- TAGTCCTTCCTACCCCAATTTCC −3¢
Il-6 reverse: 5¢- TTGGTCCTTAGCCACTCCTTC −3¢
TNF-α forward: 5¢- CCTTTCACTCACTGGCCCAA −3¢
TNF-α reverse: 5¢- AGTGCCTCTTCTGCCAGTTC −3¢
IFN-γ forward: 5¢- GAGGTCAACAACCCACAGGTC −3¢
IFN-γ reverse: 5¢- CGAATCAGCAGCGACTCCT-3¢
IL-17 forward: 5¢- CCTCACACGAGGCACAAGTG −3¢
IL-17 reverse: 5¢- CTCTCCCTGGACTCATGTTTGC −3¢
IL-12 forward: 5¢- AAGCTGCATCCTGCTTCAC −3¢
IL-12 reverse: 5¢- GATAGCCCATCACCCTGTTGA −3¢
IL-21 forward: 5¢- TGCTAGCTCCAGCCTCAGTCT −3¢
IL-21 reverse: 5¢- TTAAGTGCTGAACTTGTTTGGATTG −3¢
TGF-β forward: 5¢- AAACGGAAGCGCATCGAA −3¢
TGF-β reverse: 5¢- GGGACTGGCGAGCCTTAGTT −3¢
IL-10 forward: 5¢- GCTCTTACTGACTGGCATGAG −3¢
IL-10 reverse: 5¢- CGCAGCTCTAGGAGCATGTG −3¢
IL-1β forward: 5¢- AACCTGCTGGTGTGTGACGTTC −3¢
IL-1β reverse: 5¢- CAGCACGAGGCTTTTTTGTTGT −3¢

Illustrations

BioRender was used to design some of the illustration throughout the manuscript (https://biorender.com).

QUANTIFICATION AND STATISTICAL ANALYSIS

Results are illustrated as mean ± SEM. Graphs show data from at least two independent repeats. Significance was defined as $p < 0.05$. Statistical analysis was conducted using Prism7 (GraphPad Software). The specific statistical tests, exact value of n, what n represents, definition of center, and dispersion and precision of measures are mentioned in the figure legends.

Immunity, Volume *54*

Supplemental information

Optogenetic activation of local colonic sympathetic innervations attenuates colitis by limiting immune cell extravasation

Maya Schiller, Hilla Azulay-Debby, Nadia Boshnak, Yehezqel Elyahu, Ben Korin, Tamar L. Ben-Shaanan, Tamar Koren, Maria Krot, Fahed Hakim, and Asya Rolls

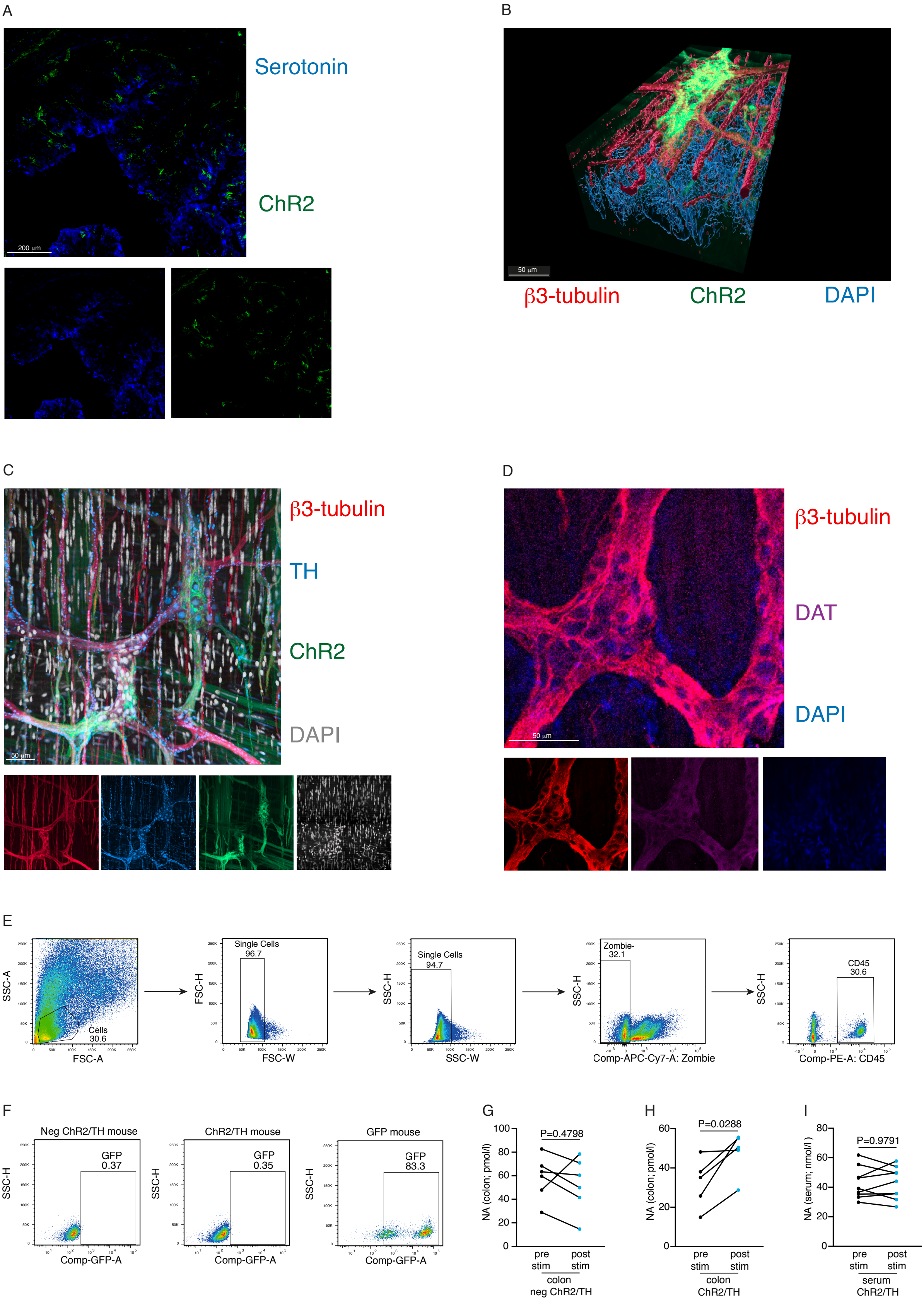

A
Serotonin
ChR2
200 µm
B
50 µm
β3-tubulin
ChR2
DAPI
C
β3-tubulin
TH
ChR2
DAPI
50 µm
D
β3-tubulin
DAT
DAPI
50 µm
E
SSC-A
FSC-A
Cells
30.6
FSC-H
FSC-W
Single Cells
96.7
SSC-H
SSC-W
Single Cells
94.7
SSC-H
Comp-APC-Cy7-A: Zombie
Zombie-
32.1
SSC-H
Comp-PE-A: CD45
CD45
30.6
F
Neg ChR2/TH mouse
GFP
0.37
ChR2/TH mouse
GFP
0.35
GFP mouse
GFP
83.3
SSC-H
Comp-GFP-A
G
NA (colon; pmol/l)
P=0.4798
pre stim
post stim
colon
neg ChR2/TH
H
NA (colon; pmol/l)
P=0.0288
pre stim
post stim
colon
ChR2/TH
I
NA (serum; nmol/l)
P=0.9791
pre stim
post stim
serum
ChR2/TH

Figure S1: Characterization of the optogenetic activation of local sympathetic fibers in the colon. Related to Figure 1. (A) Expression of the markers serotonin (blue) and the ChR2 fluorescent marker (green) in the colon of ChR2/TH mice, demonstrating that ChR2 is not express serotonin^{+} cells. Scale bar=200 µm. **(B)** Expression of the markers β3-tubulin (red), the ChR2 fluorescent marker (green) and DAPI (blue) in the colon of ChR2/TH mice. The image was taken from a colon that underwent a clearing technique. Scale bar=50 µm. **(C)** Expression of the markers β3-tubulin (red), TH (blue), ChR2 fluorescent marker (green) and DAPI (grey) in the colon of ChR2/TH mice. The image was taken from a colon that underwent a clearing technique. Scale bar=50 µm. **(D)** Expression of the markers β3-tubulin (red), DAT (purple), and DAPI (blue) in the colon of C57BL/6 mice. Scale bar=50 µm. **(E)** Gating strategy for ChR2/TH mice, their transgene negative littermates, and GFP mice used to validate that CD45^{+} cells from the colons of ChR2/TH mice do not express the ChR2 fluorescent marker. **(F)** Representative image of flow cytometry analysis, showing the percentage of GFP^{+} cells (the fluorescent marker expressed by ChR2^{+} cells) out of CD45^{+} cells (gating strategy shown in S1E) in the colons of negative littermates who do not express the ChR2 channel, ChR2/TH, and GFP mice as a positive control group. This analysis demonstrates that the ChR2 fluorescent marker was not expressed by CD45^{+} cells in the ChR2/TH mice. **(G)** Representative set of raw data of NA levels measured in the colons of transgene negative littermates and **(H)** ChR2/TH mice before (pre-stim) and after (post-stim) optogenetic stimulation. Negative littermate's colon: N=6; ChR2/TH colon: N=5. **(I)** NA levels measured from the serum of ChR2/TH mice before (pre-stim) and after (post- stim) optogenetic stimulation. N=9. Mean ± SEM, as well as individual mice, are presented for each group. Student's paired t-test.

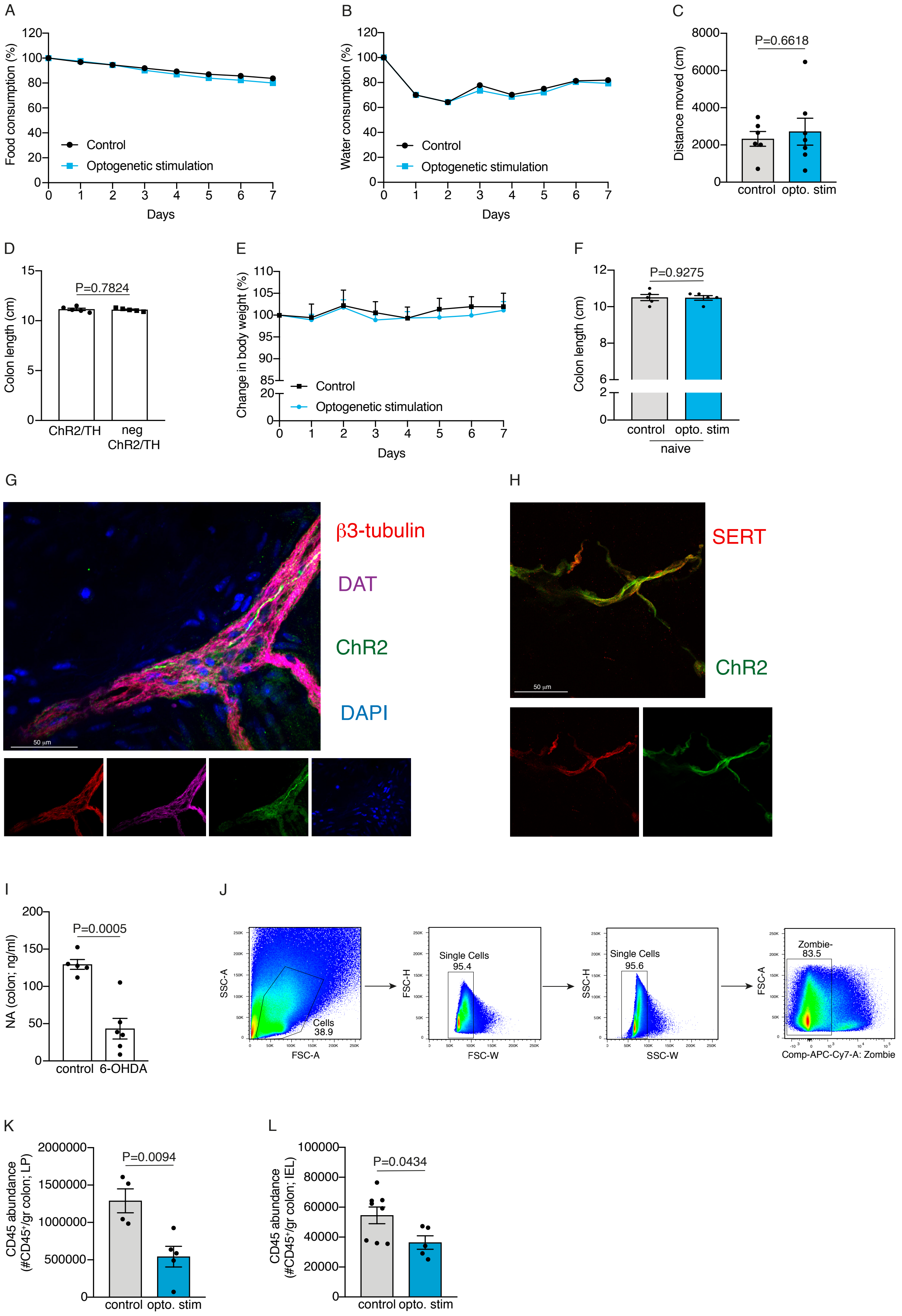
A
Food consumption (%)
Days
Control
Optogenetic stimulation
B
Water consumption (%)
Days
Control
Optogenetic stimulation
C
Distance moved (cm)
P=0.6618
control
opto. stim
D
Colon length (cm)
P=0.7824
ChR2/TH
neg ChR2/TH
E
Change in body weight (%)
Days
Control
Optogenetic stimulation
F
Colon length (cm)
P=0.9275
control
opto. stim
naive
G
β3-tubulin
DAT
ChR2
DAPI
50 μm
H
SERT
ChR2
50 μm
I
NA (colon; ng/ml)
P=0.0005
control
6-OHDA
J
SSC-A
FSC-A
Cells
38.9
FSC-H
FSC-W
Single Cells
95.4
SSC-H
SSC-W
Single Cells
95.6
FSC-A
Zombie-
83.5
Comp-APC-Cy7-A: Zombie
K
CD45 abundance (#CD45+/gr colon; LP)
P=0.0094
control
opto. stim
L
CD45 abundance (#CD45+/gr colon; IEL)
P=0.0434
control
opto. stim

Figure S2: Effects of optogenetic activation on immune abundance, behavioral and clinical traits in ChR2/TH mice, and the expression of ChR2 in ChR2/DAT and ChR2/SERT mice. Related to Figures 2 and 3. (A) Food and **(B)** water consumption by ChR2/TH mice and their controls (negative littermates exposed to light stimulation) during the 7 days of DSS administration and daily optogenetic activation. **(C)** Locomotion activity of ChR2/TH mice and their controls (negative littermates exposed to light stimulation) during the 7 days of DSS administration and daily optogenetic activation. The locomotion was calculated as the distance moved by each mouse (see methods). N=6, 7. **(D)** Colon length of ChR2/TH mice and their transgene negative littermates showing there is no difference in baseline colon length between the two strains. N=5, 5. **(E)** Percentage change in weight during the 7 days of daily optogenetic activation in naïve (not exposed to DSS) ChR2/TH mice and their controls (negative littermates exposed to light stimulation). N=5, 5. **(F)** Evaluation of colon length in naïve (not exposed to DSS) ChR2/TH mice and their controls (negative littermates exposed to light stimulation) following 7 days of daily optogenetic activation. N=5, 5. **(G)** Expression of the markers β3-tubulin (red), DAT (purple), ChR2 fluorescent marker (green) and DAPI (blue) in the colon of ChR2/DAT mice, demonstrating the expression of the ChR2 channel in DAT^+ neurons. Scale bar=50 μm. **(H)** Expression of the markers SERT (red) and the ChR2 fluorescent marker (green) in the colon of ChR2/SERT mice, demonstrating the expression of the ChR2 channel in $SERT^+$ cells. Scale bar=50 μm. **(I)** NA levels measured in the colons of mice injected IP with 6-OHDA (to ablate catecholamine neurons) and a vehicle-control group (injected with saline). N=5, 6. **(J)** Gating strategy for the analysis of immune cell abundance in the ChR2/TH mice and their controls. **(K)** Representative set of raw data of the abundance of immune cells (number of $CD45^+$ cells/gr colon) in the LP and **(L)** IEL layers of the colon from ChR2/TH mice and their controls following 7 days of 3% DSS and daily optogenetic activation. N=4, 5. Mean ± SEM as well as individual mice are presented for each group. Student's unpaired t-test.

A

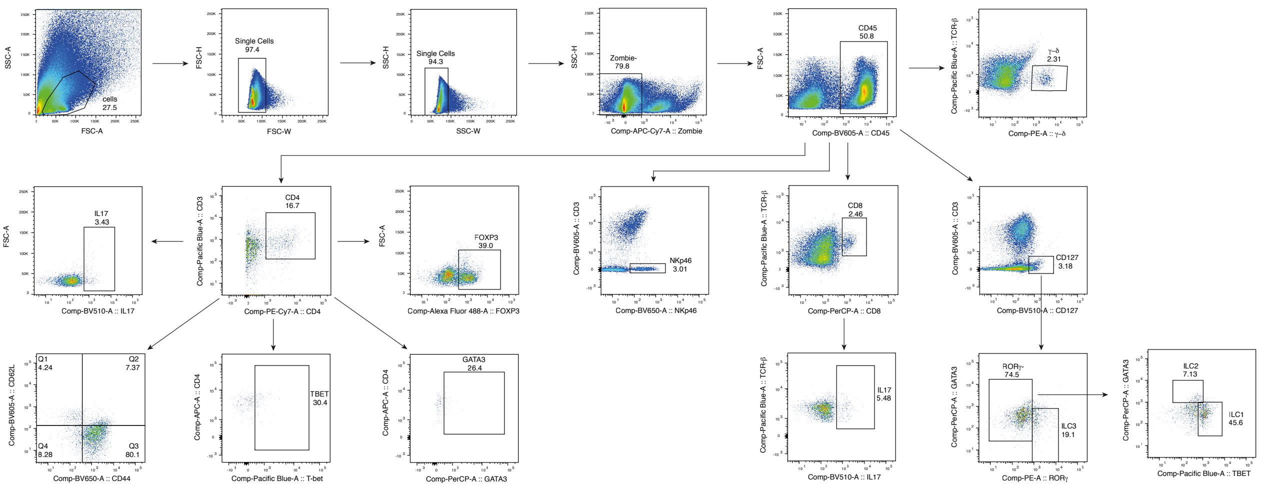
cells
27.5
FSC-A
SSC-A
Single Cells
97.4
FSC-W
FSC-H
Single Cells
94.3
SSC-W
SSC-H
Zombie-
79.8
Comp-APC-Cy7-A :: Zombie
CD45
50.8
Comp-BV605-A :: CD45
γ-δ
2.31
Comp-PE-A :: γ-δ
Comp-Pacific Blue-A :: TCR-β
IL17
3.43
Comp-BV510-A :: IL17
CD4
16.7
Comp-PE-Cy7-A :: CD4
Comp-Pacific Blue-A :: CD3
FOXP3
39.0
Comp-Alexa Fluor 488-A :: FOXP3
NKp46
3.01
Comp-BV650-A :: NKp46
Comp-BV605-A :: CD3
CD8
2.46
Comp-PerCP-A :: CD8
CD127
3.18
Comp-BV510-A :: CD127
Q1
4.24
Q2
7.37
Q4
8.28
Q3
80.1
Comp-BV650-A :: CD44
Comp-BV605-A :: CD62L
TBET
30.4
Comp-Pacific Blue-A :: T-bet
Comp-APC-A :: CD4
GATA3
26.4
Comp-PerCP-A :: GATA3
IL17
5.48
Comp-BV510-A :: IL17
RORγ
74.5
ILC3
19.1
Comp-PE-A :: RORγ
Comp-PerCP-A :: GATA3
ILC2
7.13
ILC1
45.6
Comp-Pacific Blue-A :: TBET

B

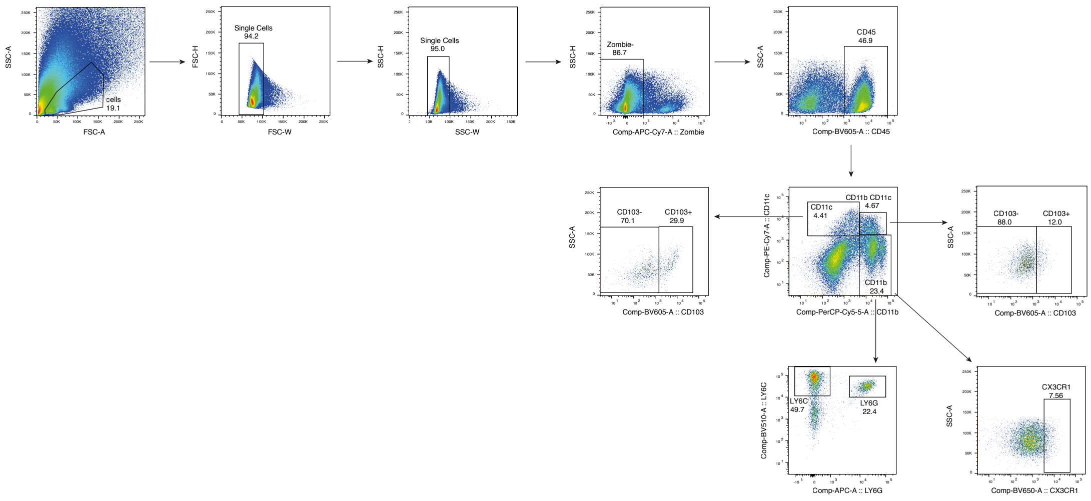
cells
19.1
FSC-A
SSC-A
Single Cells
94.2
FSC-W
FSC-H
Single Cells
95.0
SSC-W
SSC-H
Zombie-
86.7
Comp-APC-Cy7-A :: Zombie
CD45
46.9
Comp-BV605-A :: CD45
CD103-
70.1
CD103+
29.9
Comp-BV605-A :: CD103
CD11c
4.41
CD11b CD11c
4.67
CD11b
23.4
Comp-PerCP-Cy5-5-A :: CD11b
Comp-PE-Cy7-A :: CD11c
CD103-
88.0
CD103+
12.0
Comp-BV605-A :: CD103
LY6C
49.7
LY6G
22.4
Comp-APC-A :: LY6G
Comp-BV510-A :: LY6C
CX3CR1
7.56
Comp-BV650-A :: CX3CR1

C

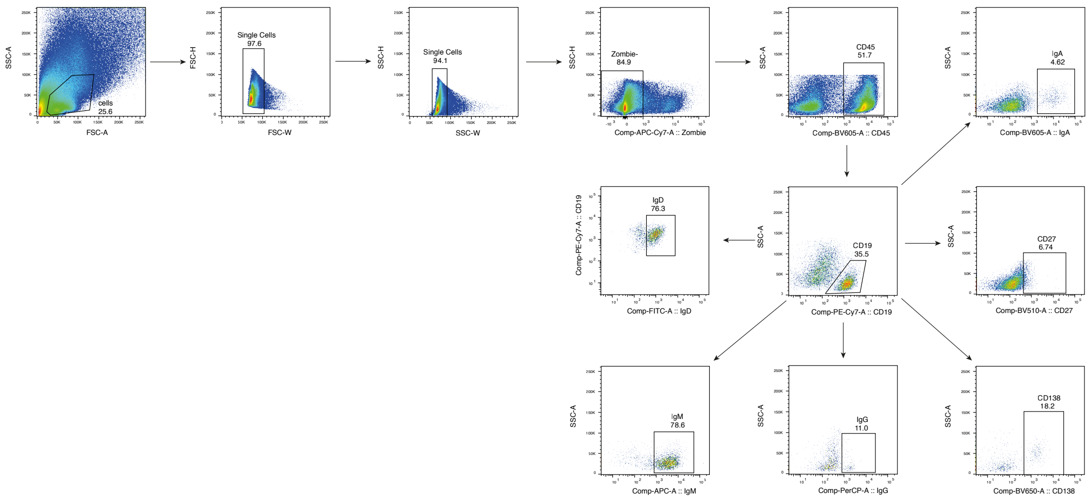
cells
25.6
FSC-A
SSC-A
Single Cells
97.6
FSC-W
FSC-H
Single Cells
94.1
SSC-W
SSC-H
Zombie-
84.9
Comp-APC-Cy7-A :: Zombie
CD45
51.7
Comp-BV605-A :: CD45
IgA
4.62
Comp-BV605-A :: IgA
IgD
76.3
Comp-FITC-A :: IgD
Comp-PE-Cy7-A :: CD19
CD19
35.5
Comp-PE-Cy7-A :: CD19
CD27
6.74
Comp-BV510-A :: CD27
IgM
78.6
Comp-APC-A :: IgM
IgG
11.0
Comp-PerCP-A :: IgG
CD138
18.2
Comp-BV650-A :: CD138

Figure S3: Gating strategy for immune subpopulations analysis in ChR2/TH mice following optogenetic activation. Related to Figure 3. Gating strategy of **(A)** T cells, NK cells, ILCs **(B)** myeloid, dendritic cells and **(C**) B cells in the colon of ChR2/TH mice and their controls (negative littermates exposed to light stimulation) following 7 days of 3% DSS and daily optogenetic activation.

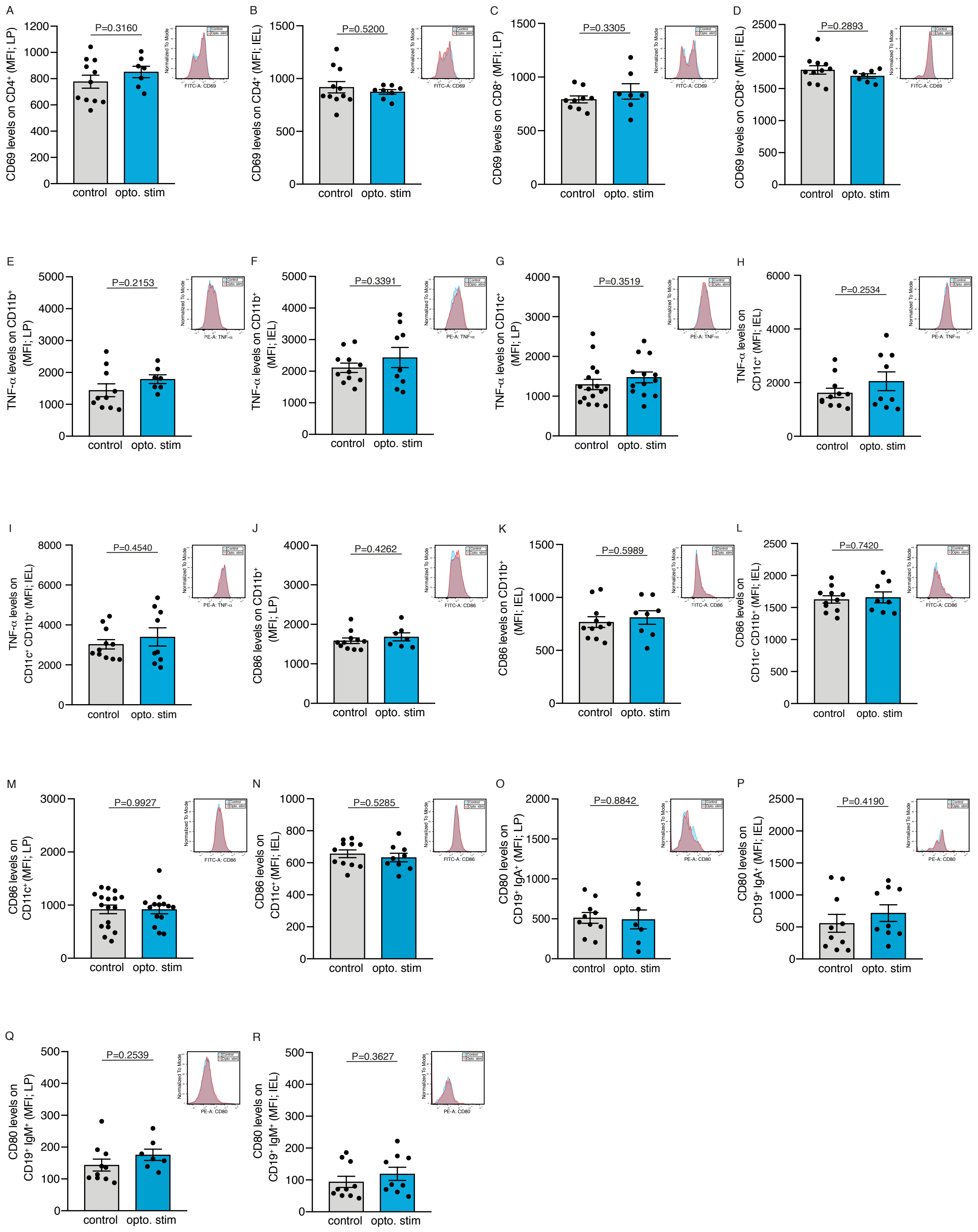
A
CD69 levels on CD4⁺ (MFI; LP)
P=0.3160
control
opto. stim
FITC-A: CD69
B
CD69 levels on CD4⁺ (MFI; IEL)
P=0.5200
control
opto. stim
FITC-A: CD69
C
CD69 levels on CD8⁺ (MFI; LP)
P=0.3305
control
opto. stim
FITC-A: CD69
D
CD69 levels on CD8⁺ (MFI; IEL)
P=0.2893
control
opto. stim
FITC-A: CD69
E
TNF-α levels on CD11b⁺ (MFI; LP)
P=0.2153
control
opto. stim
PE-A: TNF-α
F
TNF-α levels on CD11b⁺ (MFI; IEL)
P=0.3391
control
opto. stim
PE-A: TNF-α
G
TNF-α levels on CD11c⁺ (MFI; LP)
P=0.3519
control
opto. stim
PE-A: TNF-α
H
TNF-α levels on CD11c⁺ (MFI; IEL)
P=0.2534
control
opto. stim
PE-A: TNF-α
I
TNF-α levels on CD11c⁺ CD11b⁺ (MFI; IEL)
P=0.4540
control
opto. stim
PE-A: TNF-α
J
CD86 levels on CD11b⁺ (MFI; LP)
P=0.4262
control
opto. stim
FITC-A: CD86
K
CD86 levels on CD11b⁺ (MFI; IEL)
P=0.5989
control
opto. stim
FITC-A: CD86
L
CD86 levels on CD11c⁺ CD11b⁺ (MFI; IEL)
P=0.7420
control
opto. stim
FITC-A: CD86
M
CD86 levels on CD11c⁺ (MFI; LP)
P=0.9927
control
opto. stim
FITC-A: CD86
N
CD86 levels on CD11c⁺ (MFI; IEL)
P=0.5285
control
opto. stim
FITC-A: CD86
O
CD80 levels on CD19⁺ IgA⁺ (MFI; LP)
P=0.8842
control
opto. stim
PE-A: CD80
P
CD80 levels on CD19⁺ IgA⁺ (MFI; IEL)
P=0.4190
control
opto. stim
PE-A: CD80
Q
CD80 levels on CD19⁺ IgM⁺ (MFI; LP)
P=0.2539
control
opto. stim
PE-A: CD80
R
CD80 levels on CD19⁺ IgM⁺ (MFI; IEL)
P=0.3627
control
opto. stim
PE-A: CD80

Figure S4: Flow cytometry analysis of functional markers in ChR2/TH mice following optogenetic activation. Related to Figure 3. This analysis demonstrates: Expression levels of CD69 (MFI) on **(A)** LP $CD4^+$ (N=11, 7) **(B)** IEL $CD4^+$ (N=11, 8) **(C)** LP $CD8^+$ (N=9, 7). **(D)** IEL $CD8^+$ (N=11, 7). Expression levels of TNF-α (MFI) on **(E)** LP $CD11b^+$ (N=10, 7) **(F)** IEL $CD11b^+$ (N=11, 9) **(G)** LP $CD11c^+$ (N=16, 13) **(H)** IEL $CD11c^+$ (N=11, 9) **(I)** IEL $CD11c^+$ $CD11b^+$ (N=11, 9). Expression levels of CD86 (MFI) on **(J)** LP $CD11b^+$ (N=11, 7) **(K)** IEL $CD11b^+$ (N=11, 8) **(L)** IEL $CD11c^+$ $CD11b^+$ (N=11, 8) **(M)** LP $CD11c^+$ (N=17, 14) **(N)** IEL $CD11c^+$ (N=11, 9). Expression levels of CD80 (MFI) on **(O)** LP $CD19^+$ IgA^+ (N=10, 7) **(P)** IEL $CD19^+$ IgA^+ (N=10, 9) **(Q)** LP $CD19^+$ IgM^+ (N=10, 7) **(R)** IEL $CD19^+$ IgM^+ (N=10, 9). The analysis was performed on the colons of ChR2/TH mice and their controls (negative littermates exposed to light stimulation) following 7 days of 3% DSS and daily optogenetic activation. In each figure the right panel is a representative flow cytometry histogram. Mean ± SEM as well as individual mice are presented for each group. Student's unpaired t-test.

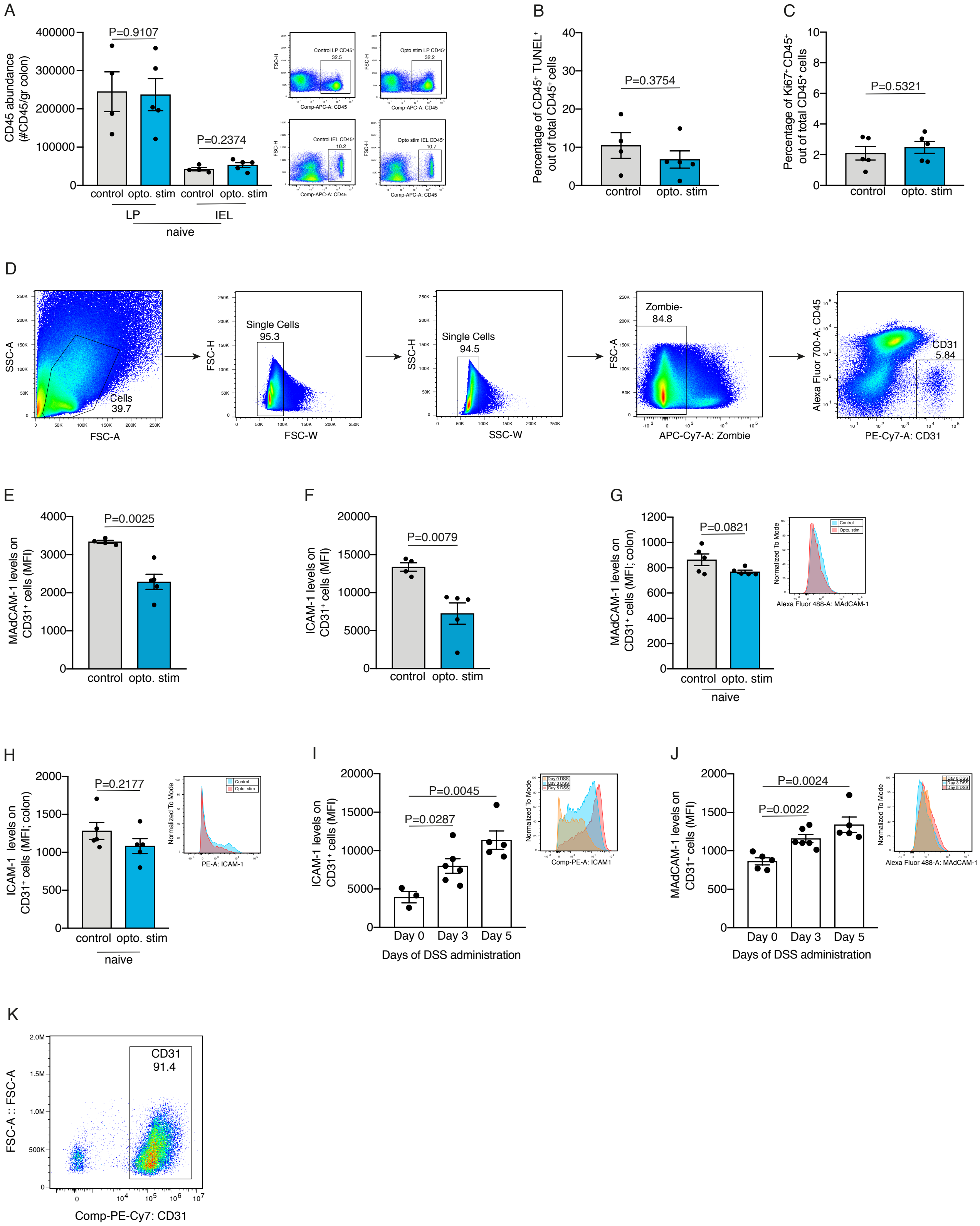
A
CD45 abundance (#CD45/gr colon)
400000
300000
200000
100000
0
P=0.9107
P=0.2374
control
opto. stim
control
opto. stim
LP
IEL
naive
Control LP CD45+
32.5
Opto stim LP CD45+
32.2
Control IEL CD45+
10.2
Opto stim IEL CD45+
10.7
Comp-APC-A: CD45
FSC-H
B
Percentage of CD45+ TUNEL+ out of total CD45+ cells
P=0.3754
control
opto. stim
C
Percentage of Ki67+ CD45+ out of total CD45+ cells
P=0.5321
control
opto. stim
D
SSC-A
FSC-A
Cells
39.7
FSC-H
FSC-W
Single Cells
95.3
SSC-H
SSC-W
Single Cells
94.5
FSC-A
APC-Cy7-A: Zombie
Zombie-
84.8
Alexa Fluor 700-A: CD45
PE-Cy7-A: CD31
CD31
5.84
E
MAdCAM-1 levels on CD31+ cells (MFI)
P=0.0025
control
opto. stim
F
ICAM-1 levels on CD31+ cells (MFI)
P=0.0079
control
opto. stim
G
MAdCAM-1 levels on CD31+ cells (MFI; colon)
P=0.0821
control
opto. stim
naive
Normalized To Mode
Alexa Fluor 488-A: MAdCAM-1
Control
Opto. stim
H
ICAM-1 levels on CD31+ cells (MFI; colon)
P=0.2177
control
opto. stim
naive
Normalized To Mode
PE-A: ICAM-1
Control
Opto. stim
I
ICAM-1 levels on CD31+ cells (MFI)
P=0.0045
P=0.0287
Day 0
Day 3
Day 5
Days of DSS administration
Normalized To Mode
Comp-PE-A: ICAM1
Day 0 DSS
Day 3 DSS
Day 5 DSS
J
MAdCAM-1 levels on CD31+ cells (MFI)
P=0.0024
P=0.0022
Day 0
Day 3
Day 5
Days of DSS administration
Normalized To Mode
Alexa Fluor 488-A: MAdCAM-1
Day 0 DSS
Day 3 DSS
Day 5 DSS
K
FSC-A :: FSC-A
Comp-PE-Cy7: CD31
CD31
91.4

Figure S5: Characterization of apoptosis and proliferation markers, immune abundance, MAdCAM-1 and ICAM-1 levels in ChR2/TH mice following optogenetic activation. Related to Figure 4. (A) Left: Representative set of raw data of abundance of immune cells (number of $CD45^+$ cells/gr colon) in the LP and IEL layers of the colon from naïve (not exposed to DSS) ChR2/TH mice and their controls (negative littermates exposed to light stimulation) following 7 days of daily optogenetic activation. Right: Representative flow cytometry plots demonstrating the percentage of $CD45^+$ population in the LP and IEL layers. N=4, 5. **(B)** Immunohistochemistry analysis of the percentage of $CD45^+$ cells expressing TUNEL (apoptosis marker) out of total $CD45^+$ cells in the colon of ChR2/TH mice and their controls (negative littermates exposed to light stimulation) following 7 days of 3% DSS and daily optogenetic activation. N=4, 5. **(C)** Immunohistochemistry analysis of the percentage of $CD45^+$ cells expressing Ki67 (proliferation marker) out of total $CD45^+$ cells in the colon of ChR2/TH mice and their controls (negative littermates exposed to light stimulation) following 7 days of 3% DSS and daily optogenetic activation. N=5, 5. **(D)** Gating strategy for the analysis of MAdCAM-1 and ICAM-1 expression level on endothelial cells ($CD31^+$ cells). **(E)** Representative set of raw data of flow cytometry analysis demonstrating the expression level of MAdCAM-1 on endothelial cells ($CD31^+$; indicated by MFI) in the colons of ChR2/TH mice and their controls following 7 days of 3% DSS and daily optogenetic activation. N=4, 5. **(F)** Representative set of raw data of flow cytometry analysis demonstrating the expression level of ICAM-1 on endothelial cells ($CD31^+$; indicated by MFI) in the colons of ChR2/TH mice and their controls following 7 days of 3% DSS and daily optogenetic activation. N=4, 5. **(G)** Left: Flow cytometry analysis of MAdCAM-1 expression on endothelial cells ($CD31^+$; indicated by MFI) in the colons of naïve (not exposed to DSS) ChR2/TH mice and their controls (negative littermates exposed to light stimulation), following 7 days of daily optogenetic stimulation. Right: Representative flow cytometry histogram demonstrating MAdCAM-1 expression level on $CD31^+$ cells. N=5, 5. **(H)** Left: Flow cytometry analysis of ICAM-1 expression on endothelial cells ($CD31^+$; indicated by MFI) in the colons of naïve (not exposed to DSS) ChR2/TH mice and their controls (negative littermates exposed to light stimulation), following 7 days of daily optogenetic stimulation. Right: Representative flow cytometry histogram demonstrating ICAM-1 expression level on $CD31^+$ cells. N=5, 5. **(I)** Left: Flow cytometry analysis of ICAM-1 expression level on endothelial cells ($CD31^+$; indicated by MFI) in the colons of C57BL/6 mice at different time points during 3% DSS administration. N=3, 6, 5. Right: Representative flow cytometry histogram demonstrating the ICAM-1 expression level on $CD31^+$

cells. **(J)** Left: Flow cytometry analysis of MAdCAM-1 expression level on endothelial cells ($CD31^+$; indicated by MFI) in the colons of C57BL/6 mice at different time points during 3% DSS administration. N=5, 6, 5. Right: Representative flow cytometry histogram demonstrating the MAdCAM-1 expression level on $CD31^+$ cells. **(K)** Representative image of flow cytometry analysis, showing the percentage of colon-derived endothelial cells ($CD31^+$) following endothelial enrichment (see methods). Mean ± SEM as well as individual mice are presented for each group. Student's unpaired t-test.
